# Supergene origin and maintenance in Atlantic cod

**DOI:** 10.1038/s41559-022-01661-x

**Published:** 2022-02-17

**Authors:** Michael Matschiner, Julia Maria Isis Barth, Ole Kristian Tørresen, Bastiaan Star, Helle Tessand Baalsrud, Marine Servane Ono Brieuc, Christophe Pampoulie, Ian Bradbury, Kjetill Sigurd Jakobsen, Sissel Jentoft

**Affiliations:** 1 Centre for Ecological and Evolutionary Synthesis (CEES), Department of Biosciences, University of Oslo, Oslo, Norway; 2 Department of Palaeontology and Museum, University of Zurich, Zurich, Switzerland; 3 Zoological Institute, Department of Environmental Sciences, University of Basel, Basel, Switzerland; 4 Marine and Freshwater Research Institute, Hafnarfjördur, Iceland; 5 Fisheries and Oceans Canada, St John’s, Newfoundland and Labrador, Canada; 6 Present Address: Natural History Museum, University of Oslo, Oslo, Norway

**Keywords:** Evolutionary biology, Population genetics, Phylogenomics, Comparative genomics

## Abstract

Supergenes are sets of genes that are inherited as a single marker and encode complex phenotypes through their joint action. They are identified in an increasing number of organisms, yet their origins and evolution remain enigmatic. In Atlantic cod, four megabase-scale supergenes have been identified and linked to migratory lifestyle and environmental adaptations. Here we investigate the origin and maintenance of these four supergenes through analysis of whole-genome-sequencing data, including a new long-read-based genome assembly for a non-migratory Atlantic cod individual. We corroborate the finding that chromosomal inversions underlie all four supergenes, and we show that they originated at different times between 0.40 and 1.66 million years ago. We reveal gene flux between supergene haplotypes where migratory and stationary Atlantic cod co-occur and conclude that this gene flux is driven by gene conversion, on the basis of an increase in GC content in exchanged sites. Additionally, we find evidence for double crossover between supergene haplotypes, leading to the exchange of an ~275 kilobase fragment with genes potentially involved in adaptation to low salinity in the Baltic Sea. Our results suggest that supergenes can be maintained over long timescales in the same way as hybridizing species, through the selective purging of introduced genetic variation.

## Abstract (web summary)

Atlantic cod carries four supergenes linked to migratory lifestyle and environmental adaptations. Using whole-genome sequencing, the authors show that the genome inversions that underlie the supergenes originated at different times and show gene flux between supergene haplotypes.

## Main

Many spectacular examples of phenotypic variation within species, such as mimicry patterns in butterflies^1^, social organization in ants^2^, plumage morphs in birds^3,4^ and floral types in plants^5^, are encoded by supergenes—tightly linked sets of genes that control a stable polymorphism in a Mendelian manner^6–9^. Even though supergenes have been known for nearly a century^10^, their origin remains a challenging question^9^. The emergence of supergenes requires beneficially interacting mutations in at least two genes and a reduction of recombination between these genes^7^. As a scenario in which these requirements can be met, recent research has pointed to chromosomal inversions arising in incompletely separated groups, such as interbreeding species or locally adapted populations that exchange migrants^11–13^. In these systems, beneficial interaction between mutations in different genes can come from their joint adaptation to the same environment, and these mutations can become linked if they are captured by the same inversion^6–9,14^. This linkage between mutations within inversions is the result of a loop formation that occurs during meiosis when chromosomes with the derived inverted haplotype pair with chromosomes with the ancestral haplotype arrangement. If a single crossover occurs within the loop region, the recombinant chromosomes are affected by duplications and deletions and are therefore unbalanced. The gametes carrying these unbalanced chromosomes are usually lethal^15,16^, so that the recombination rate between haplotypes with derived and ancestral arrangements appears reduced. However, crossovers between two haplotypes that both have the derived arrangement should not affect the viability of gametes. Since most inversions originate just once, in a single individual, the number of individuals in which haplotypes with the derived arrangement can successfully recombine is initially very low, increasing only as the derived arrangement becomes more frequent in the species. The origin of a supergene is therefore expected to be equivalent to a severe bottleneck (down to a single sequence) that affects part of the genome (the inversion region) in a part of the species (the carriers of the derived arrangement)^17^.

Once established, the maintenance of supergenes depends on the interaction of selection, drift, gene flow, mutation and recombination. The derived arrangement can be prevented from fixation—and the supergene can thus remain polymorphic—by frequency-dependent selection, by heterogeneous selection regimes in different populations or by recessive deleterious mutations that accumulate in the inversion region^7,11,13,18^. As mutations are added over time, the haplotypes with the ancestral and derived arrangements diverge from each other due to the suppression of recombination between them^18^. Owing to the reduced opportunity for recombination, mildly deleterious mutations are more likely to be fixed inside the inversion region than outside and can result in the accumulation of mutation load^18^. When this load becomes high within a supergene, it can lead to fitness decay for individuals carrying two copies of the same arrangement (that is, homokaryotypes)^19^. The accumulation of deleterious mutations, however, can be counteracted by two processes that allow gene flux (defined as the exchange of alleles during meiosis) between the two arrangements^2,9,19,20^: gene conversion and double crossover. Gene conversion is a process in which a homologous sequence is used as a template during the repair of a double-strand break, without requiring crossover with that homologous sequence^21,22^. The fragments copied through gene conversion are short, with lengths on the order of 50–1,000 base pairs (bp)^23,24^, and can have increased GC content due to biased repair of A–C and G–T mismatches^22,25^. Longer fragments can be exchanged through double crossovers when these occur within the loop formed by the two chromosomes. Either alone or in tandem, the two processes have the potential to erode differences between supergene haplotypes if their per-site rates are high relative to the mutation rate^26^. However, outside of model systems such as *Drosophila*, the rates of gene conversion and double crossovers are largely unknown.

In Atlantic cod (*Gadus morhua*), genomic regions with tight linkage over 4–17 megabases (Mbp) and strong differentiation between alternative haplotypes have been identified on linkage groups (LGs) 1, 2, 7 and 12 of the gadMor2 reference genome assembly^27–31^. The alternative haplotypes are associated with different life history strategies^29,32,33^ and environments^28,34–37^. One of the strongest of these associations is found between the haplotypes on LG 1 and migratory and stationary Atlantic cod ecotypes^29,35^. In the Northeast Atlantic, these ecotypes co-occur during the spawning season in March and April along the Norwegian coast, but they are separated throughout the rest of the year, with the migratory ecotype—the Northeast Arctic cod—returning to the Barents Sea^38^. With few exceptions, individuals carrying two copies of one of the haplotypes and heterozygous individuals are migratory, while individuals with two copies of the other haplotype are stationary^29,39,40^. Mating between the two ecotypes occurs at low frequency and explains the presence of individuals that are heterozygous for the LG 1 haplotypes as well as the very weak genetic separation outside of the four differentiated regions^29^. The differentiated region on LG 1 thus matches the definition of a supergene^6,33–35,41,42^. While certain genes from within this supergene have been proposed as candidate genes under selection, reliable identification of targets of selection remains difficult due to tight linkage among the nearly 800 genes within the supergene^32^. Similar to the different frequencies of LG 1 haplotypes between migratory and stationary ecotypes, one of the two alternative haplotypes on LG 2 is far more frequent in Atlantic cod from the Baltic sea than in the nearest North Atlantic populations and has been suggested to carry genes adapted to low salinity^28,37^. The alternative haplotypes on LGs 7 and 12 also differ in their frequencies among Atlantic cod populations, with one of the two haplotypes in each case being nearly absent in the southernmost populations, possibly in relation to adaptation to higher temperatures^41,43,44^. While the ‘supergene’ status of the haplotypes on LGs 2, 7 and 12 will depend on further investigations of their ecological roles, they, too, are commonly referred to by this term^33–35,41^, and we will call them supergenes hereafter.

Chromosomal inversions have long been suspected to be the cause of recombination suppression in the four supergenes in Atlantic cod^45^, but this has been confirmed only recently, first for the supergene on LG 1 with the help of detailed linkage maps for that LG (ref. ^32^) and then for the three other supergenes through comparison of long-read-based genome assemblies^41^. Age estimates have so far been reported only for the supergene on LG 1 (ref. ^32^), and conclusions about a possible joint origin of all four supergenes have therefore remained speculative. The role of introgression—genetic exchange through hybridization—in the origin of the Atlantic cod’s supergenes has so far also been uncertain. While introgression among codfishes (subfamily Gadinae) has been supported by one former study based on genome-scale sequence data^46^, these results were affected by the use of an incorrectly labelled specimen, susceptibility to reference bias and possibly incorrect outgroup choice in the application of *D*-statistics (Supplementary Note 1), and thus remain inconclusive regarding the occurrence of introgression.

Here we investigate the origin and maintenance of supergenes in Atlantic cod as follows. We generate a new long-read-based genome assembly for a stationary Atlantic cod individual from northern Norway as a complement to the existing genome assembly for a migratory individual from the Northeast Arctic cod population (gadMor2; ref. ^31^). Importantly, these two assemblies carry alternative haplotypes at each of the four supergenes. Through comparison of the two assemblies with each other and with an outgroup assembly, we corroborate the finding that inversions are the cause of recombination suppression for each supergene, pinpoint the chromosomal boundaries of the supergenes, and identify ancestral and derived arrangements. Using Bayesian time-calibrated phylogenetic analyses of newly generated and previously available genomic data, we show that at least some of the supergenes originated at different times. By applying *D*-statistics and sliding-window phylogenetic inference, we detect the occurrence of gene flux between haplotypes, through both gene conversion and double crossovers. Our results suggest that the long-term existence of supergenes may depend on genetic exchange between haplotypes to counter the accumulation of mutation load, and on selection acting on the exchanged sequences to maintain the separation of the two haplotypes.

## Results

### Presence of supergenes in Atlantic cod genomes

**Fig. 1:**
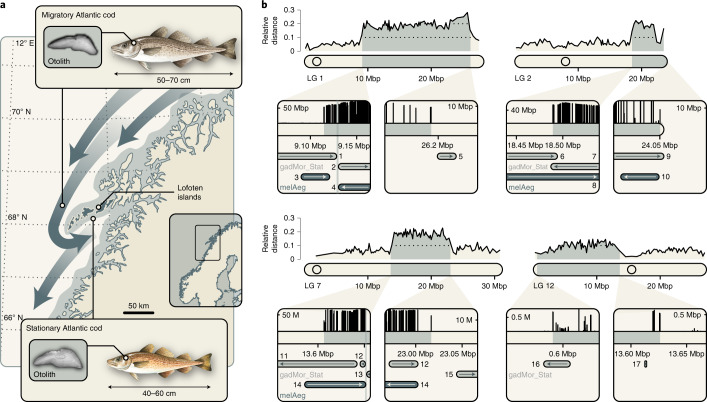
Four supergenes associated with megabase-scale chromosomal inversions in Atlantic cod. **a**, Migratory and stationary Atlantic cod seasonally co-occur along the coast of northern Norway and differ in total length and otolith measurements^38,52^. The distribution of stationary Atlantic cod is shaded in grey, whereas the seasonal movements of migratory Atlantic cod are indicated with dark-grey arrows. **b**, Pairwise sequence divergence between the gadMor2 and gadMor_Stat assemblies, relative to the sequence divergence of the haddock genome assembly (melAeg)^50^ in a three-way whole-genome alignment. The alignment coordinates are according to the gadMor2 assembly. LGs 1, 2, 7 and 12 are shown as rounded horizontal bars, on which circles indicate the approximate centromere positions^41^. Supergene regions are shaded in grey, and the beginning and end of each of these regions are shown in more detail in the insets below each LG. Each of these insets focuses on a section of 100 kbp around a supergene’s beginning or end. Shown in black above the bar representing that section is a per-SNP measure of LD, calculated as the sum of the distances between SNPs in high linkage (*R*^2^ > 0.8). On the basis of this measure, the grey shading on the bar illustrates the beginning or the end of high LD. Drawn below the scale bar are contigs of the gadMor_Stat and melAeg assemblies, in light grey and dark grey, respectively, that align well to the shown sections. The arrows indicate the alignment orientations of the contigs (forward or reverse complement), and the contigs are labelled with numbers as in Supplementary Table 3. In the first insets for LGs 1 and 7, the vertical bars indicate inferred inversion breakpoints, which are found up to 45 kbp (Table 1) after the onset of high LD. M, million. Fish drawings by Alexandra Viertler; otolith images by Côme Denechaud. Source data

To allow a comparison of genome architecture between migratory and stationary Atlantic cod, we performed PacBio and Illumina sequencing for a stationary Atlantic cod individual sampled in northern Norway, at the Lofoten islands (Fig. 1a). The resulting genome assembly (gadMor_Stat) consisted of 6,961 contigs with a contig N50 length of 121,508 bp and had a size of 565,431,517 bp, corresponding to approximately 87% of the estimated size of the Atlantic cod genome^31^. The assembly included 3,061 (84.1%) complete and 3,020 (83.0%) complete and single-copy genes out of 3,640 conserved BUSCO genes^47^ (Supplementary Table 1). When aligned to the gadMor2 reference genome assembly^31^, the gadMor_Stat assembly was highly similar on almost all gadMor2 LGs, with a pairwise sequence divergence of 0.40–0.53%. The exceptions to this were the four supergenes on LGs 1, 2, 7 and 12, which all showed an elevated sequence divergence of 0.66–1.29%. This confirmed that the gadMor_Stat and gadMor2 assemblies carried alternative supergene haplotypes on all four LGs (Supplementary Table 2). To determine the chromosomal boundaries of the regions of tight linkage associated with the supergenes, we investigated linkage disequilibrium (LD) on LGs 1, 2, 7 and 12 with a dataset of single-nucleotide polymorphisms (SNPs) for 100 Atlantic cod individuals. By quantifying the strength of linkage per SNP as the sum of the distances (in bp) with which the SNP is strongly linked (*R*^2^ > 0.8), we identified sharp declines of linkage marking the boundaries of all four supergenes (Fig. 1b and Table 1), as expected under the assumption of large-scale chromosomal inversions^48^.

**Table 1:** Tight linkage and chromosomal inversions in supergene regions in Atlantic cod

	High-LD region		Inversion region		Arrangement	
LG	Beginning	End	Beginning	End	gadMor2	gadMor_Stat
1	9,114,741	26,192,489	9,128,372–9,130,274	~26,100,000^a^	Derived	Ancestral
2	18,489,307	24,050,282	~18,490,000^b^	24,054,399–24,054,406^c^	Ancestral	Derived
7	13,606,502	23,016,726	13,651,003–13,652,432	23,002,424–23,043,967	Derived	Ancestral
12	589,105	13,631,347	607,782–662,878	13,386,293–13,614,908	Ancestral	Derived

All coordinates refer to the gadMor2 assembly^31^. Unless otherwise specified, the boundaries of inversion regions were determined on the basis of contig alignments (Supplementary Table 3).

^a^Comparison with the gadMor3 assembly^41^ (Supplementary Fig. 1) suggests that the actual end of the inversion region is misplaced in the gadMor2 assembly, between positions 18,890,477 and 18,900,044, and that the region between positions ~16,800,000 and ~18,900,000 in the gadMor2 assembly is instead located after position ~26,100,000.

^b^Due to repetitive sequences at the beginning of the inversion region, contigs mapping inside and outside of the region overlap between positions 18,487,151 and 18,494,225.

^c^This is the end of the LG in the gadMor2 assembly; however, comparison with the gadMor3 assembly suggests that the region from positions ~22,600,000 to ~23,700,000 in the gadMor2 assembly is incorrectly placed and is instead located at the end of the LG.

The presence of megabase-scale inversions on each of the four LGs was further supported by alignments of contigs from the gadMor_Stat assembly to the gadMor2 assembly, as we identified several contigs with split alignments of which one part mapped unambiguously near the beginning and another mapped near the end of a supergene (Supplementary Table 3). The positions of split contig alignments allowed us to pinpoint the inversion breakpoints on the four LGs with varying precision (Table 1). The most informative alignments were those near the beginnings of the supergenes on LGs 1 and 7, which in both cases placed the breakpoints within a window of approximately two kilobases (kbp). As also reported for inversions in *Drosophila*^49^, this precise placement of the inversion breakpoints revealed that they do not match the positions of LD onset exactly, but that they were located up to 45 kbp inside of the region of tight linkage (Fig. 1b and Table 1).

**Extended Data Fig. 1:**
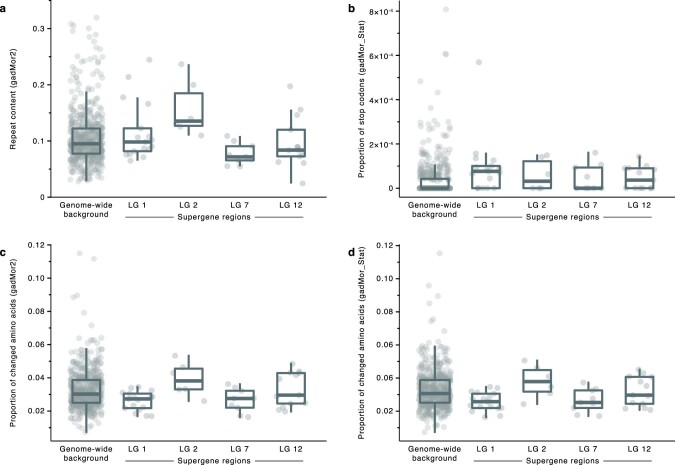
Repeat content and mutation load in Atlantic cod. Repeat content and mutation load were quantified in sliding windows along the gadMor2 assembly^31^. Windows had a length or 1 Mbp and were grouped into supergene regions and the remainder of the genome (*n* = 544, 17, 6, 9 and 13 for the genome-wide background and the four supergene regions, respectively). **a** Repeat content per window was quantified using the repeat annotation generated by Tørresen et al.^31^ for the gadMor2 assembly. **b–d** Mutation load was calculated based on the three-way genome alignment. As a first measure of mutation load, we quantified, per window, the proportion of stop codons among all codons in the gadMor_Stat sequences of the three-way alignment (**b**), according to gene annotation produced by Tørresen et al.^31^ for the gadMor2 assembly. As a second measure of mutation load, we calculated the proportions of amino acids that were changed, compared to the melAeg assembly, in the gadMor2 (**c**) and gadMor_Stat assemblies (**d**), according to the three-way alignment. Per supergene region, we tested for increased repeat content or mutation load compared to the genome-wide background; however, no measures were significantly increased at false discovery rate (FDR) 0.05 (one-sided *t*-test with measurements taken from distinct samples; *p* > 0.46; see Supplementary Table 21 for details). Box plots show the median as center line, box sizes indicate the first and third quartiles, and whiskers extend to the most extreme values or 1.5 × the interquartile range from the box limits. Source data

To determine which of the two genomes carries the derived arrangement in each case, we also aligned contigs from the long-read-based genome assembly of haddock (*Melanogrammus aeglefinus*; melAeg)^50^, a closely related outgroup within the subfamily Gadinae, to the gadMor2 assembly. We again identified split contig alignments mapping near the boundaries of the supergenes on LGs 1 and 7, indicating that for these supergenes, it is the gadMor2 genome that carries the derived arrangement (Fig. 1b, Table 1 and Supplementary Table 3). In contrast, a single contig of the melAeg assembly was clearly colinear to the gadMor2 assembly in a region that extended about 150 kbp in both directions from one of the ends of the supergene on LG 2, indicating that the derived arrangement on LG 2 is carried not by the gadMor2 genome but by the gadMor_Stat genome. For the supergene on LG 12, in contrast, no informative alignments were found; thus, our contig-mapping approach did not allow us to determine which of the two Atlantic cod genomes carries the derived arrangement on this LG (however, subsequent demographic analyses suggested that it is the gadMor_Stat genome; Supplementary Note 2). Repeat content and mutation load were not increased in supergene regions compared with the genome-wide background (Extended Data Fig. 1).

### Recent divergence among Atlantic cod populations

**Fig. 2:**
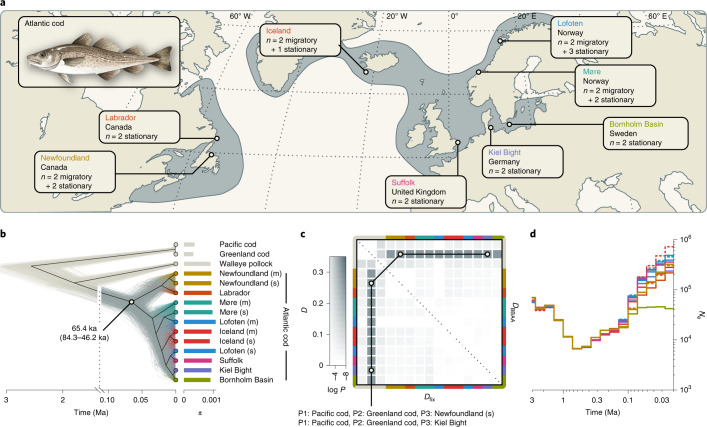
Divergence times, demography and gene flow among Atlantic cod populations. **a**, Geographic distribution and sampling locations of Atlantic cod in the North Atlantic. **b**, Tree of Atlantic cod populations and three outgroups (in beige; Pacific cod, Greenland cod and walleye pollock), inferred under the multispecies coalescent model from 1,000 SNPs sampled across the genome (excluding inversion regions). The thin grey and beige lines show individual trees sampled from the posterior distribution; the black line indicates the maximum-clade-credibility (MCC) summary tree. Estimates of *π* per population are indicated by bars to the right of the tips of the tree. **c**, Pairwise gene flow among Atlantic cod populations and introgression with outgroup species. Two versions of the *D*-statistic, *D*_BBAA_ and *D*_fix_, are shown above and below the diagonal, respectively. The colour codes on the axes indicate populations. The two trios (P1–P3) with the strongest signals are indicated, supporting introgression between Greenland cod and both the Kiel Bight and the stationary Newfoundland Atlantic cod populations with *D*_BBAA_ = *D*_fix_ = 0.250. **d**, Population sizes (*N*_e_) over time in Atlantic cod populations, estimated with Relate. For the Newfoundland, Møre, Iceland and Lofoten populations, migratory (m) and stationary (s) individuals were analysed separately; dashed lines are used for migratory populations. Source data

To estimate relationships and divergence times among Atlantic cod populations, we performed phylogenomic analyses for individuals from eight populations covering the species’ distribution in the North Atlantic (Fig. 2a and Supplementary Table 4), together with representatives of the three congeneric species, walleye pollock (*G. chalcogrammus*), Greenland cod (*G. ogac*) and Pacific cod (*G. macrocephalus*). In addition to the Atlantic cod individuals used for the gadMor2 and gadMor_Stat assemblies, we selected 22 individuals from the eight populations for which preliminary analyses had shown that each of them carried, at each of the four supergene regions, two copies of the same haplotypes (that is, they were homokaryotypic). For the sampling localities Newfoundland, Iceland, Lofoten and Møre, we discriminated between ‘migratory’ and ‘stationary’ individuals on the basis of whether they carried the same supergene haplotype on LG 1 as the gadMor2 genome or the same as the gadMor_Stat genome. At other localities, all individuals were considered stationary on the basis of the well-known migration patterns of Atlantic cod^51^. For the individuals from Lofoten and Møre, this classification could be confirmed by an analysis of their otoliths^52^, but otolith data were not available for the individuals from the other sampling localities.

On the basis of a dataset of 20,402,423 genome-wide biallelic SNPs, we estimated relationships and divergence times under the multispecies coalescent model, first only with data from outside of the supergene regions. In line with previous studies based on SNP arrays^27,35,53^, we found the primary divergence within Atlantic cod to separate the populations of the Northwest Atlantic from those of the Northeast Atlantic (including Iceland). We estimated these groups to have diverged around 65.4 thousand years ago (ka) (95% highest posterior density (HPD), 84.3–46.2 ka) but acknowledge that these results may underestimate the true divergence time because the applied model does not account for possible gene flow after divergence (Fig. 2b and Supplementary Fig. 2). The genetic diversity, quantified by *π*^54^, was comparable among the populations of both groups, ranging from 8.82 × 10^−4^ to 1.084 × 10^−3^ (Supplementary Table 5). Estimating changes in the population size (*N*_e_) over time for Atlantic cod revealed a Pleistocene bottleneck during which the population size of the common ancestor of all populations decreased from around 50,000 to 7,000 diploid individuals. The subsequent increase in population sizes occurred in parallel with the diversification of Atlantic cod populations and was experienced by all of them to a similar degree but less so by the Bornholm Basin population of the Baltic Sea (Fig. 2d).

**Extended Data Fig. 2:**
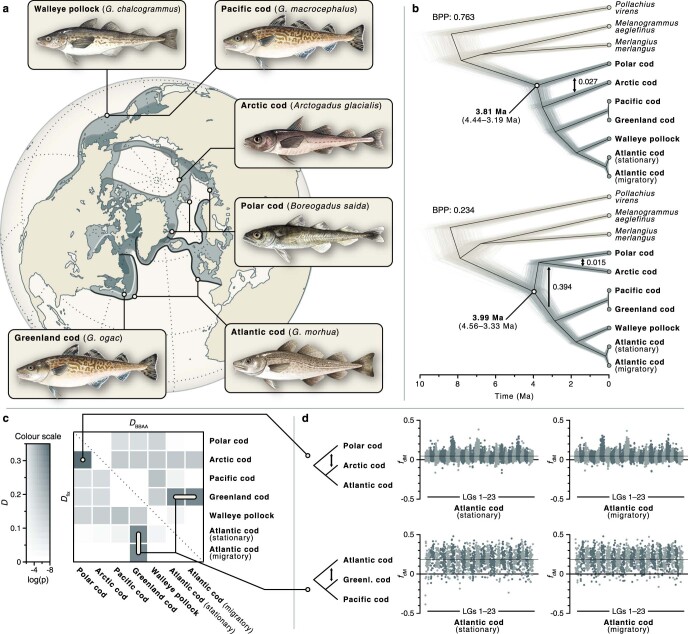
Divergence times and introgression among Gadinae. **a** Distribution ranges of species in the genera *Gadus*, *Arctogadus* and *Boreogadus*. Partially overlapping distribution ranges are shown in dark grey, with outline shades indicating the species (all distributions are shown separately in Supplementary Figure 7). **b** Species tree of the six species and three outgroups (*P*. *virens*, *M*. *aeglefinus* and *M*. *merlangus*; outgroups shown in beige), estimated under the isolation-with-migration model from 109 alignments with a total length 383,727 bp. The Bayesian analysis assigned 99.7% of the posterior probability to two tree topologies that differ in the position of Arctic cod and were supported with Bayesian posterior probabilities (BPP) of 0.763 and 0.234, respectively. Rates of introgression estimated in the Bayesian analysis are marked with arrows. Thin grey and beige lines show individual trees sampled from the posterior distribution; the black line indicates the maximum-clade-credibility summary tree, separately calculated for each of the two topologies. Of Atlantic cod, both migratory and stationary individuals were included. **c** Pairwise introgression among species of the genera *Gadus*, *Arctogadus* and *Boreogadus*. Introgression was quantified with the *D*-statistic. The heatmap shows two versions of the *D*-statistic, *D*_BBAA_ and *D*_fix_, above and below the diagonal, respectively. **d** Introgression across the genome. The *f*_dM_-statistic^103^ is shown for sliding windows in comparisons of three species. The top and bottom rows show support for introgression between polar cod and Arctic cod and between Atlantic cod and Greenland cod, respectively. Results are shown separately for the stationary and migratory Atlantic cod genomes. The mean *D*-statistic across the genome is marked with a thin solid line. See Supplementary Notes 3 and 4 for details and a discussion of these results. Fish drawings by Alexandra Viertler. Source data

Applied to the set of 20,402,423 SNPs, Patterson’s *D*-statistic revealed the occurrence of introgression between Greenland cod and Atlantic cod and showed that this signal of introgression is similar in all populations (*D*_BBAA_ = *D*_fix_ = 0.249, *P* < 10^−10^; Fig. 2c and Supplementary Tables 6 and 7). The *D*-statistic supported the same signal with an additional dataset that also included further outgroup species. This introgression signal was observed across all LGs, suggesting that the supergenes did not arise in Atlantic cod due to introgression from Greenland cod (Extended Data Fig. 2 and Supplementary Notes 3 and 4).

### Differing ages of supergenes

**Fig. 3:**
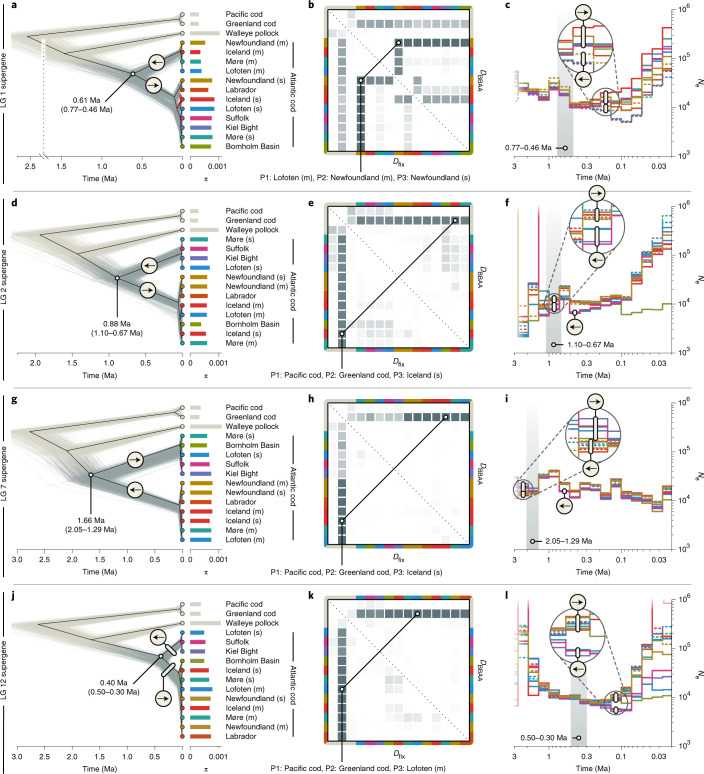
Divergence times, demography and gene flux within supergene regions. **a**,**d**,**g**,**j**, Trees of Atlantic cod populations and three outgroups (in beige; Pacific cod, Greenland cod and walleye pollock) inferred under the multispecies coalescent model from 1,000 SNPs sampled from the supergene regions on LGs 1 (**a**), 2 (**d**), 7 (**g**) and 12 (**j**). The thin grey and beige lines show individual trees sampled from the posterior distribution; the black line indicates the MCC summary tree. Within Atlantic cod, derived and ancestral arrangements are marked with forward and reverse arrows, respectively. Estimates of *π* per population within supergene regions are indicated by bars to the right of the tips of the tree. **b**,**e**,**h**,**k**, Pairwise signals of past gene flow among Atlantic cod populations and introgression with outgroup species within the supergene regions on LGs 1 (**b**), 2 (**e**), 7 (**h**) and 12 (**k**). Two versions of the *D*-statistic, *D*_BBAA_ and *D*_fix_, are shown above and below the diagonal, respectively. The colour codes on the axes indicate populations, ordered as in **a**,**d**,**g**,**j**, and the heatmap colours indicate *D*-statistics as in Fig. 2c. The trios (P1–P3) with the strongest signals of gene flux or introgression are indicated. **c**,**f**,**i**,**l**, Population sizes (*N*_e_) over time in Atlantic cod populations for the supergene regions on LGs 1 (**c**), 2 (**f**), 7 (**i**) and 12 (**l**). For the Newfoundland, Møre, Iceland and Lofoten populations, migratory (m) and stationary (s) individuals were analysed separately; dashed lines are used for migratory populations. The grey regions indicate the confidence intervals for the inferred age of the split between the two haplotypes (from **a**,**d**,**g**,**j**). Source data

**Fig. 4:**
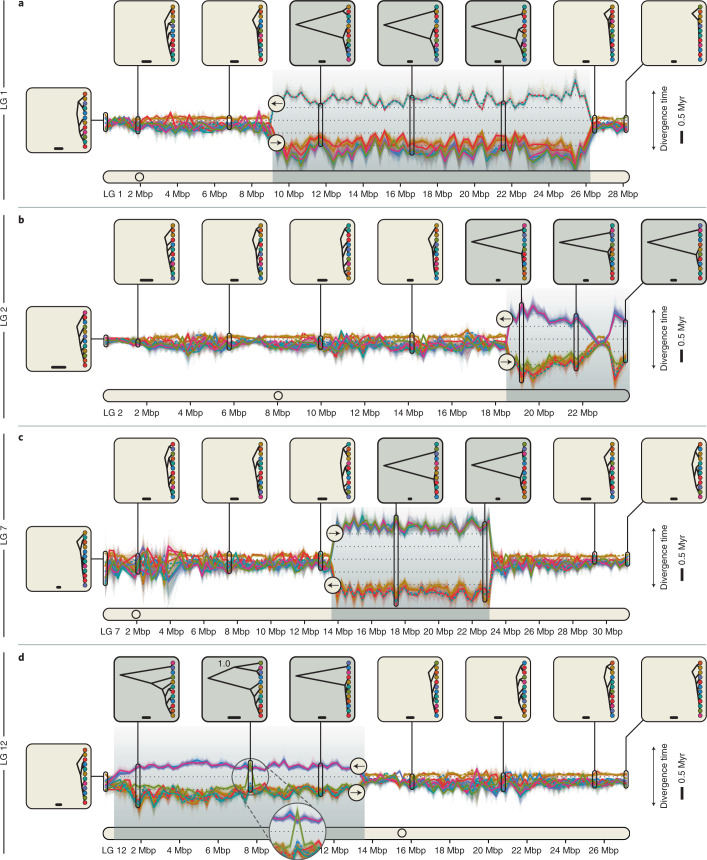
Divergence-time profiles for LGs with supergenes. **a**–**d**, Between-population divergence times along LGs 1 (**a**), 2 (**b**), 7 (**c**) and 12 (**d**), estimated from SNPs in sliding windows. Supergene regions are indicated by grey backgrounds. Along the vertical axis, the distance between two adjacent lines shows the time by which the corresponding populations have been separated on the ladderized population tree for a given window; both the scale bar and the dotted lines indicate a duration of 0.5 Myr. Examples of the population tree are shown in insets for eight selected windows. The scale bars in these insets indicate a branch length equivalent to 50,000 years. The node label in one inset in **d** indicates the support for the grouping of the Bornholm Basin population with three populations representing the derived arrangement (BPP, 1.0). Source data

To infer the ages of the supergenes on LGs 1, 2, 7 and 12, we applied phylogenomic analyses to SNPs from each supergene separately, extracted from the dataset of 20,402,423 biallelic SNPs. For each of the four supergenes, we recovered a deep divergence separating the haplotypes with ancestral and derived arrangements; however, the age estimates for this divergence differed widely among the supergenes, with mean age estimates of 0.61 million years ago (Ma) (95% HPD, 0.77–0.46 Ma) for the supergene on LG 1 (Fig. 3a), 0.88 Ma (95% HPD, 1.10–0.67 Ma) for the supergene on LG 2 (Fig. 3d), 1.66 Ma (95% HPD, 2.05–1.29 Ma) for the supergene on LG 7 (Fig. 3g) and 0.40 Ma (95% HPD, 0.50–0.30 Ma) for the supergene on LG 12 (Fig. 3j and Supplementary Fig. 3). Demographic analyses revealed past reductions in the population sizes of haplotypes with derived arrangements, in line with the expectation for inversion-based supergenes (Fig. 3c,f,i,l and Supplementary Note 2). The migratory individuals from Newfoundland, Iceland, Lofoten and Møre shared the same arrangement on all four LGs, and so did the stationary individuals from Lofoten, Suffolk and Kiel Bight (Fig. 4a,d,g,j). The genetic diversity of the haplotype with the derived arrangement was on average lower on LGs 1 and 12 but higher on LGs 2 and 7, compared with the haplotype with the ancestral arrangement (Fig. 4a,d,g,j and Supplementary Table 5).

### Gene flux between supergene haplotypes via gene conversion

Applied to the sets of supergene-specific SNPs, the *D*-statistic supported gene flux (and thus the occurrence of either gene conversion or double crossover) between haplotypes with derived and ancestral arrangements, particularly for the supergene on LG 1 and the geographically co-occurring migratory and stationary Newfoundland populations (*D*_BBAA_ = *D*_fix_ = 0.383, *P* < 10^−10^; Fig. 3b and Supplementary Tables 8 and 9). To test whether gene conversion could be the cause of this gene flux occurring between the Newfoundland populations, we tested for the GC bias expected from this process^24,25^, comparing the GC content of 7,283 sites shared between the two Newfoundland populations (‘ABBA’ sites) with that of 47,853 sites shared between the migratory Newfoundland population and other migratory populations (‘BBAA’ sites). The mean GC content of the former, 0.482, is significantly higher than that of the latter, 0.472 (one-sided *t*-test with measurements taken from distinct samples; *t* = −4.74, d.f. = 27,833, *P* < 10^−5^), supporting gene conversion as an agent of gene flux between haplotypes with derived and ancestral arrangements in the Newfoundland populations. For the supergenes on LGs 2, 7 and 12, the *D*-statistic indicated only comparatively weak signals of gene flux between derived and ancestral arrangements (*D* ≤ 0.2, *P* ≥ 10^−4^; Fig. 3e,h,k and Supplementary Tables 10–15).

### Double crossover revealed by divergence-time profiles

**Fig. 5:**
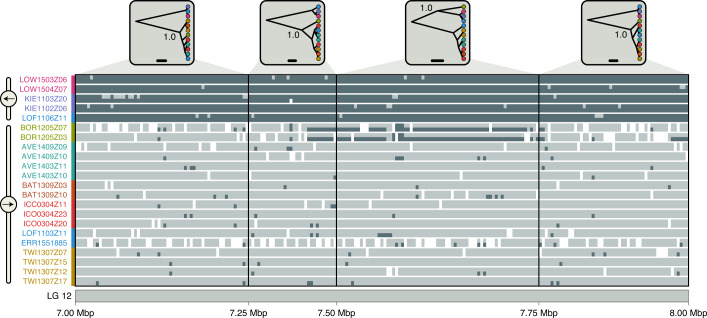
Ancestry painting for part of the supergene on LG 12. The ancestry painting^71,113^ shows genotypes at 219 haplotype-informative sites between positions 7 and 8 Mbp on LG 12, within the supergene on that LG. For each of 22 Atlantic cod individuals, homozygous genotypes are shown in dark or light grey, while heterozygous genotypes are illustrated with a light-grey top half and a dark-grey bottom half; white indicates missing genotypes. We selected as haplotype-informative sites those that have less than 10% missing data and strongly contrasting allele frequencies (≥0.9 in one group and ≤0.1 in the other) between the group carrying the derived arrangement (individuals from Suffolk, Kiel Bight and stationary Lofoten) and the group carrying the ancestral arrangement (individuals from the Møre, Labrador, Iceland, migratory Lofoten and Newfoundland populations). The four insets at the top show population trees inferred from SNPs; the node labels in these insets indicate Bayesian support for the grouping of the Bornholm Basin population with either the derived or the ancestral arrangement. Source data

**Extended Data Fig. 3:**
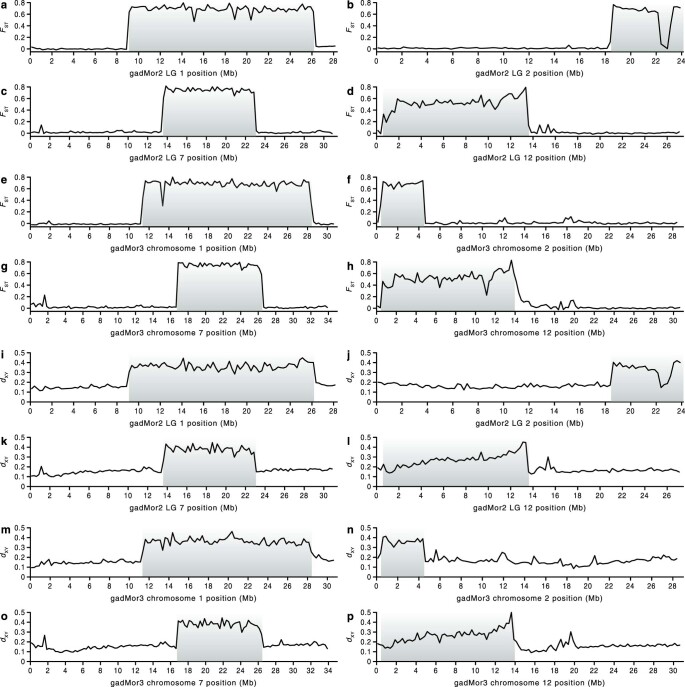
Measures of differentiation and divergence for linkage groups with supergenes. As a complement to the patterns of temporal divergence shown in Figure 4, differentiation and divergence across linkage groups with supergenes were also quantified as *F*_st_ (**a**-**h**) and *d*_xy_ (**i**-**p**). As in Figure 4, the two measures were calculated in sliding windows with a length of 250 kbp, for predefined groups of populations that separated those with the ancestral and derived supergene orientations. Both measures are plotted across linkage groups of the gadMor2 assembly (**a**-**d**, **i**-**l**) and chromosomes of the newer gadMor3^41^ (**e**-**h**, **m**-**p**) assembly. Note that gadMor3 chromosome 2 is inverted relative to gadMor2 LG 2 (see Supplementary Figure 1). Comparable results were obtained by Barth et al.^37^ for gadMor2 LGs 2, 7 and 12, using a different dataset and shorter window sizes of 50 and 100 kbp. Source data

To explore whether divergence times between the two arrangements per supergene are homogeneous across supergene regions, we repeated phylogenomic inference in sliding windows of 250 kbp along all LGs. We expected that if any gene flux between supergene haplotypes proceeded via double crossovers, its effect should be less pronounced near the inversion breakpoints and stronger towards their centres, which could generate U-shaped divergence profiles for supergene regions^20,55,56^. Contrary to this expectation, the divergence-time profiles were relatively homogeneous from beginning to end, particularly for the supergenes on LGs 1, 7 and 12 (Fig. 4a,c,d and Supplementary Fig. 4), suggesting either that double crossovers are rare within these supergenes or that sequences exchanged through double crossovers are frequently purged from the recipient haplotypes. As the supergene on LG 1 is known to include two adjacent inversions of roughly similar size^32^, our results also suggested a similar age and possibly a joint origin for both of these inversions. The divergence-time profile for the supergene on LG 2 appeared consistent with the expectation of a U-shaped pattern; however, comparison with the recently released gadMor3 assembly^41^ showed that the end of this LG may be misassembled (Supplementary Fig. 1). Additional analyses of sequence differentiation (*F*_ST_)^57^ and sequence divergence (*d*_XY_) in windows across LGs 1, 2, 7 and 12, performed with both the gadMor2 and gadMor3 assemblies as references, confirmed this assumption as well as the absence of U-shaped patterns for the four regions (Extended Data Fig. 3). However, the divergence-time profile for LG 12 revealed a single window within the supergene in which the otherwise clear separation between the groups carrying the alternative arrangements was interrupted: unlike in all other windows within this supergene, the Bornholm Basin population grouped (Bayesian posterior probability (BPP), 1.0) with the three populations representing the derived arrangement (Suffolk, Kiel Bight and stationary Lofoten) in the window for positions 7.50–7.75 Mbp (Fig. 4d). To investigate the genotypes of the two sampled Bornholm Basin individuals within this region in more detail, we identified 219 haplotype-informative sites between positions 7 and 8 Mbp on LG 12 and found that these individuals were both heterozygous at these sites, for a region of ~275 kbp between positions 7,478,537 bp and 7,752,994 bp (Fig. 5). The two individuals from the Bornholm Basin population thus carried a long sequence from the haplotype with the derived arrangement even though they were otherwise clearly associated with the ancestral arrangement. As the length of this introduced sequence was far longer than the 50–1,000 bp expected to be copied per gene-conversion event^23,24^, it strongly supports double crossover between the two haplotypes of the LG 12 supergene. The region covered by the introduced sequence contains 24 predicted genes (Supplementary Table 16), including a cluster of three vitellogenin genes, out of a total of four vitellogenin genes found in the gadMor2 genome. These genes are known to influence the buoyancy of fish eggs^58–61^ and could thus be targets of selection in Atlantic cod populations in the brackish Baltic Sea^28^.

## Discussion

Through comparison of long-read-based genome assemblies for migratory and stationary Atlantic cod individuals, we corroborated the finding that chromosomal inversions underlie all four supergenes in Atlantic cod^41^. The inversion breakpoints do not coincide exactly with the boundaries of the supergenes but lie up to 45 kbp inside them, in agreement with findings reported for *Drosophila* that suggested that recombination suppression can extend beyond inversion breakpoints^49^. By also comparing the genome assemblies for Atlantic cod with an assembly for the closely related haddock^50^, we were further able to identify the gadMor2 assembly—representing a migratory Atlantic cod individual—as the carrier of the derived arrangement of the supergenes on LG 1 and 7 but not of that on LG 2. In addition, demographic analyses (Fig. 3l and Supplementary Note 2) indicated that the gadMor2 assembly might also carry the ancestral arrangement on LG 12. The haplotypes with derived arrangements were not consistently characterized by lower genetic diversity than those with ancestral arrangements, contrary to assumptions made in previous studies to distinguish between them^29,30^. As suggested by our demographic analyses (Fig. 3i) and simulations (Supplementary Table 17), this contrast can be explained by an ability of haplotypes with derived arrangements to recover from the initial bottleneck. While this recovery may require substantial frequencies of the derived arrangement (comparable to those of the ancestral one) within the species and sufficient time since the inversion origin to allow the accumulation of new mutations^17^, both of these requirements may be met for the Atlantic cod supergenes.

According to our time-calibrated phylogenomic analyses, the four supergenes in Atlantic cod originated between ~0.40 and ~1.66 Ma. These dates could be underestimates due to gene flux between the haplotypes with derived and ancestral arrangements that could not be accounted for in our age estimation. Nevertheless, we consider these age estimates as evidence that at least some of the four supergenes had separate origins, as the age estimate for the supergene on LG 7 is more than four times that for the supergene on LG 12, and their confidence intervals are clearly non-overlapping. This conclusion is further supported by the homogeneity in sliding-window age estimates from the beginning to the end of each supergene and by the support for demographic bottlenecks coinciding with the inferred age estimates (Figs. 3 and 4). However, a joint origin cannot be excluded for the supergenes on LGs 1, 2 and 12. Our age estimates thus indicate that some but not necessarily all of the four supergenes evolved separately, after the Atlantic cod’s divergence from the walleye pollock and before the divergence of all extant Atlantic cod populations.

Our results strongly support gene flux between the two haplotypes of the supergene on LG 1 (Fig. 3b): between all carriers of the ancestral arrangement and the migratory individuals from Newfoundland (which carry the derived arrangement) and between all carriers of the derived arrangement and the stationary individuals from Newfoundland (which carry the ancestral arrangement). Due to the elevated GC content of sites shared between the haplotypes, we interpret these signals of gene flux as evidence for gene conversion occurring at Newfoundland, but we note that double crossovers could also influence GC content through crossover-associated gene conversion^62^. It remains unclear why signals of gene flux are much weaker at other locations where the two arrangements co-occur (such as in northern Norway).

Our results demonstrated the occurrence of double crossover between the two haplotypes of the LG 12 supergene as a second mechanism allowing gene flux between haplotypes with derived and ancestral arrangements. The sequence introduced through double crossover included the vitellogenin gene cluster^63^, which is assumed to contribute to the proper hydration of fish eggs and thus to the maintenance of neutral buoyancy^58–61^. In contrast to the fully marine environments of the open North Atlantic, the almost land-locked Baltic Sea has a severely reduced salinity and thus requires adaptations in the hydration of eggs, so that they remain neutrally buoyant at a salinity of ~12.5 ppt^64^ (compared with ~35 ppt in the North Atlantic and ~18 ppt at Kiel Bight^65^) and do not sink to anoxic layers^66–68^. The three vitellogenin genes may thus be under selection in Atlantic cod from the Baltic Sea^28^, increasing the frequency of the introduced sequence within the Bornholm Basin population.

The presence of four long (4–17 Mbp) and old (0.40–1.66 Ma) inversion-based supergenes in Atlantic cod adds to recent findings of inversions of similar size and/or age in butterflies^12^, ants^2^, birds^4^, lampreys^69^ and *Drosophila*^56^. For non-model organisms, these findings are largely owed to improvements in sequencing technology within the past decade, including long-read sequencing and chromosome conformation capture techniques, and may become more common as these techniques are applied to an increasing number of species. These findings are, however, in contrast to earlier expectations based on theoretical work and empirical studies on selected model organisms. Only about 20 years ago, the available evidence indicated that inversions (and thus inversion-based supergenes) would be “generally not ancient”^20^ because they would either degenerate through the accumulation of mutation load or erode if gene flux occurred through gene conversion and double crossover^20,55,70^. However, we observed neither mutation-load accumulation nor erosion of supergenes in Atlantic cod. Mutation load was not increased within the four supergenes compared to the genome-wide background. And supergene erosion—at least when resulting from double crossovers—would be expected to produce U-shaped divergence profiles^20,55,56^, but no such profiles were found for the Atlantic cod supergenes (Fig. 4). These observations mirror those made recently by Yan et al.^2^ for a supergene in fire ants, and we thus concur with their conclusion that “low levels of recombination and/or gene conversion may play an underappreciated role in preventing rapid degeneration of supergenes”. But, since our results also indicated that selection may have acted on sequences exchanged between supergene haplotypes, we further suggest that—just like in interbreeding species that maintain stable species boundaries despite frequent hybridization^71^—selective purging of introduced sequences may also be important for the maintenance of supergenes: it can maintain the rate of gene flux between haplotypes at exactly the right balance between too little flux and the consequential mutation-load accumulation, and too much flux and the resulting supergene erosion.

## Methods

### Construction of the gadMor_Stat genome assembly

We performed high-coverage genome sequencing for a stationary Atlantic cod individual (LOF1106Z11) sampled at the Lofoten islands in northern Norway. The specimen was selected on the basis of a preliminary investigation that had suggested that it carried, homozygously on each of the four LGs 1, 2, 7 and 12, a supergene haplotype that was complementary to the one of the gadMor2 genome^31^, which represents a migratory individual from northern Norway. We used the Pacific Biosciences RS II platform, operated by the Norwegian Sequencing Centre (NSC; www.sequencing.uio.no), to generate 2.4 million PacBio SMRT reads with a total volume of 12.5 gigabases (Gbp). This is approximately equivalent to a 19× coverage of the Atlantic cod genome, the size of which has been estimated at 650 Mbp^31^. The PacBio SMRT reads were assembled with Celera Assembler v.8.3rc2 (ref. ^72^), adjusting the following settings according to the nature of the PacBio reads (all others were left at their defaults): merSize, 16; merThreshold, 0; merDistinct, 0.9995; merTotal, 0.995; ovlErrorRate, 0.40; ovlMinLen, 500; utgGraphErrorRate, 0.300; utgGraphErrorLimit, 32.5; utgMergeErrorRate, 0.35; utgMergeErrorLimit, 40; utgBubblePopping, 1; utgErrorRate, 0.40; utgErrorLimit, 25; cgwErrorRate, 0.40; cnsErrorRate, 0.40. The consensus sequence of the assembly was polished with Quiver v.0.9.0 (ref. ^73^) and refined with Illumina reads sequenced for the same individual (see below). A total volume of 6.2 Gbp of Illumina reads were mapped to the assembly with BWA MEM v.0.7.12-r1039 (ref. ^74^) and sorted and indexed with SAMtools v.1.10 (refs. ^75,76^). Subsequently, Pilon v.1.16 (ref. ^77^) was applied to recall consensus, and assembly completeness was assessed with BUSCO v.5.0 (ref. ^47^), using the Actinopterygii dataset of conserved gene sequences.

### Whole-genome sequencing and population-level variant calling

Twenty-two migratory and stationary Atlantic cod individuals were sampled in Canada, Iceland, the United Kingdom, Germany, Sweden and Norway using longline fishing, hand fishing, gillnets and trawling, and were subjected to medium-coverage (8.1–17.0×) whole-genome Illumina sequencing (Fig. 2 and Supplementary Table 4). DNA extraction, library preparation and sequencing were performed at the NSC using the Illumina Truseq DNA PCR-free kit for DNA extraction and an Illumina HiSeq 2500 instrument with V4 chemistry for paired-end (2 × 125 bp) sequencing. The reads from these 22 individuals were mapped to the gadMor2 assembly for Atlantic cod^31^, together with Illumina reads from the two individuals used for the gadMor2 and gadMor_Stat assemblies. Mapping was done with BWA MEM v.0.7.17, followed by sorting and indexing with SAMtools v.1.9. Read duplicates were marked and read groups were added with Picard tools v.2.18.27 (http://broadinstitute.github.io/picard). Variant calling was performed with GATK’s v.4.1.2.0 (refs. ^78,79^) HaplotypeCaller and GenotypeGVCFs tools, followed by indexing with BCFtools v.1.9 (ref. ^76^).

### Delimiting high-LD regions associated with inversions

As chromosomal inversions locally suppress recombination between individuals carrying the inversion and those that do not, we used patterns of LD to guide the delimitation of inversion regions for each of the four supergenes^28,32,41^. To maximize the signal of LD generated by the inversions, we selected 100 Atlantic cod individuals from a separate dataset (Supplementary Table 18) so that for each of the four supergenes, 50 individuals carried two copies of one of the two alternative supergene haplotypes, and the other 50 individuals carried two copies of the other. Variant calls of the 100 individuals were filtered with BCFtools, excluding all indels and multinucleotide polymorphisms and setting all genotypes with a Phred-scaled quality below 20, a read depth below 3 or a read depth above 80 to missing. Sites with more than 80% missing data or a minor allele count below 20 were then removed from the dataset with VCFtools v.0.1.14 (ref. ^80^). Linkage among SNPs spaced less than 250,000 bp from each other was calculated with PLINK v.1.90b3b^81^. The strength of short- to mid-range linkage for each SNP was then quantified as the sum of the distances (in bp) between that SNP and all other SNPs with which it was found to be linked with *R*^2^ > 0.8. We found this measure to illustrate well the sharp decline of linkage at the boundaries of the four supergenes (Fig. 1).

### Contig mapping

To confirm the presence of chromosomal inversions within the four supergenes on LGs 1, 2, 7 and 12 of the Atlantic cod genome, we aligned contigs of the gadMor_Stat assembly to the gadMor2 assembly by using BLASTN v.2.2.29 (ref. ^82^) searches with an *e*-value threshold of 10^−10^, a match reward of 1, and mismatch, gap opening and gap extension penalties of 2, 2 and 1, respectively. Matches were plotted and visually analysed for contigs of the gadMor_Stat assembly that either span the boundaries of the four supergene regions or map partially close to both boundaries of one such region. We considered the latter to support the presence of a chromosomal inversion if one of two parts of a contig mapped just inside one boundary and the other part mapped just outside the other boundary, and if the two parts had opposite orientations; in contrast, an observation of contigs clearly spanning one of the boundaries would reject the assumption of an inversion. To further assess which of the two Atlantic cod genomes (gadMor2 or gadMor_Stat) carries the haplotype with the derived arrangement at each of the four regions, we also aligned contigs of the genome assembly for *Melanogrammus aeglefinus* (melAeg)^50^ to the gadMor2 assembly.

### Three-way whole-genome alignment

To identify the regions that are most reliably orthologous among the gadMor2, gadMor_Stat and melAeg assemblies, we generated whole-genome alignments using three different approaches. First, we visually inspected the plots of BLASTN matches (see above), determined the order and orientation of all gadMor_Stat and melAeg contigs unambiguously mapping to the gadMor2 assembly, and then combined these contigs into a single FASTA file per species and gadMor2 LG. For each LG, pairwise alignments were then produced with the program MASA-CUDAlign v.3.9.1.1024 (ref. ^83^). Second, we used the program LASTZ v.1.0.4 (ref. ^84^) to align both the gadMor_Stat assembly and the melAeg assembly to the gadMor2 assembly, after masking repetitive regions in all three assemblies with RepeatMasker v.1.0.8 (http://www.repeatmasker.org). Third, Illumina sequencing reads of the individuals used for the three assemblies were mapped to the gadMor2 assembly with BWA MEM, followed by sorting and indexing with SAMtools and conversion of the result files to FASTA format. Finally, we generated a conservative three-way whole-genome alignment by comparing the three different types of alignments and setting all sites to missing at which one or more of the three alignment types differed. Alignment sites that opened gaps in the gadMor2 sequence were deleted so that the resulting strict consensus alignment retained the coordinate system of the gadMor2 assembly.

On the basis of the three-way whole-genome alignment, we calculated the sequence divergence between the gadMor_Stat and gadMor2 assemblies, relative to the sequence divergence between the melAeg and gadMor2 assemblies, in sliding windows of 100,000 bp. Sequence divergence was calculated in pairwise sequence comparisons as uncorrected *p*-distances. We also used the three-way whole-genome alignment to generate a mask of unreliable alignment sites, including all sites that had been set to missing in the alignment.

### Estimating divergence times of Gadinae

To estimate the ages of supergene origins in Atlantic cod on the basis of the carefully calibrated timeline of Musilova et al.^85^, we performed two nested phylogenetic analyses. The first one used constraints specified according to the results of Musilova et al.^85^ to estimate the divergence times of species within the subfamily Gadinae. The second analysis was constrained according to the results of the first one to refine the divergence-time estimates among species of the genera *Gadus* (Atlantic cod, walleye pollock, Greenland cod and Pacific cod), *Arctogadus* (Arctic cod; *A*. *glacialis*) and *Boreogadus* (polar cod; *B*. *saida*) with a larger dataset and while co-estimating introgression (see below).

The phylogenomic dataset used for the first phylogenetic analysis comprised genome assemblies for eight Gadinae species published by Malmstrøm et al.^86^; a genome assembly for the most closely related outgroup, *Brosme brosme*^86^; the gadMor2 assembly for Atlantic cod; and sets of unassembled Illumina reads for Pacific cod and Greenland cod^46^ (Supplementary Table 19). Aiming to identify sequences orthologous to 3,061 exon markers used in a recent phylogenomic analysis of teleost relationships by Roth et al.^87^, we first performed targeted assembly of these markers from the sets of Illumina reads for Pacific cod and Greenland cod. Targeted assembly was conducted with Kollector v.1.0.1 (ref. ^88^), using marker sequences of Atlantic cod from Roth et al.^87^ as queries. From the set of whole-genome and targeted assemblies, candidate orthologues to the 3,061 exon markers used by Roth et al.^87^ were then identified through TBLASTN searches, using sequences of *Danio rerio* as queries, as in the earlier study. The identified sequences were aligned with MAFFT and filtered to exclude potentially remaining paralogous sequences and misaligned regions: we removed all sequences with TBLASTN bitscore values below 0.9 times the highest bitscore value and all sequences that had d*N*/d*S* values greater than 0.3 in comparison with the *Danio rerio* queries, we removed codons from the alignment for which BMGE v.1.1 (ref. ^89^) determined a gap rate greater than 0.2 or an entropy-like score greater than 0.5, and we excluded exon alignments with a length shorter than 150 bp, more than two missing sequences or a GC-content standard deviation greater than 0.04. We then grouped exon alignments by gene and excluded all genes that (1) were represented by less than three exons, (2) had one or more completely missing sequences, (3) were supported by a mean RAxML v.8.2.4 (ref. ^90^) bootstrap value lower than 0.65, (4) were located within the four supergene regions, (5) exhibited significant exon tree discordance according to an analysis with Concaterpillar v.1.7.2 (ref. ^91^) or (6) had a gene tree with non-clock-like evolution (a mean estimate for coefficient of variation greater than 0.5 or a 95% HPD interval including 1.0) according to a relaxed-clock analysis with BEAST 2 (ref. ^92^). Finally, concatenated exon alignments per gene were inspected by eye, and six genes were removed due to remaining possible misalignment. The filtered dataset included alignments for 91 genes with a total alignment length of 106,566 bp and a completeness of 92.8%.

We inferred the species tree of Gadinae with StarBEAST2 (refs. ^92,93^) under the multispecies coalescent model, assuming a strict clock, constant population sizes and the birth–death tree model^94^, and averaging over substitution models with the bModelTest package^95^ for BEAST 2. For time calibration, we placed log-normal prior distributions on the age of the divergence of the outgroup *Brosme brosme* from Gadinae (mean in real space, 32.325; standard deviation, 0.10) and on the crown age of Gadinae (mean in real space, 18.1358; standard deviation, 0.28); in both cases, the distribution parameters were chosen to approximate the age estimates for these two divergence events obtained by Musilova et al.^85^. We performed five replicate StarBEAST2 analyses, each with a length of one billion Markov-chain Monte Carlo (MCMC) iterations. After merging replicate posterior distributions, the effective sample sizes (ESSs) for all model parameters were greater than 1,000, indicating full stationarity and convergence of MCMC chains. We then used TreeAnnotator from the BEAST 2 package to summarize the posterior tree distribution in the form of a MCC consensus tree with BPPs as node support^96^.

### Estimating divergence times and introgression among species of the genera *Gadus*, *Arctogadus* and *Boreogadus*

To further investigate divergence times and introgression among species of the closely related genera *Gadus*, *Arctogadus* and *Boreogadus*, we used a second phylogenomic dataset based on read mapping to the gadMor2 assembly. This dataset included Illumina read data for all four species of the genus *Gadus*^46,86^, Arctic cod^86^ and polar cod^86^, as well as *Merlangius merlangius*, *Melanogrammus aeglefinus* and *Pollachius virens*^50,86^, which we here considered outgroups (Supplementary Table 20). Read data from a stationary individual and a migratory individual (both sampled at the Lofoten islands) were used to represent Atlantic cod. Mapping, read sorting and indexing were again performed with BWA MEM and SAMtools, and variant calling was again performed with GATK’s HaplotypeCaller and GenotypeGVCFs tools as described above, except that we now also exported invariant sites to the output file. To limit the dataset to the most reliably mapping genomic regions, we applied the mask of unreliable sites generated from the three-way whole-genome alignment (see above), resulting in a set of 19,035,318 SNPs. We then extracted alignments from GATK’s output files for each non-overlapping window of 5,000 bp for which no more than 4,000 sites were masked, setting all genotypes with a Phred-scaled likelihood below 20 to missing. Alignments were not extracted from the four supergene regions, and windows with less than 100 variable sites were ignored. As we did not model recombination within alignments in our phylogenomic inference, the most suitable alignments for the inference were those with weak signals of recombination. We therefore calculated the number of hemiplasies per alignment by comparing the number of variable sites with the parsimony score, estimated with PAUP*^97^, and excluded all alignments that had more than ten hemiplasies. Finally, we again removed all alignment sites for which BMGE determined a gap rate greater than 0.2 or an entropy-like score greater than 0.5. The resulting filtered dataset was composed of 109 alignments with a total length of 383,727 bp and a completeness of 91.0%.

We estimated the species tree and introgression among *Gadus*, *Arctogadus* and *Boreogadus* under the isolation-with-migration model implemented in the AIM package^98^ for BEAST 2. The inference assumed a strict clock, constant population sizes, the pure-birth tree model^99^ and the HKY^100^ substitution model with gamma-distributed rate variation among sites^101^. We time-calibrated the species tree with a single log-normal prior distribution on the divergence of *Pollachius virens* from all other taxa of the dataset (mean in real space, 8.56; standard deviation, 0.08), constraining the age of this divergence event according to the results of the analysis of divergence times of Gadinae (see above; Supplementary Fig. 5). We performed ten replicate analyses that each had a length of five billion MCMC iterations, resulting in ESS values greater than 400 for all model parameters. The posterior tree distribution was subdivided according to tree topology and inferred gene flow, and we produced separate MCC consensus trees for each of the tree subsets.

To further test for introgression among *Gadus*, *Arctogadus* and *Boreogadus*, we calculated Patterson’s *D*-statistic from the masked dataset for all possible species trios (with *Pollachius virens* fixed as the outgroup) using the Dtrios function of Dsuite v.0.1.r3 (ref. ^102^). For the calculation of the *D*-statistic, species trios were sorted in two ways: with a topology fixed according to the species tree inferred under the isolation-with-migration model (*D*_fix_), and so that the number of BBAA patterns was greater than those of ABBA and BABA patterns (*D*_BBAA_). The significance of the statistic was assessed through block-jackknifing with 20 blocks of equal size. For the trios with the most significant signals of introgression, we further used the Dinvestigate function of Dsuite to calculate the introgression proportion (*f*_dM_-statistic)^103^ within sliding windows of 50 SNPs, overlapping by 25 SNPs.

To corroborate the introgression patterns inferred with Dsuite, we performed two analyses based on comparisons of the frequencies of trio topologies in maximum-likelihood phylogenies. Alignments for these analyses were selected as for the species-tree inference under the isolation-with-migration model, except that up to 20 hemiplasies were allowed per alignment. The resulting set of 851 alignments had a total length of 3,052,697 bp and a completeness of 91.0%. From each of these alignments, a maximum-likelihood phylogeny was inferred with IQ-TREE v.1.6.8 (ref. ^104^) with a substitution model selected through IQ-TREE’s standard model selection. Branches with lengths below 0.001 were collapsed into polytomies. On the basis of the inferred maximum-likelihood trees, we calculated, for all possible species trios, the *D*_tree_-statistic of Ronco et al.^105^, a tree-based equivalent to Patterson’s *D*-statistic in which the frequencies of pairs of sister taxa are counted in a set of trees instead of the frequencies of shared sites in a genome (a related measure was proposed by Huson et al.^106^): *D*_tree_ = (*f*_2nd_ − *f*_3rd_)/(*f*_2nd_ + *f*_3rd_), where for a given trio, *f*_2nd_ is the frequency of the second-most-frequent pair of sisters, and *f*_3rd_ is the frequency of the third-most-frequent (thus, the least frequent) pair of sisters. We applied genealogy interrogation^107^ as a second tree-based analysis of introgression, comparing the likelihoods of trees with alternative topological constraints for the same alignment, as in Barth et al.^71^. We tested two hypotheses of introgression with this method: (1) introgression between Arctic cod and either polar cod or the group of the four species of the genus *Gadus*, and (2) introgression between Greenland cod and the two sister species walleye pollock and Atlantic cod.

### Estimating divergence times, demography and gene flow among Atlantic cod populations

To investigate divergence times among Atlantic cod populations, we applied phylogenetic analyses to the dataset based on whole-genome sequencing and variant calling for 24 Atlantic cod individuals (Supplementary Table 4). This dataset included, now considered as outgroups, the same representatives of walleye pollock, Pacific cod, Greenland cod, Arctic cod and polar cod as our analyses of divergence times and introgression among *Gadus*, *Arctogadus* and *Boreogadus* (see above). ‘Migratory’ and ‘stationary’ Atlantic cod individuals from Newfoundland, Iceland, Lofoten and Møre were used as separate groups in these analyses. Subsequent to mapping with BWA MEM and variant calling with GATK’s HaplotypeCaller and GenotypeGVCFs tools, we filtered the called variants with BCFtools to include only sites for which the Phred-scaled *P* value for Fisher’s exact test was smaller than 20, the quality score normalized by read depth was greater than 2, the root-mean-square mapping quality was greater than 20, the overall read depth across all individuals was between the 10% and 90% quantiles, and the inbreeding coefficient was greater than −0.5. We further excluded sites if their Mann–Whitney–Wilcoxon rank-sum test statistic was smaller than −0.5 either for site position bias within reads or for mapping quality bias between reference and alternative alleles. After indels were normalized with BCFtools, SNPs in proximity to indels were discarded with a filter that took into account the length of the indel: SNPs were removed within 10 bp of indels that were 5 bp or longer, but only within 5, 3 or 2 bp if the indel was 3–4, 2 or 1 bp long, respectively. After we applied this filter, all indels were removed from the dataset. For the remaining SNPs, genotypes with a read depth below 4 or a genotype quality below 20 were set to missing. Finally, we excluded all sites that were no longer variable or had more than two different alleles; the filtered dataset then contained 20,402,423 biallelic SNPs.

We inferred the divergence times among Atlantic cod populations from the SNP data under the multispecies coalescent model with the SNAPP add-on package for BEAST 2 (refs. ^108,109^). Due to the high computational demand of SNAPP, we performed this analysis only with a further reduced set of 1,000 SNPs, randomly selected from all biallelic SNPs that were without missing genotypes and located outside of the supergene regions. The input files for SNAPP were prepared with the script snapp_prep.rb^109^, implementing a strict-clock model and a pure-birth tree model. The tree of Atlantic cod populations and outgroup species was time-calibrated with a single log-normal prior distribution (mean in real space, 3.83; standard deviation, 0.093) that constrained the root age of the tree according to the results of the analysis of divergence times and introgression among *Gadus*, *Arctogadus* and *Boreogadus* (see above; Extended Data Fig. 2b and Supplementary Fig. 6). We performed three replicate SNAPP analyses, each with a length of 400,000 MCMC iterations, resulting in ESS values that were all greater than 400. The posterior tree distribution was again summarized as a MCC consensus tree.

Gene flow among Atlantic cod populations and outgroup species was investigated with Dsuite from all biallelic SNPs that were without missing genotypes and located outside of the four supergene regions; there were 408,574 of these. The gene flow analyses were performed with Dsuite’s Dtrios function as described above.

Population sizes over time were estimated for all sampled Atlantic cod populations with Relate v.1.1.2 (ref. ^110^). To maximize the number of suitable SNPs for this analysis, we excluded all outgroups except the sister species (walleye pollock) and repeated variant calling and filtering with the same settings as before. After we applied a mask to exclude all variants from repetitive regions in the gadMor2 assembly (784,488 bp in total)^31^, 10,872,496 biallelic SNPs remained and were phased with BEAGLE v.5.1 (ref. ^111^), with the population size assumed by BEAGLE set to 10,000. We excluded all sites that were heterozygous in the walleye pollock individual and then reconstructed an ‘ancestral’ genome sequence from the gadMor2 assembly and the called variants for the walleye pollock. Following this reconstruction, we removed the walleye pollock from the set of SNPs and excluded all sites that had become monomorphic after this removal, leaving 7,101,144 SNPs that were biallelic among the sampled Atlantic cod individuals. In addition to the ‘ancestral’ genome sequence and the set of biallelic SNPs, we prepared a mask for the Relate analysis, covering all sites that were also included in the mask for repetitive regions, all sites that would have been excluded from variant calling due to proximity to indels (see above) and all sites that were ignored in the reconstruction of the ‘ancestral’ sequence due to heterozygous genotype calls for the walleye pollock individual.

As Relate further requires an estimate of the mutation rate, we calculated this rate for the filtered set of SNPs as the mean number of substitutions between Atlantic cod individuals from the Northwest Atlantic (that is, from the populations Newfoundland and Labrador) and those from the Northeast Atlantic (that is, from all other populations), divided by two times the expected coalescence time between the two groups and the genome size. We excluded the four LGs carrying supergenes from this calculation. The expected coalescence time was calculated as the divergence time between the two groups, which was estimated in the analysis with SNAPP as 65,400 years (Fig. 2), plus the expected time to coalescence within the common ancestor, which is the product of the generation time and the diploid population size under the assumption of a panmictic ancestral population. With an assumed generation time of 10 years^112^ and a population size of 57,400, as estimated in the SNAPP analysis, the expected time to coalescence within the common ancestor is 574,000 years, and the total expected coalescence time was thus set to 65,400 + 574,000 = 639,400 years. As the mean number of substitutions between the individuals of the two groups was 878,704.31 and the size of the gadMor2 assembly without LGs 1, 2, 7 and 12, and excluding masked sites, is 419,183,531 bp, the calculated mutation rate was *μ* = 878,704.31/(2 × 639,400 × 419,183,531) = 1.64 × 10^−9^ per bp per year, or 1.64 × 10^−8^ per bp per generation. Because the number of substitutions was calculated from the filtered set of SNPs, this rate is likely to underestimate the true mutation rate of Atlantic cod; however, because the same filtered set of SNPs was used as input for Relate, this rate is applicable in our inference of population sizes over time. The input file was converted from variant call format to haplotype format using RelateFileFormats with the flag “--mode ConvertFromVcf”. The script PrepareInputFiles.sh was used to flip genotypes according to the reconstructed ‘ancestral’ genome sequence and to adjust distances between SNPs using the mask prepared for this analysis. Relate was first run to infer genome-wide genealogies and mutations assuming the above calculated mutation rate of 1.64 × 10^−8^ per bp per generation and a diploid effective population size of 50,000. This was followed by an estimation of population-size changes over time by running the script EstimatePopulationSize.sh for five iterations, applying the same mutation rate and setting the threshold to remove uninformative trees to 0.5. The tools and scripts RelateFileFormats, PrepareInputFiles.sh and EstimatePopulationSize.sh are all distributed with Relate.

### Estimating divergence times, demography and gene flux specific to supergenes

The analyses of divergence times, demography and gene flux among Atlantic cod populations were repeated separately with SNPs from each of the four supergene regions on LGs 1, 2, 7 and 12. While the SNAPP analyses for these regions were again performed with reduced subsets of 1,000 SNPs per region, the data subsets used in analyses of gene flux with Dsuite comprised 11,474, 3,123, 10,412 and 10,339 biallelic SNPs, and those used in the analyses of demography with Relate comprised 211,057, 71,046, 130,918 and 130,620 biallelic SNPs, respectively. The mutation rate used as input for these Relate analyses was identical to the one used for the analysis with genome-wide SNPs.

### Estimating population divergence times across the genome

In addition to the genome-wide and supergene-specific SNAPP analyses that used biallelic SNPs from the entire genome or the entire length of supergene regions, we performed sliding-window SNAPP analyses across all LGs to quantify differences in population divergence times across the genome. Our motivation for these analyses was primarily to assess whether divergence times were homogeneous over the lengths of supergenes, as differences in these divergence times within supergenes could be informative both about the presence of separate inversion within these regions and about their erosion processes. Additionally, we expected that these analyses could reveal further putative inversions elsewhere in the genome if they existed.

From the set of 20,402,423 biallelic SNPs, we extracted subsets of SNPs for each non-overlapping window of a length of 250,000 bp, with a minimum distance between SNPs of 50 bp. We discarded windows with less than 500 remaining biallelic SNPs and used a maximum of 1,000 biallelic SNPs per window; these were selected at random if more biallelic SNPs were available. Input files for SNAPP were then prepared as for the genome-wide and supergene-specific SNAPP analyses. Per window, we performed two replicate SNAPP analyses with an initial length of 100,000 MCMC iterations, and these analyses were resumed up to a maximum of 500,000 MCMC iterations as long as the lowest ESS value was below 100. Windows with less than 300 sufficiently complete SNPs for SNAPP analyses, with an ESS value below 100 after the maximum number of MCMC iterations or with a mean BPP node support value below 0.5 were discarded after the analysis. Per remaining window, posterior tree distributions from the two replicate analyses were combined and summarized in the form of MCC consensus trees. Additionally, a random sample of 100 trees was drawn from each combined posterior distribution.

Instead of showing all resulting trees, we developed a type of plot that shows, without loss of phylogenetic information, the divergence times stacked on each other on a single axis, which allowed us to illustrate these divergence times efficiently across LGs. For this plot, all trees were first ladderized, outgroups were pruned and the divergence times between each pair of populations adjacent to each other on the ladderized trees were extracted. Per window, the order of populations on the ladderized tree, together with the extracted divergence times between them, was used to define the positions of points on the vertical axis of the plot, so that each point represents a population, and their vertical distances indicate the divergence times between populations that are next to each other on the ladderized tree. The positions of windows on the LGs were used to place these dots on the horizontal axis of the plot, and all dots representing the same population were connected by lines to produce the complete plot of divergence times across LGs.

### Reporting Summary

Further information on research design is available in the Nature Research Reporting Summary linked to this article.

## Supplementary information

Supplementary Information Supplementary Notes 1–4, Figs. 1–9 and Tables 1–23.

Reporting Summary

## Data Availability

The gadMor_Stat assembly (ENA accession number GCA_905250895) and read data for all Atlantic cod specimens listed in Supplementary Table 4 are deposited on ENA with project number PRJEB43149. The alignment files, SNP datasets in PED and VCF format, and input and output of the phylogenetic analyses are available from Zenodo (10.5281/zenodo.4560275). Source data are provided with this paper.

## Source data

Source Data Fig. 1 Genetic distance and linkage per SNP.
Source Data Fig. 2 Maximum-credibility population tree, *D*_BBAA_, *D*_fix_ and demography.
Source Data Fig. 3 Maximum-credibility population tree, *D*_BBAA_, *D*_fix_ and demography for LGs 1, 2, 7 and 12.
Source Data Fig. 4 Population trees for LGs 1, 2, 7 and 12.
Source Data Fig. 5 Alleles at fixed sites.
Source Data Extended Data Fig. 1 Repeat content and mutation load.
Source Data Extended Data Fig. 2 Species trees, *D*_BBAA_, *D*_fix_ and *f*_dM_.
Source Data Extended Data Fig. 3 *F*_ST_ and *d*_XY_ for gadMor2 and gadMor3.

## Code availability

The code for the computational analyses is available from GitHub (http://github.com/mmatschiner/supergenes).
