# Supplementary material for: Supergene origin and maintenance in Atlantic cod
Source: Nat Ecol Evol. 2022 Feb 17;6(4):469–81. doi: 10.1038/s41559-022-01661-x (PMC8986531; doi:10.1038/s41559-022-01661-x)
Supplement: Supplementary file 1 — Supplementary Notes 1–4, Figs. 1–9 and Tables 1–23. [file 41559_2022_1661_MOESM1_ESM.pdf]

nature portfolio
https://doi.org/10.1038/s41559-022-01661-x

Supplementary information

Supergene origin and maintenance in Atlantic cod

In the format provided by the authors and unedited

Supplementary Information

Supergene origin and maintenance in Atlantic cod

Matschiner et al.

Supplementary Notes

Supplementary Note 1: Unreliable support for introgression in the study by Árnason and Halldórsdóttir (2019).

In their study, Árnason and Halldórsdóttir[1] investigated signals of introgression among species of the genera *Gadus*, *Arctogadus*, and *Boreogadus* using a combination of low-coverage ($3\times$) and high-coverage ($12-45\times$) whole-genome-sequencing data. The authors reported discordance between mitochondrial and nuclear phylogenies, elevated D-statistics, and reticulations inferred with PhyloNetworks[2], and interpreted their results as support for i) introgression between *Gadus morhua* and *Gadus ogac*, ii) strong genetic divergence between eastern and western populations of *Arctogadus glacialis*, iii) the (past) existence of ghost lineages as pathways for introgression, iv) introgression between *Gadus morhua* and *Gadus chalcogrammus* or a homoploid hybrid speciation origin of the latter. As we argue below, these conclusions are at least partially unreliable due to mislabelled sequences, reference bias, and incorrect application of statistical tests.

Mislabelled sequences and reference bias generated tree discordance. Árnason and Halldórsdóttir inferred phylogenies using the Neighbor-Joining algorithm[3], implemented in ape[4], from both mitochondrial sequence alignments and nuclear single-nucleotide-polymorphism (SNP) data (Fig. 2 in their study). The resulting trees were discordant with each other because the placement of *Arctogadus glacialis* differed between them. Árnason and Halldórsdóttir interpreted this discordance as evidence for introgression into *Arctogadus glacialis*, the (past) existence of ghost lineages, and — because a second added mitochondrial sequence for *Arctogadus glacialis* did not cluster with the first — strong divergence between separate populations of *Arctogadus glacialis.* Notably, however, both the mitochondrial and the nuclear phylogenies inferred by Árnason and Halldórsdóttir also differed from the consensus view of the relationships among the investigated species, which has been supported rather unambiguously, both by mitochondrial and nuclear datasets, and in a range of publications[5–9]. According to that consensus view, *Gadus morhua* is the sister species to *Gadus chalcogrammus*, *Gadus macrocephalus* is the sister species to *Gadus ogac*, the two pairs of sister species are closer to each other than either is to *Arctogadus glacialis* or *Boreogadus saida*, and the clade formed by all of these six species is monophyletic. In the mitochondrial tree inferred by Árnason and Halldórsdóttir, on the other hand, the sampled *Arctogadus glacialis* specimen clustered with an outgroup, *Micromesistius poutassou*, whereas the same specimen clustered with *Gadus morhua* in their nuclear tree.

As the *Arctogadus glacialis* specimen used by Árnason and Halldórsdóttir was the one specimen sequenced to a far lower read depth ($3\times$) than all other specimens ($12-45\times$) used in their study, and as they had mapped all reads towards the gadMor2[10] genome assembly for *Gadus morhua*, we assumed that reference bias could have led to a placement of *Arctogadus glacialis* near *Gadus morhua* in their nuclear phylogeny, which would explain at least the discordance between their nuclear phylogeny and the consensus view[11, 12]. To test this assumption, we reanalyzed the genomic read data produced by Árnason and Halldórsdóttir together with data from two other recent genomic studies of codfishes (family Gadidae)[8, 13]. We downloaded all accessions used by Árnason and Halldórsdóttir

from the European Nucleotide Archive (ENA): SRR2906188 (*Arctogadus glacialis*), SRR2906185 (*Boreogadus saida*), SRR2906193 (*Gadus ogac*), SRR2906345 (*Gadus macrocephalus*), SRR2906339 (*G. chalcogrammus*), and SRR2906208, SRR2906256, SRR2906368, SRR2906362, SRR2906197, and SRR2906248 (all *G. morhua*). In addition, we downloaded the following accessions produced by Malmstrøm et al.[8]: ERR1473878 (*Pollachius virens*), ERR1473879 (*Melanogrammus aeglefinus*), ERR1473880 and ERR1473881 (*Merlangius merlangus*), ERR1473882 and ERR1473883 (*Arctogadus glacialis*), ERR1473884 and ERR1473885 (*Boreogadus saida*), ERR1473886 (*Gadus chalcogrammus*), ERR1473887, ERR1473888, ERR1473889, and ERR1473890 (all *Gadus morhua*), ERR1473875 (*Brosme brosme*), ERR1473876 (*Trisopterus minutus*), and ERR1473877 (*Gadiculus argenteus*). Finally, we added accession ERR1278928 (*Gadus ogac*) generated by Kirubakaran et al.[13]. To exlude reference bias in our own inference of the nuclear phylogeny, we applied local assembly with Kollector v.1.0.1[14] for a set of nuclear marker sequences from Roth et al.[15], instead of mapping the reads to the gadMor2 assembly. We also identified, aligned, and filtered orthologous sequences as in Roth et al.[15], and concatenated the resulting 210 alignments with a total length of 41,811 bp. From this concatenated alignment, we inferred the nuclear phylogeny with IQ-TREE v.1.6.8[16].

The inferred nuclear phylogeny confirmed our assumption of reference bias: The *Arctogadus glacialis* specimen from Árnason and Halldórsdóttir did not appear close to *Gadus morhua* as in their nuclear phylogeny. Instead, the two included *Gadus chalcogrammus* specimens appeared as the sister clade to a monophyletic group formed by all included *Gadus morhua* specimens and all other relationships also agreed with the consensus view, including the position of the *Arctogadus glacialis* specimen used by Malmstrøm et al., which appeared as the sister to the *Boreogadus saida* specimens. The one exception to this was the specimen identified as *Arctogadus glacialis* by Árnason and Halldórsdóttir[1] (ENA accession SRR2906188): Instead of clustering with the *Arctogadus glacialis* from Malmstrøm et al., this specimen appeared as the sister to the *Trisopterus minutus* specimen (accession ERR1473876). A reanalysis of mitochondrial sequences, extracted from the same set of accessions, produced the exact same topology, in which the *Arctogadus glacialis* specimen from Árnason and Halldórsdóttir once again appeared as the sister of the *Trisopterus minutus* specimen. As the genus *Trisopterus* is likely the sister lineage to the genus *Micromesistius*[17] (which was not included in our reanalysis), this result is entirely consistent with the position inferred by Árnason and Halldórsdóttir based on mitochondrial data, under the assumption that the specimen considered by them to be an *Arctogadus glacialis* specimen is mislabelled and in fact represents the species *Micromesistius poutassou*. This assumption of taxon mislabeling appears even more plausible as the mitochondrial sequence of the "*Arctogadus glacialis*" specimen is 99% identical to the mitochondrial sequence of *Micromesistius poutassou* that Árnason and Halldórsdóttir had obtained from GenBank (accession FR751401) and the two species *Arctogadus glacialis* and *Micromesistius poutassou* are phenotypically similar, grow to similar lengths, and occur both in the region sampled by Árnason and Halldórsdóttir. Thus, we conclude that instead of introgression, ghost lineages, and population separation in *Arctogadus glacialis*, it was reference bias in the low-coverage nuclear data, and mislabeling of a *Micromesistius poutassou* specimen as "*Arctogadus glacialis*" that caused the discordance between the mitochondrial and the nuclear trees inferred by Árnason and

Halldórsdóttir, as well as the discordance between these trees and the consensus view of relations among codfishes.

Incorrect application led to exaggerated D-statistics. To further investigate the occurrence of introgression among codfishes, Árnason and Halldórsdóttir applied Patterson's D-statistic[18, 19] to their nuclear SNP dataset, using ANGSD[20]. A D-statistic significantly different from zero supports introgression between two taxa in a quartet of taxa P1, P2, P3, and P4, if the four taxa are labelled so that P1 and P2 are sister taxa and P4 is the outgroup to the other three. In the study by Árnason and Halldórsdóttir, this basic requirement for the application of D-statistics was not met for 26 out of 30 tested quartets (Fig. 3 of their study), in part because of the use of the specimen mislabelled as "*Arctogadus glacialis*". All of these 26 quartets had produced highly significant D-statistics, but because the test requirement was not met, none of these significant D-statistics actually support introgression. Of the remaining four tested quartets, one produced an insignificant D-statistics and the last three seemed to provide weak support ($0.04 \leq D \leq 0.06$) for introgression between the two Pacific species *Gadus chalcogrammus* and *Gadus macrocephalus*, and between the two Atlantic species *Gadus morhua* and *Gadus ogac*. These inferred occurrences of introgression may be plausible given the overlapping distributions and close relationships of the species involved.

Reference bias and incorrect root placement affected results of PhyloNetworks analyses. Finally, Árnason and Halldórsdóttir used the SNaQ method implemented in PhyloNetworks[2] to infer trees in which reticulation edges were supposed to indicate introgression events (Figs. 4–6 and Supplementary Figures S2 and S3 in their study). This analysis was applied separately to SNP data from each of the 23 linkage groups of the gadMor2 assembly. The results of these analyses are difficult to interpret, as — contrary to expectations — not just the inferred reticulation edges but also the inferred species tree differed strongly among the linkage groups. A common feature to most of the presented trees was a reticulation edge connecting the mislabelled "*Arctogadus glacialis*" specimen, for which only low-coverage (3×) data had been available, with *Gadus morhua* specimens, indicating that, just like the comparison of mitochondrial and nuclear phylogenies, the SNaQ analyses were affected by reference bias in this particular specimen. Additionally, the presented trees suffer from incorrect root placement, given that the most divergent species of the dataset was not *Boreogadus saida*, but *Micromesistius poutassou*, represented by the specimen mislabelled as "*Arctogadus glacialis*".

Supplementary Note 2: Demographic analyses recover signatures of bottlenecks following inversions.

It is likely that each of the inversions associated with the four supergenes in *Gadus morhua* originated just once and that all of the current carriers of the inversion descended from the individual in which it originated. The supergene origin should therefore have been equivalent to an extreme bottleneck event during which the population size was reduced to a single sequence, but which affected only the inversion region, and only the carriers of the derived arrangement. We might thus expect to see signatures of this extreme bottleneck event in analyses of population size over time, in the

form of differences in ancestral population sizes between the haplotypes with derived and ancestral arrangements that date to the time of supergene origin.

To verify that signatures of extreme bottlenecks are detectable in descending genomes even after long periods of time, we first performed a series of coalescent simulations of a panmictic population that experienced a bottleneck during which the population size was reduced to a single sequence, using msprime v.0.7.4[21]. These simulations confirmed that, depending on the age of the bottleneck and the population size following the bottleneck, substantial proportions of the genome coalesce inside the bottleneck and are thus able to carry its signature: For example, 22% of the genome coalesce inside a bottleneck that occurred 300,000 years ago if the population size outside of the bottleneck is 10,000, and 19% of the genome coalesce inside of a million-year old bottleneck if the population size outside of it is 30,000 (Supplementary Table 17). To further verify that such types of bottleneck signatures can be detected with Relate[22] from datasets comparable to our empirical ones, we performed additional simulations with msprime in which we applied a model that mimicked diversification of *Gadus morhua* populations as inferred from the supergene region on LG 2; this model included a bottleneck that affected the ancestor of one of two groups immediately after the divergence between them, and a population size reduction to a single sequence during this bottleneck. Demographic analysis of the simulated data with Relate v.1.1.2 in fact recovered the signature of the bottleneck, in the form of a slight but clear reduction in the estimated population size of members of the affected group that coincided with the age of the bottleneck (Supplementary Figure 8a). The ability of Relate to recover the bottleneck was not affected by an erroneous assumption of homogeneous recombination when data were simulated with a high-resolution recombination map (Supplementary Figure 8b).

After confirming that the program Relate is in principle able to pick up such signals, we tested for the presence of bottleneck signatures associated with inversion events by performing demographic analyses with Relate separately for sets of SNPs from each of the four supergene regions. As expected due to the smaller amount of input data (1–3% compared to the genome-wide SNP data), these analyses produced estimates that were less clear (Fig. 3c,f,i,l) than those obtained with genome-wide SNP data (Fig. 2d). Nevertheless, the supergene-specific demographic analyses supported a difference between the population sizes of haplotypes with derived and ancestral arrangements, with a temporary reduction of population sizes for the haplotype with the derived arrangement that coincided with the estimated ages of the supergenes on LGs 2 and 7 (Fig. 3f,i). For the supergenes on LGs 1 and 12, the difference in the population sizes did not coincide with the supergene ages. However, for the supergene on LG 1, the population size of the haplotype with the derived arrangement was reduced compared to the ancestral arrangement in several time intervals following the supergene origin (Fig. 3c). While our contig-mapping approach had not allowed us to infer which of the two haplotypes of the LG 12 supergene had the derived arrangement, the reduced inferred population size of the haplotype carried by the sampled individuals from the Suffolk, Kiel Bight, and stationary Lofoten populations, in a time interval following the supergene origin, suggested that this haplotype might have the derived arrangement on that linkage group (Fig. 3l). The ancestral population sizes estimated from the supergene regions on LGs 2 and 7 further highlighted that estimates of the current genetic diversity may not always be useful for the

identification of derived arrangements, as the population-size estimates for the haplotype with the ancestral arrangement were in both cases higher than for the derived arrangement at the time of supergene origin, but lower for most of the subsequent time towards the present (Fig. 3f,i).

Supplementary Note 3: Inference of species relationships and introgression among codfishes. To establish the phylogenetic context within which the inversions arose in *Gadus morhua* and to test whether introgression played a role in the origin of the four supergenes[23, 24], we performed Bayesian analysis with the isolation-with-migration model as implemented in the AIM add-on package for BEAST 2[25, 26]. The dataset used in this analysis consisted of 109 alignments of sequences for the four species of the genus *Gadus* (*G. morhua*, *G. chalcogrammus*, *G. ogac*, and *G. macrocephalus*), their most closely related relatives *Arctogadus glacialis* and *Boreogadus saida*, and three outgroups (Extended Data Fig. 2a; Supplementary Table 20). The 109 alignments (total length: 383,727 bp) were sampled from across the genome, excluding the four supergene regions and selecting the regions with the most reliable orthology (i.e., those unambiguouly aligned in the three-way whole-genome alignment) and the weakest signals of recombination (i.e., those with low numbers of hemiplasies). We found strong support (Bayesian posterior probability, BPP: 0.997) for a topology in which *Arctogadus* is either placed as the sister taxon to *Gadus* (BPP: 0.763) or to *Boreogadus* (BPP: 0.234). Introgression was supported (by Bayes factors greater than 10) between *Arctogadus* and *Boreogadus* in both cases and additionally from the common ancestor of the genus *Gadus* to *Arctogadus* in the latter case (Extended Data Fig. 2b, Supplementary Figure 6). Regardless of the position of *Arctogadus*, our analysis with the isolation-with-migration model supported an age of around 4 Ma for the clade comprising the three genera (mean estimates: 3.81 Ma and 3.99 Ma; 95% HPD: 4.44–3.19 Ma and 4.56–3.33 Ma; Extended Data Fig. 2b).

Introgression among the three genera was further supported by Patterson's D-statistic[18, 19]. Using Dsuite[27], we calculated for all possible species trios two versions of this statistic from a set of 19,035,318 SNPs: D_{fix}, for which taxa in the trio are arranged according to a provided input tree, and D_{BBAA}, for which taxa are arranged so that the number of sites with the "BBAA" pattern is maximised. The strongest signals of introgression were found once again between *Arctogadus* and *Boreogadus*, in a trio together with *Gadus morhua* ($D_{\text{fix}} = 0.360$, $p < 10^{-10}$; Extended Data Fig. 2c, Supplementary Tables 22 and 23). Within the genus *Gadus*, the D-statistic additionally provided strong support for introgression between the geographically co-occurring *G. ogac* and *G. morhua* in a trio with *G. macrocephalus* ($D_{\text{fix}} = D_{\text{BBAA}} = 0.283$, $p < 10^{-10}$; Supplementary Tables 22 and 23). In both cases, signals of introgression quantified with the f_{dM}-statistic[28] (an alternative to the D-statistic that is unbiased in small genomic regions[29] and symmetrically distributed around zero in the absence of introgression[27]) were found on all chromosomes, and were not affected by the choice of genome representing *Gadus morhua* (Extended Data Fig. 2d). The introgression signal was not elevated in supergene regions, suggesting that they did not arise in *G. morhua* due to introgression from *G. ogac*. The occurrence of introgression between *Boreogadus* and *Arctogadus* was corroborated by a tree-based equivalent to Patterson's D-statistic that does not rely on the molecular-clock

assumption (the D_{tree}-statistic of Ronco et al.[30]) and by genealogy interrogation[31, 32], but introgression between *G. ogac* and *G. morhua* did not receive this additional support (Supplementary Figure 9).

Supplementary Note 4: Interpretation of signals of introgression between *Gadus morhua* and *Gadus ogac*.

We observed strong signals of introgression between *Gadus morhua* and *Gadus ogac*, affecting all sampled populations of *Gadus morhua* (Figs. 2c, 3b,e,h,k and Extended Data Fig. 2c). Given that these signals were based on datasets mapped to the gadMor2 assembly, reference bias could potentially explain the observed introgression signals if the sequence data for *Gadus ogac* would have lower quality or shorter read lengths than that for *Gadus macrocephalus*[11], the sister species of *Gadus ogac*. This, however, appears unlikely, as the samples of both species were sequenced in the same study[1], with the same protocol, and to a similar read depth (17.8–27.5×). Nevertheless, to rule out reference bias as an explanation of these introgression signals, we repeated tests for introgression with an alternative dataset that was mapped to melAeg sequences from the threeway whole-genome alignment, representing *Melanogrammus aeglefinus*, a common outgroup to all taxa included in the dataset. Just like the original dataset mapped to gadMor2, this alternative dataset mapped to melAeg supported introgression between all *Gadus morhua* populations and *Gadus ogac* with $0.063 \leq D \leq 0.084$ ($p < 10^{-4}$). The absence of reference bias was further supported by a comparison of the numbers of reads with reference and alternate alleles at sites called as heterozygous in *Gadus ogac*, showing that sites with more reads carrying the reference allele are not more frequent than those with more reads carrying the alternate allele ($p > 0.05$), regardless of whether the dataset was mapped to gadMor2 or melAeg.

On the other hand, the fact that the introgression signals with *Gadus ogac* are largely homogenous across all sampled *Gadus morhua* populations, and with both the ancestral and derived arragments of the four supergenes, appears puzzling: Any introgression from *Gadus ogac* could only have occurred after its divergence from *Gadus macrocephalus* (~40 ka), but because that divergence time is far younger than that of Northwest and Northeast Atlantic populations of *Gadus morhua* (~65 ka) or those between the supergene haplotypes (0.40–1.66 Ma), multiple separate but similarly strong introgression events would be required to explain the homogeneous signal. The signals of introgression with *Gadus ogac* are therefore best explained by introgression in the opposite direction — from *Gadus morhua* into *Gadus ogac*. In this case, introgression from one *Gadus morhua* population that co-occurs with *Gadus ogac* in the Northwest Atlantic would be sufficient to explain the observed signals for introgression, because it would increase allele sharing with *Gadus ogac* more or less evenly among all *Gadus morhua* populations, due to the relatedness among the *Gadus morhua* populations and supergene haplotypes. Under this assumption of gene flow from a Northwest Atlantic population into *Gadus ogac*, we expect a decreasing homogeneity of the introgression signal when it is quantified for more divergent supergene haplotypes, and this expectation is in

fact met as the signal appears the least homogeneous for the supergene on LG 7 — the oldest of the supergenes — where it is markedly stronger for the haplotype that is carried by the Northwest Atlantic populations (Fig. 3h). The conclusion that *Gadus ogac* genomes carry introgression from *Gadus morhua* is also consistent with recent studies suggesting that introgression is more efficiently purged in species with larger population sizes[33–35], given that *Gadus ogac* appears to have low genetic diversity compared to *Gadus morhua*.

Supplementary Figures

Supplementary Figure 1: Concordance between reference genomes for *Gadus morhua*.

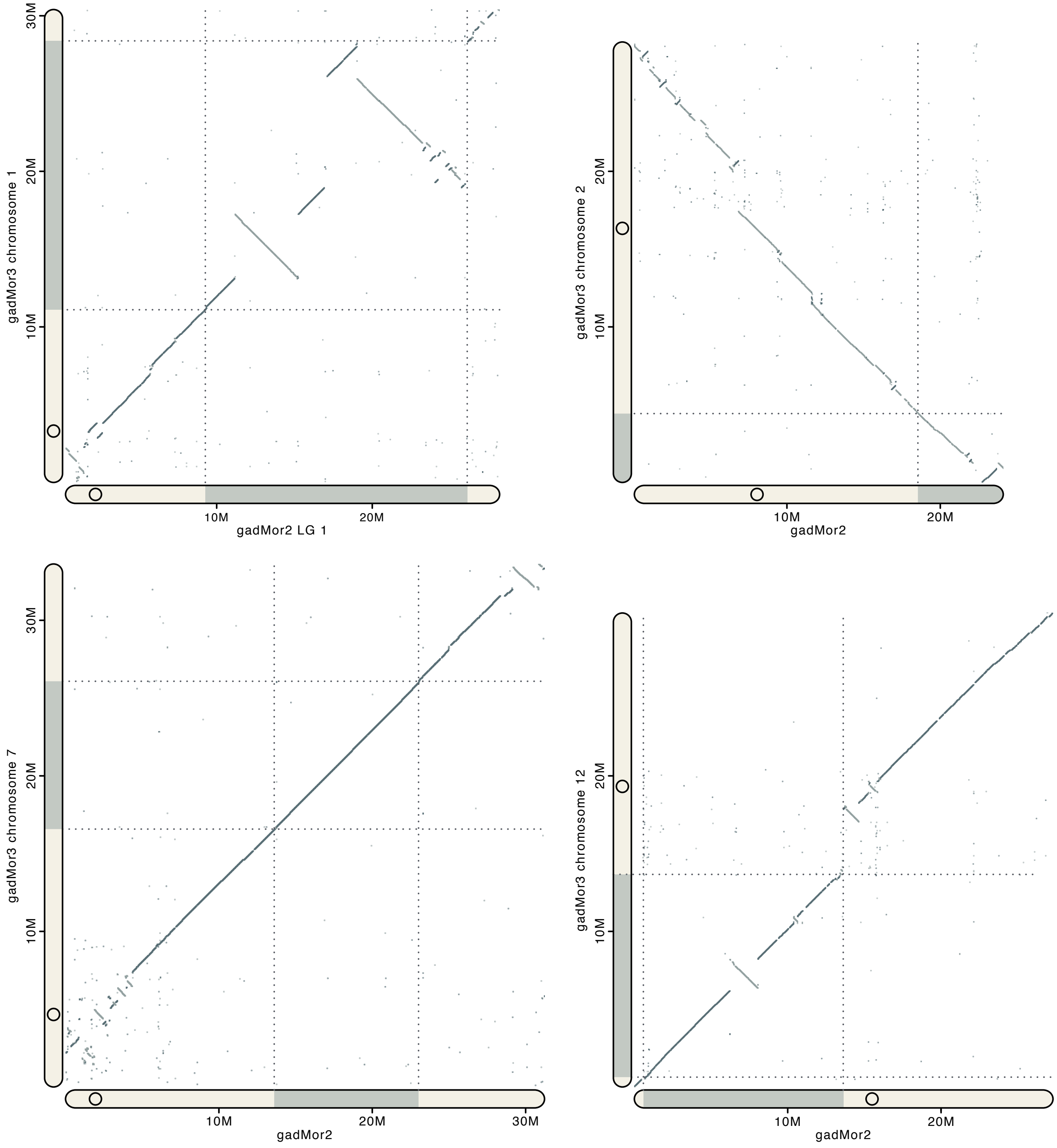

Dot plots illustrate regions of homology between the genome assemblies gadMor2[10] and gadMor3[36], for the four linkage groups with large genomic inversion regions in *Gadus morhua*. Grey

shading and dotted lines indicate the beginning and end of supergene regions. Circles on scale bars indicate approximate centromere positions[36].

Supplementary Figure 2: Divergence times among *Gadus morhua* populations.

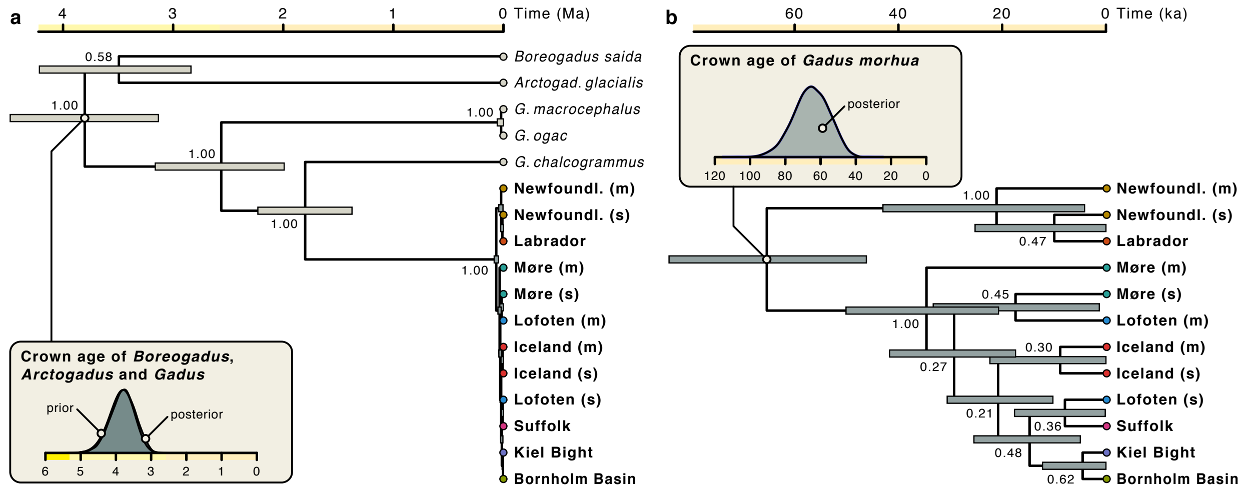

a Maximum-clade-credibility tree of *Gadus morhua* populations and outgroups inferred from genome-wide SNPs while accounting for incomplete lineage sorting with SNAPP. Single-nucleotide polymorphisms from within the four supergenes were excluded in the inference. *Gadus morhua* populations are marked in bold; regular font is used for species here considered as outgroups. The tree was time-calibrated with a single prior distribution to constrain the crown age of *Gadus*, *Arctogadus*, and *Boreogadus* to around 3.83 Ma (standard deviation: 0.093), according to the results of our analysis of divergence times among the three genera with the isolation-with-migration model (Extended Data Fig. 2a; Supplementary Figure 6). The prior distribution is shown in the inset together with the posterior distribution for the same divergence event; these two distributions are nearly identical. **b** Enlarged section of the maximum-clade-credibility tree showing divergences among *Gadus morhua* populations in more detail. The posterior distribution for the crown age of *Gadus morhua* is shown in the inset. In both **a** and **b**, numbers next to nodes report BPP support values and node bars indicate 95% HPD intervals for node ages. This tree is identical to the maximum-clade-credibility tree plotted in Figure 2b.

Supplementary Figure 3: Divergence times among *Gadus morhua* populations, specific to supergenes.

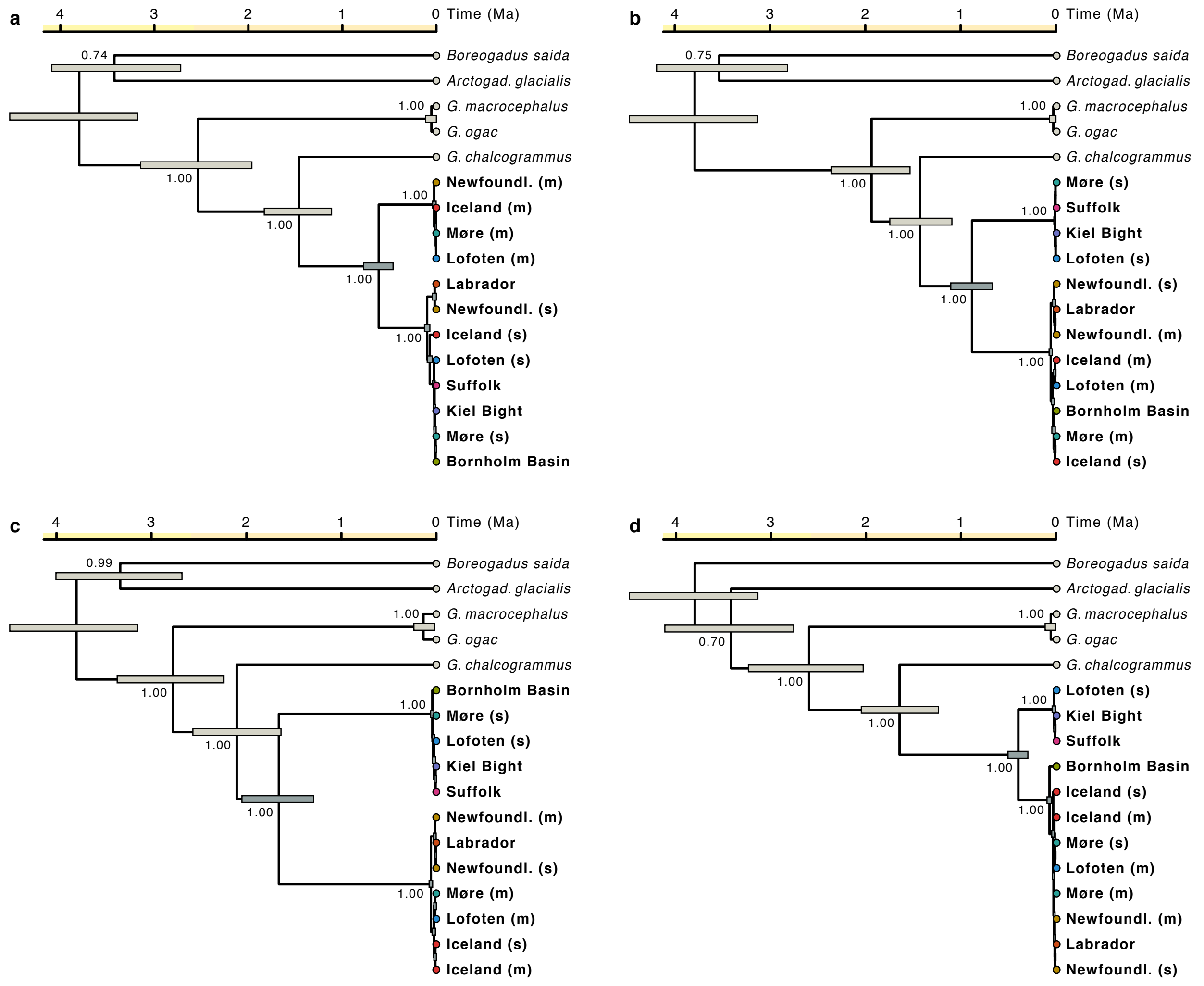

Maximum-clade-credibility trees of *Gadus morhua* populations and outgroups, separately inferred with SNAPP for each of the four supergenes. Inference settings were identical to those used to infer the MCC tree shown in Supplementary Figure 2, except that only SNPs from supergene regions were used. **a**–**d** Maximum-clade-credibility trees for the supergene on linkage groups (LGs) 1 (**a**), 2 (**b**), 7 (**c**), and 12 (**d**). Numbers next to nodes report BPP support values and node bars indicate 95% HPD intervals for node ages. These trees are identical to the ones shown in Figure 3a,d,g,j.

Supplementary Figure 4: Between-population divergence along linkage groups.

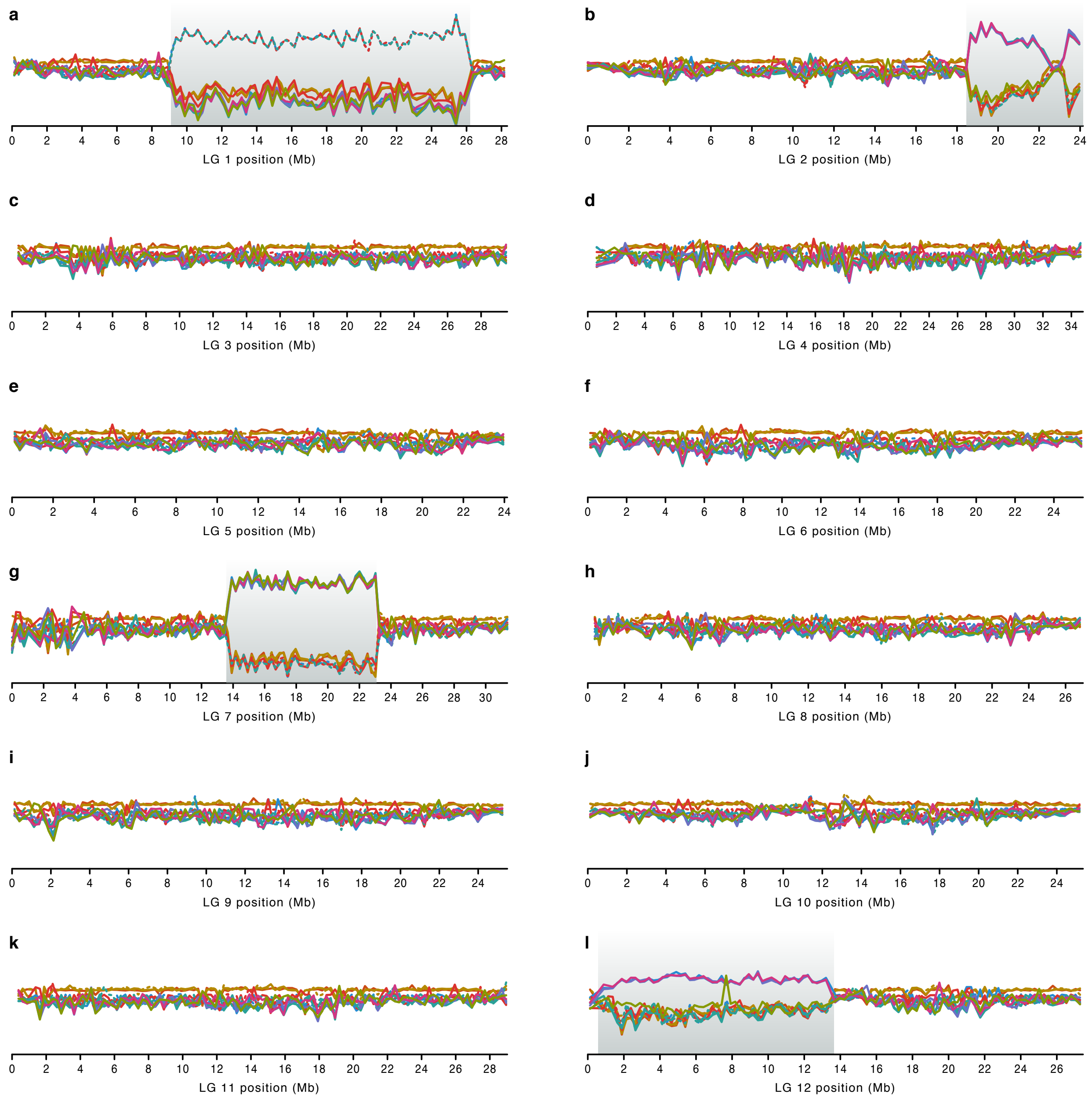

Divergence times were estimated with SNAPP from SNPs in sliding windows. **a–l** Between-population mean divergence-time estimates along LGs 1 (**a**), 2 (**b**), 3 (**c**), 4 (**d**), 5 (**e**), 6 (**f**), 7 (**g**), 8 (**h**), 9 (**i**), 10 (**j**), 11 (**k**), and 12 (**l**), plotted as in Figure 4. The four supergene regions on LGs 1, 2, 7, and 12 are indicated with grey background.

Supplementary Figure 4 (continued): Between-population divergence along linkage groups.

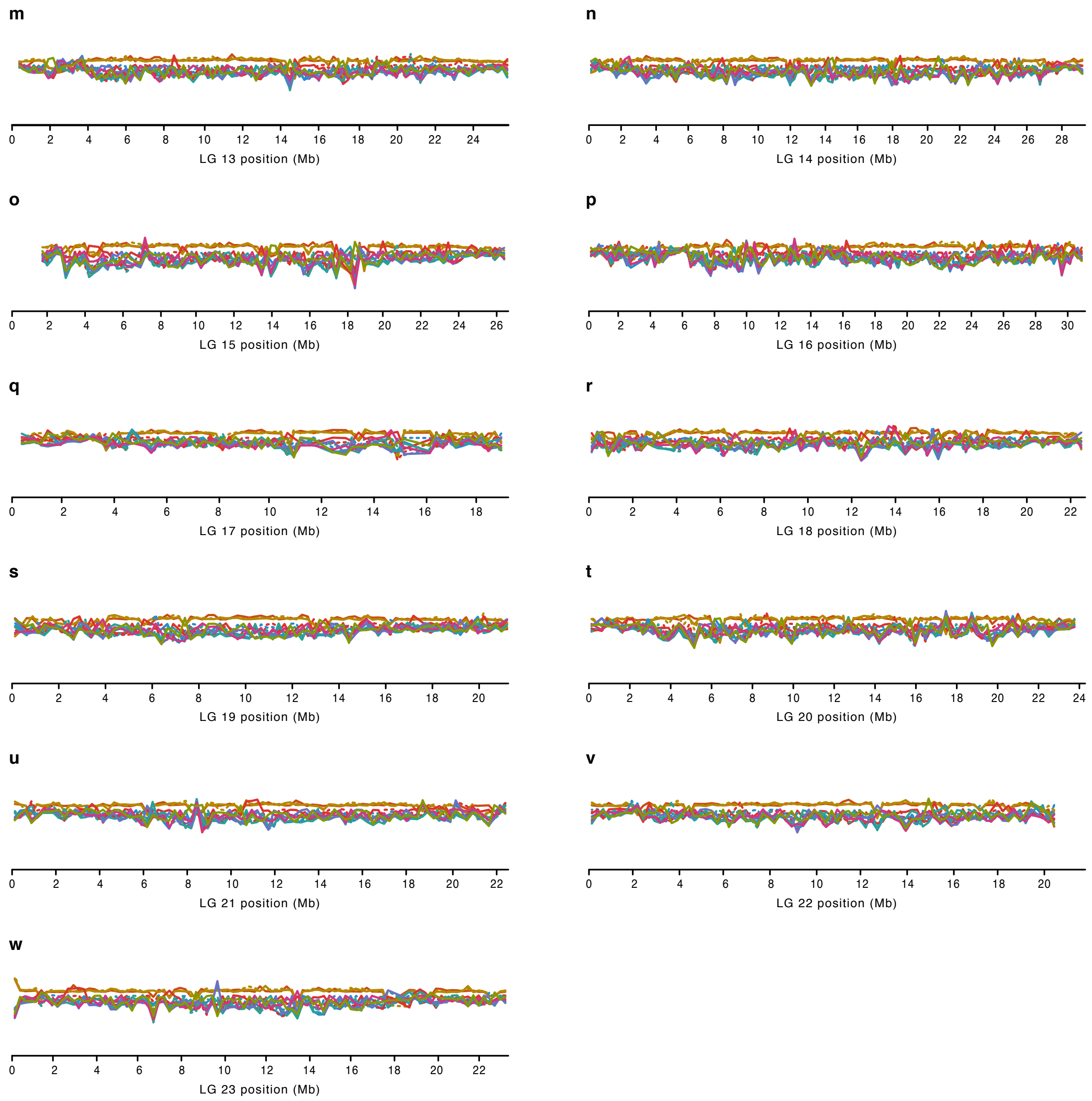

m–w Between-population divergence times along LGs 13 (**m**), 14 (**n**), 15 (**o**), 16 (**p**), 17 (**q**), 18 (**r**), 19 (**s**), 20 (**t**), 21 (**u**), 22 (**v**), and 23 (**w**).

Supplementary Figure 5: Divergence times of Gadinae.

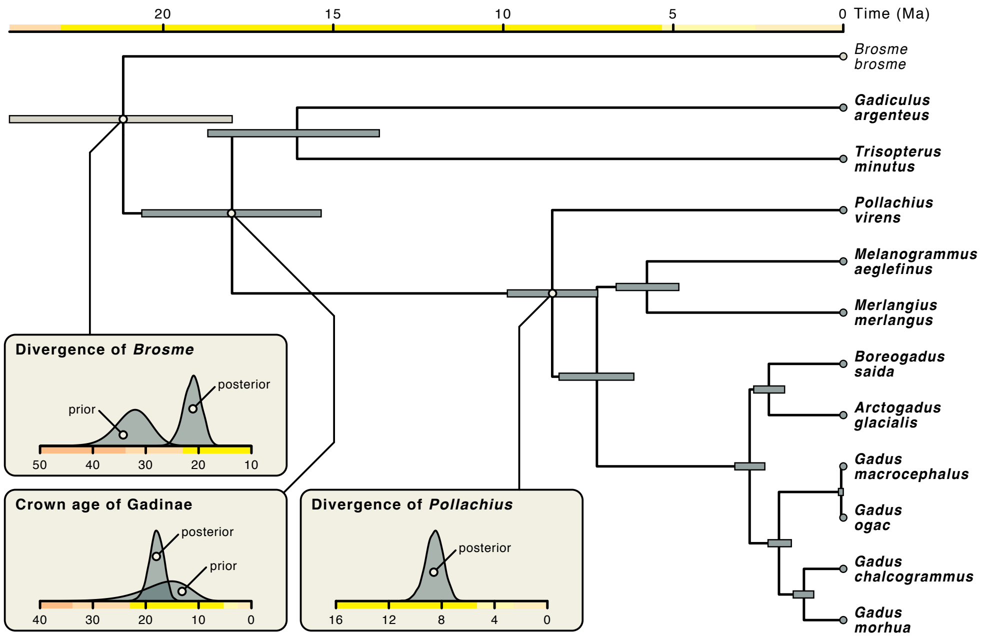

Maximum-clade-credibility species tree of Gadinae estimated with StarBEAST from 91 gene alignments under the multi-species coalescent model. Species included in the subfamily Gadinae are marked in bold. The tree was time-calibrated with two prior distributions according to Musilova et al.[37], constraining the divergence of the outgroup *Brosme brosme* to have occurred around 32.325 Ma (standard deviation: 0.1), and the first divergence within crown Gadinae to have occurred around 18.1358 Ma (standard deviation: 0.28). These prior distributions are shown in insets together with the respective posterior distributions; also shown is the posterior distribution for the divergence time of *Pollachius virens*, which we used for time calibration of the downstream estimation of divergence times among *Gadus*, *Arctogadus*, and *Boreogadus* with the isolation-with-migration model (Extended Data Fig. 2; Supplementary Figure 6). Node bars indicate 95% highest-posterior-density (HPD) intervals for node ages. All nodes were supported by Bayesian posterior probabilities (BPP) of 1.0.

Supplementary Figure 6: Divergence times and introgression among *Gadus*, *Arctogadus*, and *Boreogadus*.

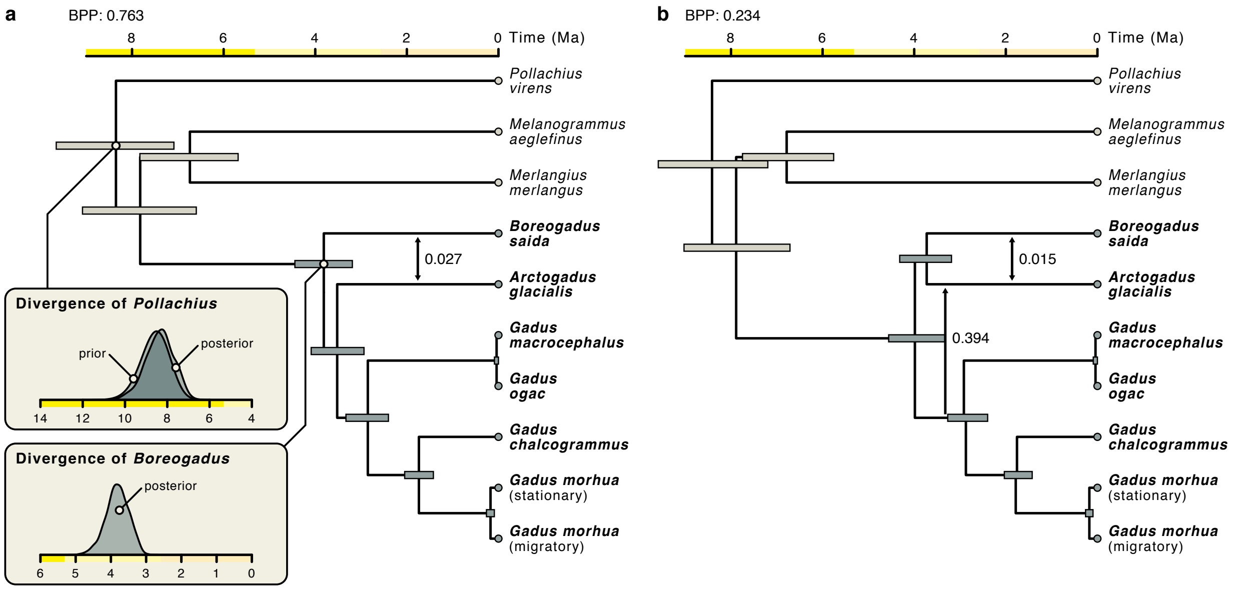

Maximum-clade-credibility species trees estimated with the AIM package for BEAST 2 under the isolation-with-migration model. Species of the three genera *Gadus*, *Arctogadus*, and *Boreogadus* are marked in bold; regular font is used for species considered as outgroups in this analysis. The tree was time-calibrated with a single prior distribution according to the results of our analysis of divergence times of Gadinae (Supplementary Figure 5), constraining the divergence of *Pollachius* to have occurred around 8.56 Ma (standard deviation: 0.08). Almost the entire posterior tree distribution (99.7%) supported one out of two constellations of topology and introgression. **a** Constellation of topology and introgression supported by a BPP of 0.763. The arrow between the branches leading to *Boreogadus saida* and *Arctogadus glacialis* indicates introgression supported with a Bayes factor greater than 10, the rate of which was estimated at 0.027 lineages per million years (95% HPD: 0–0.059). Insets show the prior distribution placed on the age of the divergence of *Pollachius virens*, the resulting posterior distribution for the same divergence event, and the posterior distribution for the divergence time of *Boreogadus saida*, which we used for time calibration in the downstream analyses of divergence times among *Gadus morhua* population. **b** Constellation of topology and introgression supported by a BPP of 0.234. Bayes factors greater than 10 support introgression between two pairs of lineages: from the common ancestor of the genus *Gadus* to *Arctogadus glacialis* with an estimated rate of 0.394 lineages per million years (95% HPD: 0.245–0.601) and between *Boreogadus saida* and *Arctogadus glacialis* with an estimated rate of 0.015 lineages per million years (95% HPD: 0–0.059). In both **a** and **b**, node bars indicate 95% HPD intervals for node ages.

Supplementary Figure 7: Distribution ranges of sampled species of the genera *Gadus*, *Arctogadus*, and *Boreogadus*.

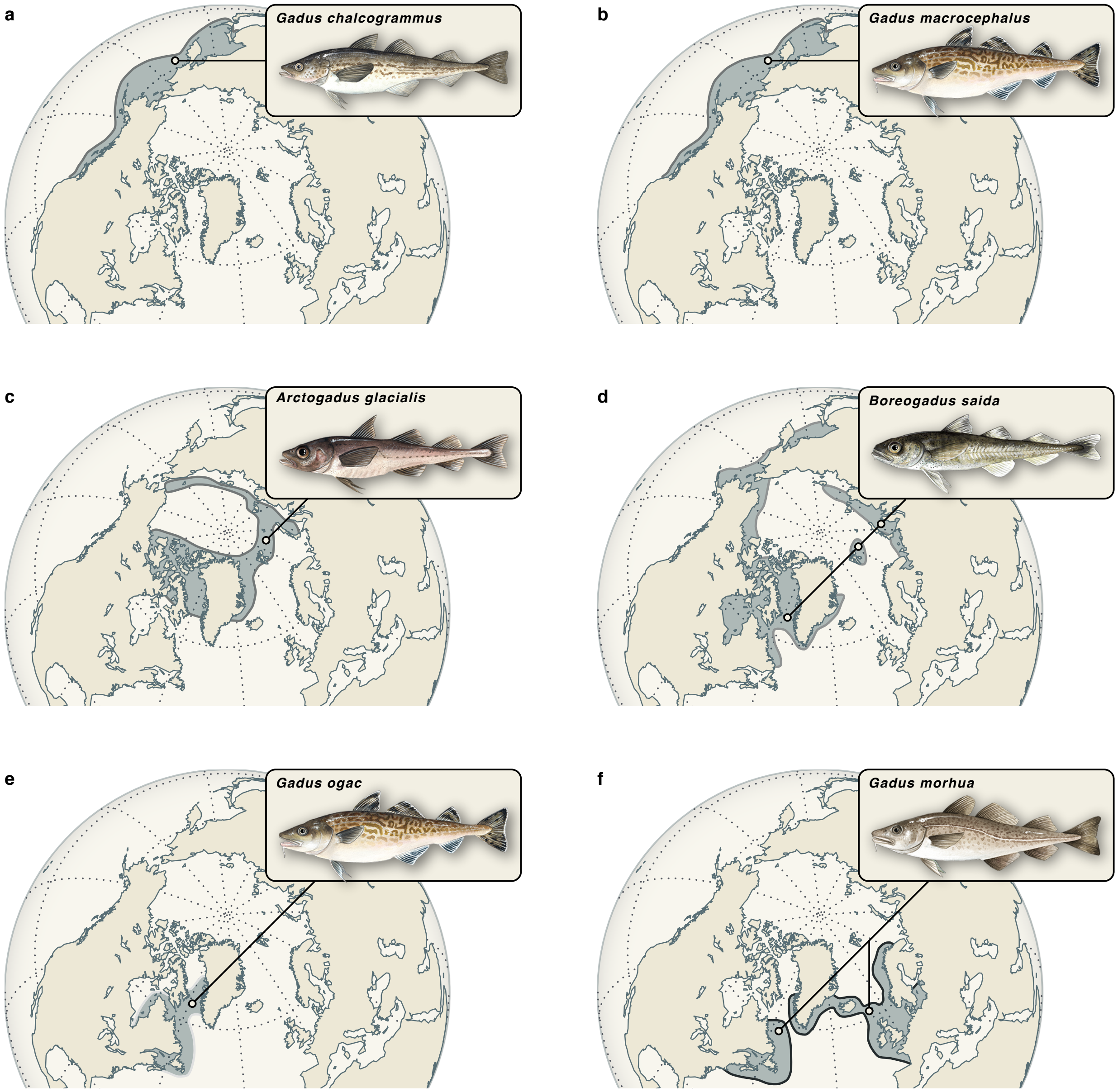

The distributions of the six species *G. chalcogrammus*, *G. macrocephalus*, *A. glacialis*, *B. saida*, *G. ogac*, and *G. morhua* are as shown in Extended Data Fig. 2a but displayed separately.

Supplementary Figure 8: Population sizes over time estimated with Relate from simulated data.

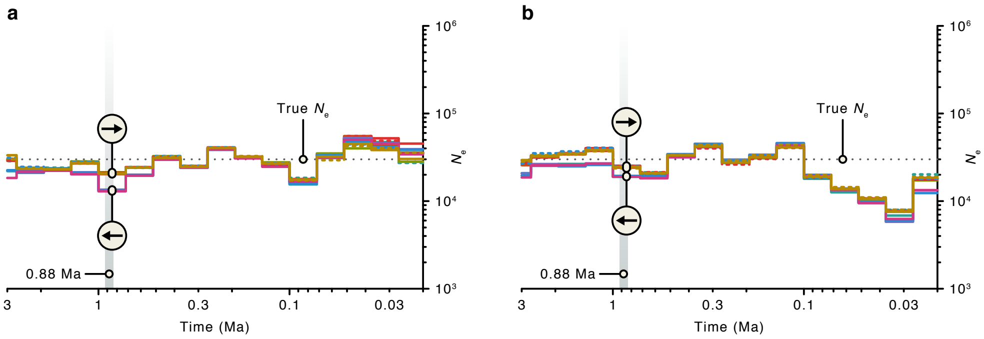

To test the ability of Relate to recover bottleneck events associated with old genomic inversions, we simulated chromosome-scale genetic data under a model of population diversification with msprime v.0.7.4[21], and inferred population sizes over time from these data with Relate. The selected model mimicked diversification of *Gadus morhua* populations as inferred from the supergene on LG 2 (Fig. 3d), with an initial divergence separating two haplotypes (labelled with forward and reverse arrows) 880,823 years ago, immediately followed by an extreme bottleneck in one of the two descending lineages during which the population size was reduced to a single sequence. The length of the bottleneck was set to ten generations. Outside of the bottleneck, all population sizes were set to a diploid size of $N_e = 30,000$. The generation time was set to 10 years and mutations were added with a rate identical to the one inferred for our empirical dataset ($\mu = 1.64 \times 10^{-9}$ per bp and year). To model recombination rate variation in realistic detail we used a recombination map for a human chromosome from the HapMap project[38] because we assumed that the resolution of these maps would exceed those of available maps for teleost fishes. We selected the recombination map for human chromosome 20, which did not contain large regions without information and was more comparable in size to fish chromosomes than other maps from the HapMap project. **a,b** The inference of population sizes over time with Relate used the same settings as our inference from empirical data (Fig. 3f), except that, to allow an assessment of the effect of unknown recombination rate variation, we inferred population sizes from the simulated data once with the true recombination map as input to Relate (**a**) and once assuming homogeneous recombination across the chromosome (**b**). The dotted line shows the true population size used in simulations and the grey region indicates the time of the simulated bottleneck (the width of this region is not drawn to scale). Regardless of whether the true recombination map was used or homogenous recombination was assumed, the signature of the bottleneck was recognizable as a separation of the estimated population sizes per haplotype around the time of the bottleneck, with temporarily decreased estimates for the populations carrying the affected haplotype (marked with a reverse arrow). The true depth of the simulated bottleneck (an N_e corresponding to a single sequence), however, was not recovered, probably because Relate estimates population sizes in time bins that are much wider than the duration of the bottleneck, spanning up to several hundred thousand years. In most time bins outside the bottleneck, the inferred population size was close to the true N_e used in the simulation.

Supplementary Figure 9: Tree-based signals for introgression among *Gadus*, *Arctogadus*, and *Boreogadus*.

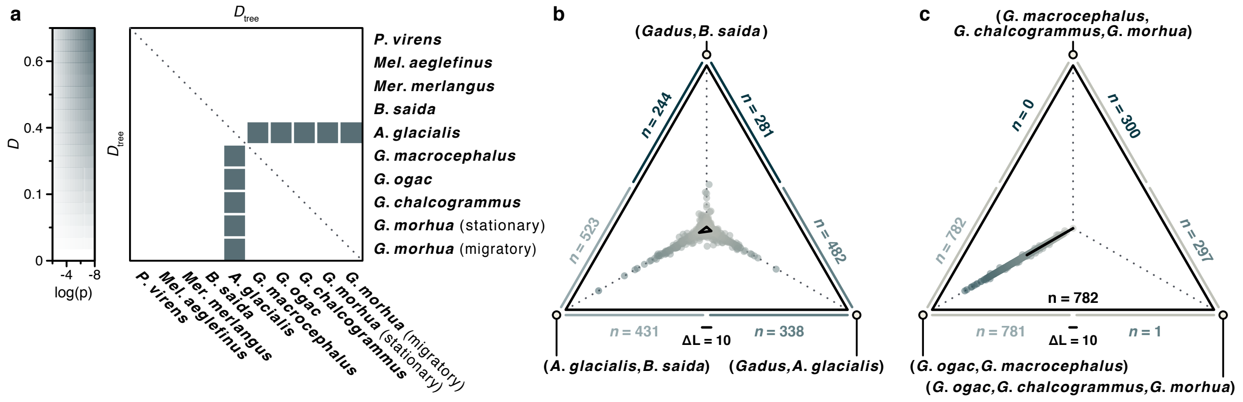

Signals of introgression among the genera *Gadus*, *Arctogadus*, and *Boreogadus*, obtained from sets of maximum-likelihood phylogenies. **a** The heatmap shows the tree-based equivalent to Patterson's D-statistic, D_{tree}[30], and its significance. Significant results ($p < 10^{-8}$) were only found in trios where *Boreogadus saida* and *Arctogadus glacialis* were placed as sister taxa, P1 and P2, and one of the species of the genus *Gadus* was in the position of the third species, P3. The grouping of P1 and P2 was then supported by 203 trees while 145 alignments supported the grouping of P2 and P3 and only 25 alignments supported the grouping of P1 and P3. The D_{tree}-statistic was thus the same for all these trios; $D_{\mathrm{tree}} = (145 - 25)/(145 + 25) = 0.706$. **b** Likelihood support from alignments sampled across the genome for three alternative positions of *Arctogadus glacialis*: As the sister to *Boreogadus saida* (bottom left), as the sister to the genus *Gadus* (bottom right), and as an outgroup to a clade combining *Boreogadus saida* and the genus *Gadus* (top). Each dot in the triangle plot indicates the relative likelihood support that one alignment provides for each of the three hypotheses. Per dot, the distance from the center corresponds to the difference in likelihood between the best-supported topology and the alternative ones; the scale bar indicates the distance corresponding to a difference in log-likelihood support of 10. The small triangle in the center of the plot indicates the mean relative likelihood support for each topology, across all alignments. Sample sizes given on the edges of the triangle report the numbers of alignments that support one of the topologies connected by the edge over the other one. **c** As **b** but comparing the positions of *Gadus ogac* as the sister of *Gadus macrocephalus*, as the sister to *Gadus chalcogrammus* and *Gadus morhua*, and as the outgroup to a clade formed by *Gadus macrocephalus*, *Gadus chalcogrammus*, and *Gadus morhua*.

The tree-based tests for introgression corroborate the identification of *Arctogadus glacialis* as a species that introgressed either with *Boreogadus saida* or with the common ancestor of the genus *Gadus*, but do not further support the evidence coming from Patterson's D-statistic for introgression between *Gadus ogac* and *Gadus morhua*, perhaps because the introgressed regions were in this case too short to affect tree topologies.

Supplementary Tables

Supplementary Table 1: Assembly completeness.
Assembly completeness was assessed with the program BUSCO v.5.0, using the Actinopterygii set of 3,640 conserved and single-copy orthologs.

Category	Frequency	Percent of total
Complete BUSCO genes	3,061	84.1
Complete and single-copy BUSCO genes	3,020	83.0
Complete and duplicated BUSCO genes	41	1.1
Fragmented BUSCO genes	218	6.0
Missing BUSCO genes	361	9.9

Supplementary Table 2: Genetic distances between gadid genome assemblies.
Genetic distances were calculated from the three-way whole-genome alignment in which the gadMor_Stat and melAeg assemblies were aligned to the gadMor2 assembly. The three-way alignment was carefully filtered for reliably aligned sites, and pairwise genetic distances were calculated as the proportion of sites considered reliably aligned that differed between a pair of assemblies. We report, per gadMor2 linkage group, the genetic distances between the gadMor_Stat and gadMor2 assemblies ($d_{\text{gadMor\_Stat}-\text{gadMor2}}$), the genetic distance between the melAeg and gadMor2 assemblies ($d_{\text{melAeg}-\text{gadMor2}}$), and the relative difference between the gadMor_Stat and gadMor2 assemblies, compared to the difference between the melAeg and gadMor2 assemblies ($d_{\text{gadMor\_Stat}-\text{gadMor2}}$ / $d_{\text{melAeg}-\text{gadMor2}}$).

LG	$d_{\text{gadMor\_Stat}-\text{gadMor2}}$	$d_{\text{melAeg}-\text{gadMor2}}$	$d_{\text{gadMor\_Stat}-\text{gadMor2}}/d_{\text{melAeg}-\text{gadMor2}}$
1	0.00833	0.04675	0.17816
2	0.00615	0.05136	0.11980
3	0.00454	0.04696	0.09659
4	0.00534	0.05212	0.10251
5	0.00459	0.04726	0.09701
6	0.00411	0.04564	0.08994
7	0.00681	0.04756	0.14327
8	0.00471	0.04842	0.09723
9	0.00407	0.04752	0.08560
10	0.00443	0.05032	0.08800
11	0.00423	0.04523	0.09362
12	0.00581	0.04941	0.11756
13	0.00401	0.04782	0.08386
14	0.00404	0.04637	0.08720
15	0.00421	0.04504	0.09356
16	0.00419	0.04636	0.09040
17	0.00518	0.05474	0.09462
18	0.00436	0.04926	0.08849
19	0.00450	0.05136	0.08759
20	0.00435	0.04563	0.09539
21	0.00449	0.05026	0.08942
22	0.00486	0.04998	0.09714
23	0.00466	0.04954	0.09411

LG	Region	$d_{\text{gadMor\_Stat}-\text{gadMor2}}$	$d_{\text{melAeg}-\text{gadMor2}}$	$d_{\text{gadMor\_Stat}-\text{gadMor2}}/d_{\text{melAeg}-\text{gadMor2}}$
1	9,114,741–26,192,489	0.01069	0.04504	0.23736
2	18,489,307–24,050,282	0.01285	0.05805	0.22141
7	13,606,502–23,016,726	0.00909	0.04257	0.21348
12	589,105–13,631,347	0.00658	0.04868	0.13523

Supplementary Table 3: Contigs of the gadMor_Stat and melAeg assemblies aligning near supergene boundaries.
Contigs were aligned to the gadMor2 assembly[10] with BLASTN searches. Per alignment, we report the start and end positions of aligning regions on both the gadmor2 assembly (in the form "LG:start position–end position") and on the contigs from the gadMor_Stat and melAeg[39] assemblies. Contig labels correspond to those used in Figure 1b.

Label	Assembly	Contig	gadMor2 position	Contig position	Orientation
1	gadMor_Stat	scf7180000174049	1:8,943,539–9,128,372	1–200,341	forward
			1:16,848,173–16,296,669	200,341–707,031	reverse
			1:26,322,778–26,326,857	706,985–710,054	forward
2	gadMor_Stat	scf7180000173314	1:9,130,274–9,192,260	253,972–314,079	forward
			1:18,622,815–18,890,477	254–258,023	forward
3	melAeg	scf1854:1-47276	1:9,090,044–9,120,848	4–47,275	forward
4	melAeg	scg895:1-207496	1:9,130,297–9,187,321	1–59,877	reverse
			1:18,731,791–18,890,477	56,307–205,627	reverse
5	gadMor_Stat	scf7180000174960	1:26,199,094–26,219,392	22–28,398	forward
6	gadMor_Stat	scf7180000173351	2:18,399,575–18,494,225	1–89,835	forward
7	gadMor_Stat	scf7180000176322	2:18,567,231–18,487,151	6–75,867	reverse
			2:23,150,464–23,200,546	68,286–108,674	reverse
8	melAeg	scf170:27711-284173	2:18,360,264–18,642,641	1–255901	forward
9	gadMor_Stat	scf7180000175069	2:23,939,789–24,054,399	4–107,435	forward
10	melAeg	scf874:12556-53982	2:24,007,915–24,049,209	1–39,552	reverse
11	gadMor_Stat	scf7180000173083	7:13,241,739–13,642,122	1–403,620	reverse
12	gadMor_Stat	scf7180000173082	7:13,645,465–13651003	30,270–37,901	reverse
			7:22,971,316–23,002,424	4–28,473	forward
13	gadMor_Stat	scf7180000177071	7:13,652,432–13,689,724	33–23,593	reverse
			7:23,269,226–23,270,717	21,916–23,360	forward
14	melAeg	scf271:1-285897	7:13,585,625–13,651,514	6–66,452	forward
			7:22,773,521–23,002,439	68,044–285,897	reverse
15	gadMor_Stat	scf7180000175147	7:23,043,967–23,074,768	1–31,361	forward
16	gadMor_Stat	scf7180000176674	12:579,198–607,782	20,228–51,625	reverse
			12:13,378,418–13,386,293	1–10,649	forward
17	gadMor_Stat	scf7180000174053	12:662,878–694,568	8–27,278	forward
			12:13,614,908–13,616,907	23,719–25,709	reverse

Supplementary Table 4: *Gadus morhua* specimens used for population-level analyses.
For the Newfoundland, Iceland, Møre, and Lofoten populations, we distinguished "migratory" (m) and "stationary" (s) individuals. At all other sampling locations, all individuals were considered stationary. Coverage was measured based on an assumed genome size of 650 Mbp[10]. TL: terminal length in centimeters.

Specimen ID	ENA accession(s)	Population	Country	Lat.	Lon.	Date	Sampling	TL (cm)	# reads	Coverage
TWI1307Z12	ERR5321892	Newfoundland (m)	Canada	49.68	-54.80	07/2013	Hand line	76	66,970,106	10.3
TWI1307Z17	ERR5321893	Newfoundland (m)	Canada	49.68	-54.80	07/2013	Hand line	70	80,309,398	12.4
TWI1307Z07	ERR5321894	Newfoundland (s)	Canada	49.68	-54.80	07/2013	Hand line	51	76,098,264	11.7
TWI1307Z15	ERR5321895	Newfoundland (s)	Canada	49.68	-54.80	07/2013	Hand line	67	66,823,400	10.3
BAT1309Z03	ERR5321896	Labrador	Canada	52.27	-55.58	09/2013	Hand line	55	60,329,852	9.3
BAT1309Z10	ERR5321897	Labrador	Canada	52.27	-55.58	09/2013	Hand line	62	55,045,914	8.5
ICO0304Z23	ERR5321898	Iceland (m)	Iceland	63.50	-22.11	04/2003	Gillnet	86	110,796,682	17.0
ICO0304Z20	ERR5321899	Iceland (m)	Iceland	63.50	-22.11	04/2003	Gillnet	81	69,704,806	10.7
ICC0304Z11	ERR5321900	Iceland (s)	Iceland	63.81	-21.98	04/2003	Gillnet	NA	57,098,960	8.8
LOF1103Z11	ERR5321901	Lofoten (m)	Norway	68.07	13.58	03/2011	Longline	93	67,562,048	10.4
NA	ERR1551885[1]	Lofoten (m)	Norway	67.60	12.54	NA	Longline	NA	483,953,674	74.5
LOF1106Z04	ERR5321902	Lofoten (s)	Norway	68.07	13.69	06/2011	Hand line	64	57,883,292	8.9
LOF1106Z24	ERR5321903	Lofoten (s)	Norway	68.07	13.69	06/2011	Hand line	68	60,765,564	9.3
LOF1106Z11[2]	ERR5321904	Lofoten (s)	Norway	68.07	13.69	06/2011	Hand line	62	61,641,456	9.5
AVE1403Z10	ERR5321905	Møre (m)	Norway	63.08	7.11	03/2014	Gillnet	80	63,759,112	9.8
AVE1403Z11	ERR5321906	Møre (m)	Norway	63.08	7.11	03/2014	Gillnet	102	106,219,192	16.3
AVE1409Z09[3]	ERR2850387	Møre (s)	Norway	63.12	7.23	09/2014	Hand line	74	57,939,902	8.9
AVE1409Z10[3]	ERR2850388	Møre (s)	Norway	63.12	7.23	09/2014	Hand line	65	60,093,966	9.2
LOW1503Z06[3]	ERR2850515-18	Suffolk	UK	52.12	1.98	03/2015	Trawling	58	68,126,966	10.5
LOW1504Z07[3]	ERR2850579-82	Suffolk	UK	52.04	1.51	04/2015	Trawling	53	67,523,264	10.4
KIE1102Z06[3]	ERR2850464	Kiel Bight	Germany	54.76	10.05	02/2011	Trawling	51	79,339,236	12.2
KIE1103Z20[3]	ERR2850485	Kiel Bight	Germany	54.76	10.05	03/2011	Trawling	42	89,153,264	13.7
BOR1205Z03[3]	ERR2850424-26	Bornholm Basin	Sweden	55.29	15.72	05/2012	Trawling	38	52,748,670	8.1
BOR1205Z07[3]	ERR2850436-38,	Bornholm Basin	Sweden	55.29	15.72	05/2012	Trawling	45	54,990,446	8.5

[1]Accession of Illumina data from the migratory *Gadus morhua* specimen used for the gadMor2 assembly[10]
[2]Stationary *Gadus morhua* individual used for the gadMor_Stat assembly
[3]Published in Barth et al.[40]

Supplementary Table 5: Genetic diversity of *Gadus morhua* populations and outgroups. Genetic diversity π was calculated following Ruegg et al.[41]. Genetic diversities for populations inferred to be carriers of the derived arrangement are underlined.

		π outside of supergene	π in supergene regions ($\times 10^{-3}$)			
Species	Population	regions ($\times 10^{-3}$)	LG 1	LG 2	LG 7	LG 12
G. macrocephalus	NA	0.363	0.302	0.283	0.369	0.376
G. ogac	NA	0.323	0.299	0.268	0.316	0.365
G. chalcogrammus	NA	0.905	1.031	0.993	1.213	1.068
G. morhua	Newfoundland (m)	0.934	0.522	0.593	0.799	0.707
G. morhua	Newfoundland (s)	0.934	0.768	0.594	0.789	0.711
G. morhua	Labrador	0.942	0.630	0.611	0.808	0.723
G. morhua	Iceland (m)	0.942	0.353	0.578	0.782	0.678
G. morhua	Iceland (s)	0.882	0.846	0.550	0.680	0.652
G. morhua	Lofoten (m)	1.084	0.394	0.665	0.871	0.776
G. morhua	Lofoten (s)	1.061	0.843	0.683	0.812	0.489[1]
G. morhua	Møre (m)	0.947	0.371	0.589	0.746	0.700
G. morhua	Møre (s)	0.974	0.780	0.619	0.694	0.660
G. morhua	Suffolk	0.947	0.753	0.608	0.729	0.532[1]
G. morhua	Kiel Bight	0.935	0.738	0.607	0.769	0.518[1]
G. morhua	Bornholm Basin	0.904	0.722	0.379	0.680	0.487
G. morhua	All with derived arrangement[2]	NA	0.410	0.629	0.782	0.513
G. morhua	All with ancestral arrangement[3]	NA	0.760	0.570	0.737	0.701

[1]The derived and ancestral arrangements for the supergene on LG 12 could not be identified with the contig-mapping approach; however, reduced population size in a time inverval after the supergene origin suggests that it was the individuals from the Lofoten (s), Suffolk, and Kiel Bight populations that carried the derived arrangments.

[2]Average genetic diversity across all populations carrying the derived arrangement

[3]Average genetic diversity across all populations carrying the ancestral arrangement

Supplementary Table 6: D_{BBAA}-statistics for *Gadus morhua* populations and outgroups. For the calculation of the D_{BBAA} version of the D-statistic[27], all trios are oriented so that the number of "BBAA" patterns (C_{BBAA}) is greater than the numbers of "ABBA" (C_{ABBA}) and "BBAA" (C_{BABA}) patterns. Additionally, the trio is arbitrarily oriented so that the number of "ABBA" patterns (C_{ABBA}) exceeds that of "BBAA" patterns (C_{BABA}). When the trio is oriented in this way, the D_{BBAA}-statistic is never negative and values significantly greater than zero support introgression between the two species in positions P2 and P3. If the same pair of species in positions P2 and P3 produced significant results with several third species in position P1, only the trio with the highest D_{BBAA} is listed. Significance was assessed with block-jackknifing. Sites from supergene regions were excluded from this analysis. The highest D values are underlined.

P1	P2	P3	C_{BBAA}	C_{ABBA}	C_{BABA}	D_{BBAA}	p
Lofoten (m)	*G. chalcogrammus*	*G. macrocephalus*	520,663.0	99,351.4	84,913.6	0.078	0.0000
Lofoten (m)	Newfoundland (m)	*G. macrocephalus*	1,315,150.0	13,951.5	12,893.6	0.039	0.0000
Lofoten (m)	Newfoundland (s)	*G. macrocephalus*	1,315,950.0	13,995.8	12,968.8	0.038	0.0000
Lofoten (m)	Labrador	*G. macrocephalus*	1,283,140.0	14,079.2	13,066.8	0.037	0.0000
Lofoten (m)	Møre (m)	*G. macrocephalus*	1,359,260.0	13,246.6	12,503.9	0.029	0.0000
Lofoten (m)	Møre (s)	*G. macrocephalus*	1,290,310.0	12,728.2	12,083.8	0.026	0.0000
Lofoten (m)	Iceland (m)	*G. macrocephalus*	1,360,800.0	13,236.4	12,506.8	0.028	0.0000
Lofoten (m)	Iceland (s)	*G. macrocephalus*	1,195,570.0	11,584.5	10,701.2	0.040	0.0000
Lofoten (m)	Lofoten (s)	*G. macrocephalus*	1,271,080.0	13,134.9	12,618.3	0.020	0.0000
Lofoten (m)	Suffolk	*G. macrocephalus*	1,337,050.0	13,094.1	12,316.8	0.031	0.0000
Lofoten (m)	Kiel Bight	*G. macrocephalus*	1,349,250.0	13,088.5	12,375.4	0.028	0.0000
Lofoten (m)	Bornholm Basin	*G. macrocephalus*	1,249,840.0	12,723.0	12,013.0	0.029	0.0000
G. macrocephalus	*G. ogac*	*G. chalcogrammus*	1,381,720.0	11,877.9	9,868.6	0.092	0.0000
G. macrocephalus	*G. ogac*	Newfoundland (m)	1,407,000.0	16,512.3	9,997.9	0.246	0.0000
G. macrocephalus	*G. ogac*	Newfoundland (s)	1,409,330.0	16,543.0	9,954.9	<u>0.249</u>	0.0000
G. macrocephalus	*G. ogac*	Labrador	1,362,790.0	16,024.5	9,682.8	0.247	0.0000
G. macrocephalus	*G. ogac*	Møre (m)	1,428,900.0	16,894.6	10,179.4	0.248	0.0000
G. macrocephalus	*G. ogac*	Møre (s)	1,337,080.0	15,864.3	9,578.4	0.247	0.0000
G. macrocephalus	*G. ogac*	Lofoten (m)	1,243,990.0	14,961.9	8,992.1	0.249	0.0000
G. macrocephalus	*G. ogac*	Iceland (m)	1,433,930.0	16,947.1	10,201.1	0.248	0.0000
G. macrocephalus	*G. ogac*	Iceland (s)	1,244,640.0	14,521.0	8,801.2	0.245	0.0000
G. macrocephalus	*G. ogac*	Lofoten (s)	1,306,910.0	15,591.3	9,381.0	0.249	0.0000
G. macrocephalus	*G. ogac*	Suffolk	1,399,690.0	16,515.4	9,967.1	0.247	0.0000
G. macrocephalus	*G. ogac*	Kiel Bight	1,416,600.0	16,741.8	10,039.2	<u>0.250</u>	0.0000
G. macrocephalus	*G. ogac*	Bornholm Basin	1,303,870.0	15,412.2	9,298.4	0.247	0.0000
Lofoten (m)	Newfoundland (m)	*G. chalcogrammus*	899,928.0	19,121.2	17,064.7	0.057	0.0000
Lofoten (m)	Newfoundland (s)	*G. chalcogrammus*	900,322.0	19,129.8	17,145.6	0.055	0.0000
Lofoten (m)	Labrador	*G. chalcogrammus*	879,634.0	19,307.5	17,313.0	0.054	0.0000
Lofoten (m)	Møre (m)	*G. chalcogrammus*	940,601.0	17,992.4	16,643.0	0.039	0.0000
Lofoten (m)	Møre (s)	*G. chalcogrammus*	892,834.0	17,119.2	16,108.0	0.030	0.0000
Lofoten (m)	Iceland (m)	*G. chalcogrammus*	940,877.0	17,881.4	16,644.0	0.036	0.0000
Lofoten (m)	Iceland (s)	*G. chalcogrammus*	824,358.0	15,499.5	14,249.0	0.042	0.0000
Lofoten (m)	Lofoten (s)	*G. chalcogrammus*	880,879.0	17,736.3	16,817.5	0.027	0.0000
Lofoten (m)	Suffolk	*G. chalcogrammus*	925,054.0	17,621.4	16,512.5	0.032	0.0000
Lofoten (m)	Kiel Bight	*G. chalcogrammus*	933,388.0	17,530.1	16,578.2	0.028	0.0000
Lofoten (m)	Bornholm Basin	*G. chalcogrammus*	864,946.0	17,173.2	16,101.7	0.032	0.0000
Lofoten (m)	Møre (m)	Newfoundland (m)	101,116.0	76,042.8	73,359.0	0.018	0.0000

Supplementary Table 6 (continued): D_{BBAA}-statistics for *Gadus morhua* populations and outgroups.

P1	P2	P3	C_{BBAA}	C_{ABBA}	C_{BABA}	D_{BBAA}	p
Lofoten (m)	Møre (s)	Newfoundland (m)	97,687.3	73,463.8	71,353.9	0.015	0.0000
Newfoundland (s)	Newfoundland (m)	Lofoten (m)	100,337.0	73,708.3	73,166.3	0.004	0.0199
Lofoten (m)	Iceland (m)	Newfoundland (m)	99,598.9	76,880.4	73,436.2	0.023	0.0000
Lofoten (m)	Iceland (s)	Newfoundland (m)	87,416.8	67,254.8	64,090.8	0.024	0.0000
Lofoten (m)	Lofoten (s)	Newfoundland (m)	99,879.5	75,403.2	73,398.0	0.013	0.0000
Lofoten (m)	Suffolk	Newfoundland (m)	99,806.2	75,106.4	72,659.9	0.017	0.0000
Lofoten (m)	Kiel Bight	Newfoundland (m)	100,081.0	75,274.3	72,882.2	0.016	0.0000
Lofoten (m)	Bornholm Basin	Newfoundland (m)	95,876.1	72,700.8	71,137.0	0.011	0.0000
Lofoten (m)	Møre (m)	Newfoundland (s)	101,717.0	76,179.5	73,376.7	0.019	0.0000
Lofoten (m)	Møre (s)	Newfoundland (s)	98,290.6	73,381.3	71,418.9	0.014	0.0000
Lofoten (m)	Iceland (m)	Newfoundland (s)	100,066.0	77,109.0	73,368.2	0.025	0.0000
Lofoten (m)	Iceland (s)	Newfoundland (s)	87,938.8	67,313.5	64,024.0	0.025	0.0000
Lofoten (m)	Lofoten (s)	Newfoundland (s)	100,514.0	75,274.5	73,461.6	0.012	0.0000
Lofoten (m)	Suffolk	Newfoundland (s)	100,406.0	75,163.5	72,707.5	0.017	0.0000
Lofoten (m)	Kiel Bight	Newfoundland (s)	100,678.0	75,348.4	72,961.2	0.016	0.0000
Lofoten (m)	Bornholm Basin	Newfoundland (s)	96,373.6	72,764.8	71,170.7	0.011	0.0000
Lofoten (m)	Møre (m)	Labrador	101,470.0	76,990.3	73,939.4	0.020	0.0000
Lofoten (m)	Møre (s)	Labrador	98,044.0	74,081.3	71,929.0	0.015	0.0000
Newfoundland (s)	Labrador	Lofoten (m)	99,742.5	75,063.6	74,447.7	0.004	0.0279
Lofoten (m)	Iceland (m)	Labrador	99,959.3	77,749.7	73,957.8	0.025	0.0000
Lofoten (m)	Iceland (s)	Labrador	87,825.1	67,579.0	64,608.9	0.022	0.0000
Lofoten (m)	Lofoten (s)	Labrador	100,116.0	76,114.1	73,845.5	0.015	0.0000
Lofoten (m)	Suffolk	Labrador	100,325.0	75,694.3	73,373.0	0.016	0.0000
Lofoten (m)	Kiel Bight	Labrador	100,604.0	75,824.4	73,680.3	0.014	0.0000
Lofoten (m)	Bornholm Basin	Labrador	96,187.9	73,617.0	71,666.0	0.013	0.0000
Bornholm Basin	Møre (s)	Møre (m)	82,381.2	82,054.0	81,135.4	0.006	0.0001
Iceland (m)	Møre (m)	Lofoten (m)	86,643.9	84,266.6	82,604.6	0.010	0.0000
Bornholm Basin	Møre (m)	Iceland (m)	85,019.3	84,942.5	83,017.4	0.011	0.0000
Bornholm Basin	Møre (m)	Iceland (s)	73,998.2	73,801.4	72,299.6	0.010	0.0000
Bornholm Basin	Lofoten (s)	Møre (m)	85,727.1	83,660.2	83,127.0	0.003	0.0466
Lofoten (s)	Suffolk	Møre (m)	86,796.1	86,639.3	86,067.9	0.003	0.0241
Bornholm Basin	Kiel Bight	Møre (m)	89,156.6	82,461.2	81,638.7	0.005	0.0018
Bornholm Basin	Møre (s)	Lofoten (m)	81,360.8	77,833.7	76,767.9	0.007	0.0000
Bornholm Basin	Møre (s)	Iceland (m)	83,200.5	81,196.5	80,013.6	0.007	0.0000
Bornholm Basin	Møre (s)	Iceland (s)	73,138.3	71,373.7	70,332.3	0.007	0.0051
Bornholm Basin	Lofoten (s)	Møre (s)	82,126.2	81,588.3	80,904.9	0.004	0.0320
Lofoten (s)	Møre (s)	Suffolk	84,088.9	83,849.0	83,204.8	0.004	0.0021
Bornholm Basin	Kiel Bight	Møre (s)	85,114.4	80,467.4	79,193.1	0.008	0.0003
Suffolk	Møre (s)	Bornholm Basin	81,267.9	80,920.2	80,378.3	0.003	0.0523
Iceland (m)	Lofoten (s)	Lofoten (m)	84,955.3	84,141.1	82,642.6	0.009	0.0000
Iceland (m)	Suffolk	Lofoten (m)	85,394.6	83,070.4	81,713.4	0.008	0.0000
Iceland (m)	Kiel Bight	Lofoten (m)	85,257.8	83,440.5	82,256.6	0.007	0.0000
Lofoten (s)	Suffolk	Iceland (m)	87,585.4	86,223.8	84,759.5	0.009	0.0000
Bornholm Basin	Kiel Bight	Iceland (m)	90,081.5	81,472.2	80,596.6	0.005	0.0017
Bornholm Basin	Lofoten (s)	Iceland (s)	75,788.9	72,473.6	71,901.6	0.004	0.0698
Bornholm Basin	Suffolk	Iceland (s)	73,774.8	72,801.1	71,399.4	0.010	0.0001
Bornholm Basin	Kiel Bight	Iceland (s)	78,755.6	71,169.0	70,455.3	0.005	0.0647

Supplementary Table 6 (continued): D_{BBAA}-statistics for *Gadus morhua* populations and outgroups.

P1	P2	P3	C_{BBAA}	C_{ABBA}	C_{BABA}	D_{BBAA}	p
Møre (s)	Kiel Bight	Lofoten (s)	84,291.6	84,153.6	83,616.2	0.003	0.0368
Lofoten (s)	Kiel Bight	Suffolk	86,514.7	85,681.9	84,988.6	0.004	0.0018
Møre (m)	Suffolk	Bornholm Basin	84,024.6	83,775.7	83,044.5	0.004	0.0267

Supplementary Table 7: D_{fix}-statistics for *Gadus morhua* populations and outgroups.
For the calculation of the D_{fix} version of the D-statistic[27], all trios are fixed in their orientation according to an assumed tree, for which we here use the one inferred under the multi-species coalescent model (Fig. 2b). Additionally, the trio is arbitrarily oriented so that the number of "ABBA" patterns (C_{ABBA}) exceeds that of "BBAA" patterns (C_{BABA}). When the trio is oriented in this way, the D_{BBAA}-statistic is never negative and values significantly greater than zero support introgression between the two species in positions P2 and P3. If the same pair of species in positions P2 and P3 produced significant results with several third species in position P1, only the trio with the highest D_{BBAA} is listed. Significance was assessed with block-jackknifing. Sites from supergene regions were excluded from this analysis. The highest D values are underlined.

P1	P2	P3	C_{BBAA}	C_{ABBA}	C_{BABA}	D_{BBAA}	p
Lofoten (m)	*G. chalcogrammus*	*G. macrocephalus*	520,663.0	99,351.4	84,913.6	0.078	0.0000
Lofoten (m)	Newfoundland (m)	*G. macrocephalus*	1,315,150.0	13,951.5	12,893.6	0.039	0.0000
Lofoten (m)	Newfoundland (s)	*G. macrocephalus*	1,315,950.0	13,995.8	12,968.8	0.038	0.0000
Lofoten (m)	Labrador	*G. macrocephalus*	1,283,140.0	14,079.2	13,066.8	0.037	0.0000
Lofoten (m)	Møre (m)	*G. macrocephalus*	1,359,260.0	13,246.6	12,503.9	0.029	0.0000
Lofoten (m)	Møre (s)	*G. macrocephalus*	1,290,310.0	12,728.2	12,083.8	0.026	0.0000
Lofoten (m)	Iceland (m)	*G. macrocephalus*	1,360,800.0	13,236.4	12,506.8	0.028	0.0000
Lofoten (m)	Iceland (s)	*G. macrocephalus*	1,195,570.0	11,584.5	10,701.2	0.040	0.0000
Lofoten (m)	Lofoten (s)	*G. macrocephalus*	1,271,080.0	13,134.9	12,618.3	0.020	0.0000
Lofoten (m)	Suffolk	*G. macrocephalus*	1,337,050.0	13,094.1	12,316.8	0.031	0.0000
Lofoten (m)	Kiel Bight	*G. macrocephalus*	1,349,250.0	13,088.5	12,375.4	0.028	0.0000
Lofoten (m)	Bornholm Basin	*G. macrocephalus*	1,249,840.0	12,723.0	12,013.0	0.029	0.0000
G. macrocephalus	*G. ogac*	*G. chalcogrammus*	1,381,720.0	11,877.9	9,868.6	0.092	0.0000
G. macrocephalus	*G. ogac*	Newfoundland (m)	1,407,000.0	16,512.3	9,997.9	0.246	0.0000
G. macrocephalus	*G. ogac*	Newfoundland (s)	1,409,330.0	16,543.0	9,954.9	<u>0.249</u>	0.0000
G. macrocephalus	*G. ogac*	Labrador	1,362,790.0	16,024.5	9,682.8	0.247	0.0000
G. macrocephalus	*G. ogac*	Møre (m)	1,428,900.0	16,894.6	10,179.4	0.248	0.0000
G. macrocephalus	*G. ogac*	Møre (s)	1,337,080.0	15,864.3	9,578.4	0.247	0.0000
G. macrocephalus	*G. ogac*	Lofoten (m)	1,243,990.0	14,961.9	8,992.1	0.249	0.0000
G. macrocephalus	*G. ogac*	Iceland (m)	1,433,930.0	16,947.1	10,201.1	0.248	0.0000
G. macrocephalus	*G. ogac*	Iceland (s)	1,244,640.0	14,521.0	8,801.2	0.245	0.0000
G. macrocephalus	*G. ogac*	Lofoten (s)	1,306,910.0	15,591.3	9,381.0	0.249	0.0000
G. macrocephalus	*G. ogac*	Suffolk	1,399,690.0	16,515.4	9,967.1	0.247	0.0000
G. macrocephalus	*G. ogac*	Kiel Bight	1,416,600.0	16,741.8	10,039.2	<u>0.250</u>	0.0000
G. macrocephalus	*G. ogac*	Bornholm Basin	1,303,870.0	15,412.2	9,298.4	0.247	0.0000
Lofoten (m)	Newfoundland (m)	*G. chalcogrammus*	899,928.0	19,121.2	17,064.7	0.057	0.0000

Supplementary Table 7 (continued): D_{fix}-statistics for *Gadus morhua* populations and out-groups.

P1	P2	P3	C_{BBAA}	C_{ABBA}	C_{BABA}	D_{BBAA}	p
Lofoten (m)	Newfoundland (s)	*G. chalcogrammus*	900,322.0	19,129.8	17,145.6	0.055	0.0000
Lofoten (m)	Labrador	*G. chalcogrammus*	879,634.0	19,307.5	17,313.0	0.054	0.0000
Lofoten (m)	Møre (m)	*G. chalcogrammus*	940,601.0	17,992.4	16,643.0	0.039	0.0000
Lofoten (m)	Møre (s)	*G. chalcogrammus*	892,834.0	17,119.2	16,108.0	0.030	0.0000
Lofoten (m)	Iceland (m)	*G. chalcogrammus*	940,877.0	17,881.4	16,644.0	0.036	0.0000
Lofoten (m)	Iceland (s)	*G. chalcogrammus*	824,358.0	15,499.5	14,249.0	0.042	0.0000
Lofoten (m)	Lofoten (s)	*G. chalcogrammus*	880,879.0	17,736.3	16,817.5	0.027	0.0000
Lofoten (m)	Suffolk	*G. chalcogrammus*	925,054.0	17,621.4	16,512.5	0.032	0.0000
Lofoten (m)	Kiel Bight	*G. chalcogrammus*	933,388.0	17,530.1	16,578.2	0.028	0.0000
Lofoten (m)	Bornholm Basin	*G. chalcogrammus*	864,946.0	17,173.2	16,101.7	0.032	0.0000
Lofoten (m)	Møre (m)	Newfoundland (m)	101,116.0	76,042.8	73,359.0	0.018	0.0000
Lofoten (m)	Møre (s)	Newfoundland (m)	97,687.3	73,463.8	71,353.9	0.015	0.0000
Newfoundland (s)	Newfoundland (m)	Lofoten (m)	100,337.0	73,708.3	73,166.3	0.004	0.0199
Lofoten (m)	Iceland (m)	Newfoundland (m)	99,598.9	76,880.4	73,436.2	0.023	0.0000
Lofoten (m)	Iceland (s)	Newfoundland (m)	87,416.8	67,254.8	64,090.8	0.024	0.0000
Lofoten (m)	Lofoten (s)	Newfoundland (m)	99,879.5	75,403.2	73,398.0	0.013	0.0000
Lofoten (m)	Suffolk	Newfoundland (m)	99,806.2	75,106.4	72,659.9	0.017	0.0000
Lofoten (m)	Kiel Bight	Newfoundland (m)	100,081.0	75,274.3	72,882.2	0.016	0.0000
Lofoten (m)	Bornholm Basin	Newfoundland (m)	95,876.1	72,700.8	71,137.0	0.011	0.0000
Lofoten (m)	Møre (m)	Newfoundland (s)	101,717.0	76,179.5	73,376.7	0.019	0.0000
Lofoten (m)	Møre (s)	Newfoundland (s)	98,290.6	73,381.3	71,418.9	0.014	0.0000
Lofoten (m)	Iceland (m)	Newfoundland (s)	100,066.0	77,109.0	73,368.2	0.025	0.0000
Lofoten (m)	Iceland (s)	Newfoundland (s)	87,938.8	67,313.5	64,024.0	0.025	0.0000
Lofoten (m)	Lofoten (s)	Newfoundland (s)	100,514.0	75,274.5	73,461.6	0.012	0.0000
Lofoten (m)	Suffolk	Newfoundland (s)	100,406.0	75,163.5	72,707.5	0.017	0.0000
Lofoten (m)	Kiel Bight	Newfoundland (s)	100,678.0	75,348.4	72,961.2	0.016	0.0000
Lofoten (m)	Bornholm Basin	Newfoundland (s)	96,373.6	72,764.8	71,170.7	0.011	0.0000
Lofoten (m)	Møre (m)	Labrador	101,470.0	76,990.3	73,939.4	0.020	0.0000
Lofoten (m)	Møre (s)	Labrador	98,044.0	74,081.3	71,929.0	0.015	0.0000
Newfoundland (s)	Labrador	Lofoten (m)	99,742.5	75,063.6	74,447.7	0.004	0.0279
Lofoten (m)	Iceland (m)	Labrador	99,959.3	77,749.7	73,957.8	0.025	0.0000
Lofoten (m)	Iceland (s)	Labrador	87,825.1	67,579.0	64,608.9	0.022	0.0000
Lofoten (m)	Lofoten (s)	Labrador	100,116.0	76,114.1	73,845.5	0.015	0.0000
Lofoten (m)	Suffolk	Labrador	100,325.0	75,694.3	73,373.0	0.016	0.0000
Lofoten (m)	Kiel Bight	Labrador	100,604.0	75,824.4	73,680.3	0.014	0.0000
Lofoten (m)	Bornholm Basin	Labrador	96,187.9	73,617.0	71,666.0	0.013	0.0000
Lofoten (m)	Møre (s)	Møre (m)	80,571.3	84,048.2	80,947.2	0.019	0.0000
Lofoten (m)	Iceland (m)	Møre (m)	82,604.6	86,643.9	84,266.6	0.014	0.0000
Lofoten (m)	Iceland (s)	Møre (m)	72,001.0	74,983.3	73,196.2	0.012	0.0000
Lofoten (m)	Lofoten (s)	Møre (m)	83,276.1	86,092.4	83,367.1	0.016	0.0000
Lofoten (m)	Suffolk	Møre (m)	82,333.2	86,034.3	82,638.9	0.020	0.0000
Lofoten (m)	Kiel Bight	Møre (m)	82,562.5	86,296.7	82,997.0	0.019	0.0000
Lofoten (m)	Bornholm Basin	Møre (m)	79,469.0	83,254.0	80,923.7	0.014	0.0000
Lofoten (m)	Møre (s)	Iceland (m)	81,388.9	83,186.0	80,164.7	0.018	0.0000
Lofoten (m)	Møre (s)	Iceland (s)	71,381.1	72,775.3	70,473.5	0.016	0.0000
Lofoten (m)	Møre (s)	Lofoten (s)	80,085.8	84,303.2	80,326.7	0.024	0.0000
Lofoten (m)	Møre (s)	Suffolk	79,554.5	83,557.4	79,595.2	0.024	0.0000

Supplementary Table 7 (continued): D_{fix}-statistics for *Gadus morhua* populations and outgroups.

P1	P2	P3	C_{BBAA}	C_{ABBA}	C_{BABA}	D_{BBAA}	p
Lofoten (m)	Møre (s)	Kiel Bight	79,506.0	84,530.7	79,462.2	0.031	0.0000
Lofoten (m)	Møre (s)	Bornholm Basin	77,833.7	81,360.8	76,767.9	0.029	0.0000
Iceland (m)	Lofoten (s)	Lofoten (m)	84,955.3	84,141.1	82,642.6	0.009	0.0000
Iceland (m)	Suffolk	Lofoten (m)	85,394.6	83,070.4	81,713.4	0.008	0.0000
Iceland (m)	Kiel Bight	Lofoten (m)	85,257.8	83,440.5	82,256.6	0.007	0.0000
Lofoten (s)	Suffolk	Iceland (m)	87,585.4	86,223.8	84,759.5	0.009	0.0000
Bornholm Basin	Kiel Bight	Iceland (m)	90,081.5	81,472.2	80,596.6	0.005	0.0017
Bornholm Basin	Lofoten (s)	Iceland (s)	75,788.9	72,473.6	71,901.6	0.004	0.0698
Bornholm Basin	Suffolk	Iceland (s)	73,774.8	72,801.1	71,399.4	0.010	0.0001
Bornholm Basin	Kiel Bight	Iceland (s)	78,755.6	71,169.0	70,455.3	0.005	0.0647
Suffolk	Lofoten (s)	Kiel Bight	84,988.6	86,514.7	85,681.9	0.005	0.0002
Suffolk	Lofoten (s)	Bornholm Basin	82,724.6	84,150.7	82,438.2	0.010	0.0000
Bornholm Basin	Kiel Bight	Suffolk	87,465.9	81,655.5	80,793.0	0.005	0.0022

Supplementary Table 8: D_{BBAA}-statistics for *Gadus morhua* populations and outgroups within the supergene region on LG 1.
The D_{BBAA} version of the D-statistic[27] was calculated as for Supplementary Table 6, except that only sites from the supergene region on LG 1 were used. The highest D values are underlined.

P1	P2	P3	C_{BBAA}	C_{ABBA}	C_{BABA}	D_{BBAA}	p
Newfoundland (s)	*G. chalcogrammus*	*G. macrocephalus*	18,487.3	3,724.6	3,100.6	0.091	0.0000
Iceland (m)	Newfoundland (m)	*G. macrocephalus*	49,542.8	212.7	185.5	0.068	0.0050
Kiel Bight	Lofoten (s)	*G. macrocephalus*	48,224.6	316.9	290.4	0.044	0.0169
Kiel Bight	Suffolk	*G. macrocephalus*	51,567.5	315.3	290.9	0.040	0.0312
Kiel Bight	Møre (s)	*G. macrocephalus*	49,294.1	315.4	283.0	0.054	0.0092
Newfoundland (s)	*G. chalcogrammus*	*G. ogac*	16,967.9	3,449.0	3,009.4	0.068	0.0000
G. macrocephalus	*G. ogac*	Newfoundland (m)	44,845.4	512.2	338.7	0.204	0.0000
G. macrocephalus	*G. ogac*	Iceland (m)	45,983.8	534.5	348.5	0.211	0.0000
G. macrocephalus	*G. ogac*	Møre (m)	45,841.7	537.7	345.2	0.218	0.0000
G. macrocephalus	*G. ogac*	Lofoten (m)	39,458.0	457.2	292.6	0.220	0.0000
G. macrocephalus	*G. ogac*	Newfoundland (s)	44,555.7	466.5	334.2	0.165	0.0000
G. macrocephalus	*G. ogac*	Labrador	42,906.4	460.1	324.2	0.173	0.0000
G. macrocephalus	*G. ogac*	Iceland (s)	38,876.5	410.5	298.0	0.159	0.0000
G. macrocephalus	*G. ogac*	Lofoten (s)	40,723.6	439.4	317.3	0.161	0.0000
G. macrocephalus	*G. ogac*	Suffolk	44,190.2	464.3	342.1	0.152	0.0000
G. macrocephalus	*G. ogac*	Kiel Bight	44,891.2	472.6	346.4	0.154	0.0000
G. macrocephalus	*G. ogac*	Møre (s)	41,842.6	446.7	323.9	0.159	0.0000
G. macrocephalus	*G. ogac*	Bornholm Basin	41,037.4	434.5	318.9	0.153	0.0000
Lofoten (m)	Newfoundland (m)	*G. chalcogrammus*	30,070.9	261.1	232.4	0.058	0.0153
Lofoten (m)	Iceland (m)	*G. chalcogrammus*	33,208.8	195.6	176.3	0.052	0.0145
Møre (m)	Newfoundland (s)	*G. chalcogrammus*	19,552.6	2,027.7	1,886.4	0.036	0.0224
Møre (m)	Labrador	*G. chalcogrammus*	18,468.8	2,112.1	1,945.4	0.041	0.0260
Bornholm Basin	Iceland (s)	*G. chalcogrammus*	29,160.5	392.2	341.5	0.069	0.0117
Møre (m)	Lofoten (s)	*G. chalcogrammus*	17,429.4	2,049.7	1,928.4	0.031	0.0755
Møre (m)	Suffolk	*G. chalcogrammus*	18,897.4	2,137.4	1,989.1	0.036	0.0361
Møre (m)	Kiel Bight	*G. chalcogrammus*	19,207.2	2,176.5	2,019.2	0.037	0.0194
Bornholm Basin	Møre (s)	*G. chalcogrammus*	33,024.7	367.6	330.6	0.053	0.0139
Møre (m)	Bornholm Basin	*G. chalcogrammus*	17,629.4	2,016.5	1,912.0	0.027	0.0908
Møre (m)	Iceland (m)	Newfoundland (m)	2,630.7	1,295.3	1,199.3	0.038	0.0514
Lofoten (m)	Newfoundland (m)	Newfoundland (s)	14,465.7	1,040.8	464.5	0.383	0.0000
Iceland (m)	Newfoundland (m)	Labrador	16,935.5	1,152.4	461.4	<u>0.428</u>	0.0000
Iceland (m)	Newfoundland (m)	Iceland (s)	15,160.8	1,002.5	414.4	0.415	0.0000
Iceland (m)	Newfoundland (m)	Lofoten (s)	16,682.0	1,048.9	470.8	0.380	0.0000
Lofoten (m)	Newfoundland (m)	Suffolk	15,213.9	999.9	461.1	0.369	0.0000
Iceland (m)	Newfoundland (m)	Kiel Bight	17,605.9	1,105.1	505.8	0.372	0.0000
Lofoten (m)	Newfoundland (m)	Møre (s)	14,781.6	969.4	443.4	0.372	0.0000
Iceland (m)	Newfoundland (m)	Bornholm Basin	16,491.6	1,040.0	476.6	0.372	0.0000
Lofoten (m)	Iceland (m)	Møre (m)	1,423.2	1422.9	1,368.3	0.020	0.0832
Labrador	Newfoundland (s)	Iceland (m)	19,409.3	1280.1	711.1	0.286	0.0000
Bornholm Basin	Labrador	Iceland (m)	17,553.5	926.0	842.9	0.047	0.0695
Møre (s)	Iceland (s)	Iceland (m)	16,791.3	728.4	668.8	0.043	0.0583
Møre (s)	Suffolk	Iceland (m)	20,436.4	706.2	677.3	0.021	0.0679
Møre (s)	Kiel Bight	Iceland (m)	20,578.5	713.7	660.1	0.039	0.0066
Labrador	Newfoundland (s)	Møre (m)	19,308.6	1,279.2	730.8	0.273	0.0000

Supplementary Table 8 (continued): D_{BBAA}-statistics for *Gadus morhua* populations and outgroups within the supergene region on LG 1.

P1	P2	P3	C_{BBAA}	C_{ABBA}	C_{BABA}	D_{BBAA}	p
Lofoten (m)	Møre (m)	Labrador	17,641.5	535.2	426.9	0.113	0.0016
Lofoten (m)	Møre (m)	Iceland (s)	15,738.5	482.1	381.2	0.117	0.0026
Iceland (m)	Møre (m)	Lofoten (s)	19,879.4	557.8	451.5	0.105	0.0050
Lofoten (m)	Møre (m)	Suffolk	18,093.3	568.8	461.0	0.105	0.0034
Lofoten (m)	Møre (m)	Kiel Bight	18,248.7	581.2	455.5	0.121	0.0008
Lofoten (m)	Møre (m)	Møre (s)	17,509.5	554.2	441.1	0.114	0.0024
Lofoten (m)	Møre (m)	Bornholm Basin	17,149.7	531.9	427.6	0.109	0.0039
Labrador	Newfoundland (s)	Lofoten (m)	16,820.0	1,103.8	612.9	0.286	0.0000
Møre (s)	Iceland (s)	Lofoten (m)	14,644.3	640.7	577.0	0.052	0.0463
Møre (s)	Suffolk	Lofoten (m)	17,679.3	620.2	593.3	0.022	0.0902
Møre (s)	Kiel Bight	Lofoten (m)	17,772.6	629.1	568.9	0.050	0.0018
Suffolk	Iceland (s)	Newfoundland (s)	3,077.7	2,619.6	1,718.4	0.208	0.0000
Newfoundland (s)	Labrador	Iceland (s)	2,715.7	2,432.7	1,833.5	0.140	0.0000
Newfoundland (s)	Labrador	Lofoten (s)	3,801.3	2,357.9	1,949.4	0.095	0.0000
Newfoundland (s)	Labrador	Suffolk	3,993.3	2,425.8	1,961.8	0.106	0.0000
Newfoundland (s)	Labrador	Kiel Bight	4,008.5	2,415.2	1,981.8	0.099	0.0000
Newfoundland (s)	Labrador	Møre (s)	3,857.1	2,357.5	1,926.3	0.101	0.0000
Newfoundland (s)	Labrador	Bornholm Basin	3,739.3	2,312.7	1,873.5	0.105	0.0000
Labrador	Iceland (s)	Lofoten (s)	2,706.1	2,585.8	1,673.9	0.214	0.0000
Labrador	Iceland (s)	Suffolk	2,786.5	2,606.7	1,699.8	0.211	0.0000
Labrador	Iceland (s)	Kiel Bight	2,812.2	2,573.9	1,691.7	0.207	0.0000
Labrador	Iceland (s)	Møre (s)	2,719.0	2,529.8	1,643.2	0.212	0.0000
Labrador	Iceland (s)	Bornholm Basin	2,642.8	2,440.4	1,643.7	0.195	0.0000
Bornholm Basin	Lofoten (s)	Suffolk	2,372.5	2,362.2	2,279.9	0.018	0.0460
Bornholm Basin	Møre (s)	Suffolk	2,324.7	2,322.5	2,239.2	0.018	0.0311

Supplementary Table 9: D_{fix}-statistics for *Gadus morhua* populations and outgroups within the supergene region on LG 1.
The D_{fix} version of the D-statistic[27] was calculated as for Supplementary Table 6, except that only sites from the supergene region on LG 1 were used and the input tree was the one inferred for the supergene on LG 1 under the multi-species coalescent model (Fig. 3a).

P1	P2	P3	C_{BBAA}	C_{ABBA}	C_{BABA}	D_{BBAA}	p
Newfoundland (s)	*G. chalcogrammus*	*G. macrocephalus*	18,487.3	3,724.6	3,100.6	0.091	0.0000
Iceland (m)	Newfoundland (m)	*G. macrocephalus*	49,542.8	212.7	185.5	0.068	0.0050
Kiel Bight	Lofoten (s)	*G. macrocephalus*	48,224.6	316.9	290.4	0.044	0.0169
Kiel Bight	Suffolk	*G. macrocephalus*	51,567.5	315.3	290.9	0.040	0.0312
Kiel Bight	Møre (s)	*G. macrocephalus*	49,294.1	315.4	283.0	0.054	0.0092
Newfoundland (s)	*G. chalcogrammus*	*G. ogac*	16,967.9	3,449.0	3,009.4	0.068	0.0000
G. macrocephalus	*G. ogac*	Newfoundland (m)	44,845.4	512.2	338.7	0.204	0.0000
G. macrocephalus	*G. ogac*	Iceland (m)	45,983.8	534.5	348.5	0.211	0.0000
G. macrocephalus	*G. ogac*	Møre (m)	45,841.7	537.7	345.2	0.218	0.0000

Supplementary Table 9 (continued): D_{fix}-statistics for *Gadus morhua* populations and outgroups within the supergene region on LG 1.

P1	P2	P3	C_{BBAA}	C_{ABBA}	C_{BABA}	D_{BBAA}	p
G. macrocephalus	*G. ogac*	Lofoten (m)	39,458.0	457.2	292.6	0.220	0.0000
G. macrocephalus	*G. ogac*	Newfoundland (s)	44,555.7	466.5	334.2	0.165	0.0000
G. macrocephalus	*G. ogac*	Labrador	42,906.4	460.1	324.2	0.173	0.0000
G. macrocephalus	*G. ogac*	Iceland (s)	38,876.5	410.5	298.0	0.159	0.0000
G. macrocephalus	*G. ogac*	Lofoten (s)	40,723.6	439.4	317.3	0.161	0.0000
G. macrocephalus	*G. ogac*	Suffolk	44,190.2	464.3	342.1	0.152	0.0000
G. macrocephalus	*G. ogac*	Kiel Bight	44,891.2	472.6	346.4	0.154	0.0000
G. macrocephalus	*G. ogac*	Møre (s)	41,842.6	446.7	323.9	0.159	0.0000
G. macrocephalus	*G. ogac*	Bornholm Basin	41,037.4	434.5	318.9	0.153	0.0000
Lofoten (m)	Newfoundland (m)	*G. chalcogrammus*	30,070.9	261.1	232.4	0.058	0.0153
Lofoten (m)	Iceland (m)	*G. chalcogrammus*	33,208.8	195.6	176.3	0.052	0.0145
Møre (m)	Newfoundland (s)	*G. chalcogrammus*	19,552.6	2,027.7	1,886.4	0.036	0.0224
Møre (m)	Labrador	*G. chalcogrammus*	18,468.8	2,112.1	1,945.4	0.041	0.0260
Bornholm Basin	Iceland (s)	*G. chalcogrammus*	29,160.5	392.2	341.5	0.069	0.0117
Møre (m)	Lofoten (s)	*G. chalcogrammus*	17,429.4	2,049.7	1,928.4	0.031	0.0755
Møre (m)	Suffolk	*G. chalcogrammus*	18,897.4	2,137.4	1,989.1	0.036	0.0361
Møre (m)	Kiel Bight	*G. chalcogrammus*	19,207.2	2,176.5	2,019.2	0.037	0.0194
Bornholm Basin	Møre (s)	*G. chalcogrammus*	33,024.7	367.6	330.6	0.053	0.0139
Møre (m)	Bornholm Basin	*G. chalcogrammus*	17,629.4	2,016.5	1,912.0	0.027	0.0908
Møre (m)	Iceland (m)	Newfoundland (m)	2,630.7	1,295.3	1,199.3	0.038	0.0514
Lofoten (m)	Newfoundland (m)	Newfoundland (s)	14,465.7	1,040.8	464.5	0.383	0.0000
Iceland (m)	Newfoundland (m)	Labrador	16,935.5	1,152.4	461.4	<u>0.428</u>	0.0000
Iceland (m)	Newfoundland (m)	Iceland (s)	15,160.8	1,002.5	414.4	0.415	0.0000
Iceland (m)	Newfoundland (m)	Lofoten (s)	16,682.0	1,048.9	470.8	0.380	0.0000
Lofoten (m)	Newfoundland (m)	Suffolk	15,213.9	999.9	461.1	0.369	0.0000
Iceland (m)	Newfoundland (m)	Kiel Bight	17,605.9	1,105.1	505.8	0.372	0.0000
Lofoten (m)	Newfoundland (m)	Møre (s)	14,781.6	969.4	443.4	0.372	0.0000
Iceland (m)	Newfoundland (m)	Bornholm Basin	16,491.6	1,040.0	476.6	0.372	0.0000
Labrador	Newfoundland (s)	Iceland (m)	19,409.3	1,280.1	711.1	0.286	0.0000
Bornholm Basin	Labrador	Iceland (m)	17,553.5	926.0	842.9	0.047	0.0695
Møre (s)	Iceland (s)	Iceland (m)	16,791.3	728.4	668.8	0.043	0.0583
Møre (s)	Suffolk	Iceland (m)	20,436.4	706.2	677.3	0.021	0.0679
Møre (s)	Kiel Bight	Iceland (m)	20,578.5	713.7	660.1	0.039	0.0066
Labrador	Newfoundland (s)	Møre (m)	19,308.6	1,279.2	730.8	0.273	0.0000
Lofoten (m)	Møre (m)	Labrador	17,641.5	535.2	426.9	0.113	0.0016
Lofoten (m)	Møre (m)	Iceland (s)	15,738.5	482.1	381.2	0.117	0.0026
Iceland (m)	Møre (m)	Lofoten (s)	19,879.4	557.8	451.5	0.105	0.0050
Lofoten (m)	Møre (m)	Suffolk	18,093.3	568.8	461.0	0.105	0.0034
Lofoten (m)	Møre (m)	Kiel Bight	18,248.7	581.2	455.5	0.121	0.0008
Lofoten (m)	Møre (m)	Møre (s)	17,509.5	554.2	441.1	0.114	0.0024
Lofoten (m)	Møre (m)	Bornholm Basin	17,149.7	531.9	427.6	0.109	0.0039
Labrador	Newfoundland (s)	Lofoten (m)	16,820.0	1,103.8	612.9	0.286	0.0000
Møre (s)	Iceland (s)	Lofoten (m)	14,644.3	640.7	577.0	0.052	0.0463
Møre (s)	Suffolk	Lofoten (m)	17,679.3	620.2	593.3	0.022	0.0902
Møre (s)	Kiel Bight	Lofoten (m)	17,772.6	629.1	568.9	0.050	0.0018
Suffolk	Iceland (s)	Newfoundland (s)	3,077.7	2,619.6	1,718.4	0.208	0.0000
Kiel Bight	Iceland (s)	Labrador	2,573.9	2,812.2	1,691.7	0.249	0.0000

Supplementary Table 9 (continued): D_{fix}-statistics for *Gadus morhua* populations and outgroups within the supergene region on LG 1.

P1	P2	P3	C_{BBAA}	C_{ABBA}	C_{BABA}	D_{BBAA}	p
Newfoundland (s)	Labrador	Lofoten (s)	3,801.3	2,357.9	1,949.4	0.095	0.0000
Newfoundland (s)	Labrador	Suffolk	3,993.3	2,425.8	1,961.8	0.106	0.0000
Newfoundland (s)	Labrador	Kiel Bight	4,008.5	2,415.2	1,981.8	0.099	0.0000
Newfoundland (s)	Labrador	Møre (s)	3,857.1	2,357.5	1,926.3	0.101	0.0000
Newfoundland (s)	Labrador	Bornholm Basin	3,739.3	2,312.7	1,873.5	0.105	0.0000
Bornholm Basin	Lofoten (s)	Iceland (s)	2,931.2	1,839.6	1,739.2	0.028	0.0090
Bornholm Basin	Suffolk	Iceland (s)	2,877.7	1,835.4	1,749.3	0.024	0.0182
Bornholm Basin	Møre (s)	Suffolk	2,324.7	2,322.5	2,239.2	0.018	0.0311

Supplementary Table 10: D_{BBAA}-statistics for *Gadus morhua* populations and outgroups within the supergene region on LG 2.
The D_{BBAA} version of the D-statistic[27] was calculated as for Supplementary Table 6, except that only sites from the supergene region on LG 2 were used. The highest D values are underlined.

P1	P2	P3	C_{BBAA}	C_{ABBA}	C_{BABA}	D_{BBAA}	p
Labrador	*G. chalcogrammus*	*G. macrocephalus*	4,523.6	1,220.8	941.6	0.129	0.0000
Iceland (m)	Møre (s)	*G. macrocephalus*	8,549.2	451.4	329.6	0.156	0.0002
Bornholm Basin	Suffolk	*G. macrocephalus*	8,449.9	428.6	326.2	0.136	0.0002
Bornholm Basin	Kiel Bight	*G. macrocephalus*	8,583.7	444.1	324.8	0.155	0.0000
Bornholm Basin	Lofoten (s)	*G. macrocephalus*	7,876.1	415.2	320.4	0.129	0.0003
Iceland (m)	Newfoundland (m)	*G. macrocephalus*	12,739.7	127.8	108.2	0.083	0.0325
Lofoten (m)	Labrador	*G. macrocephalus*	10,727.8	113.8	101.1	0.059	0.0910
Lofoten (m)	Bornholm Basin	*G. macrocephalus*	10,515.5	110.4	96.6	0.066	0.0752
Lofoten (m)	Møre (m)	*G. macrocephalus*	11,448.6	115.5	105.5	0.045	0.0714
Labrador	*G. chalcogrammus*	*G. ogac*	4,161.8	1,162.4	926.9	0.113	0.0000
G. macrocephalus	*G. ogac*	Møre (s)	11,666.4	157.8	84.2	0.304	0.0000
G. macrocephalus	*G. ogac*	Suffolk	12,617.5	169.2	94.1	0.285	0.0000
G. macrocephalus	*G. ogac*	Kiel Bight	12,868.9	169.2	95.3	0.279	0.0000
G. macrocephalus	*G. ogac*	Lofoten (s)	11,722.3	156.2	86.2	0.289	0.0000
G. macrocephalus	*G. ogac*	Newfoundland (s)	13,040.0	179.1	96.6	0.299	0.0000
G. macrocephalus	*G. ogac*	Newfoundland (m)	12,934.5	177.2	95.8	0.299	0.0000
G. macrocephalus	*G. ogac*	Labrador	12,633.0	173.8	93.1	0.302	0.0000
G. macrocephalus	*G. ogac*	Iceland (m)	13,348.1	188.5	98.5	0.314	0.0000
G. macrocephalus	*G. ogac*	Lofoten (m)	11,184.0	160.1	84.8	0.308	0.0000
G. macrocephalus	*G. ogac*	Bornholm Basin	12,146.6	170.1	90.4	0.306	0.0000
G. macrocephalus	*G. ogac*	Iceland (s)	11,465.5	162.2	81.2	<u>0.333</u>	0.0000
G. macrocephalus	*G. ogac*	Møre (m)	13,275.2	183.5	98.0	0.304	0.0000
Lofoten (m)	Møre (s)	*G. chalcogrammus*	4,646.8	563.8	482.2	0.078	0.0275
Iceland (s)	Suffolk	*G. chalcogrammus*	5,020.7	586.2	503.1	0.076	0.0242
Lofoten (m)	Kiel Bight	*G. chalcogrammus*	5,025.2	615.6	516.2	0.088	0.0095
Lofoten (m)	Lofoten (s)	*G. chalcogrammus*	4,676.1	578.6	498.8	0.074	0.0216
Iceland (s)	Newfoundland (m)	*G. chalcogrammus*	8,021.9	129.8	118.1	0.047	0.0710

Supplementary Table 10 (continued): D_{BBAA}-statistics for *Gadus morhua* populations and outgroups within the supergene region on LG 2.

P1	P2	P3	C_{BBAA}	C_{ABBA}	C_{BABA}	D_{BBAA}	p
Iceland (s)	Labrador	*G. chalcogrammus*	7,841.8	132.3	112.9	0.079	0.0335
Iceland (s)	Iceland (m)	*G. chalcogrammus*	8,316.7	138.8	122.4	0.063	0.0526
Lofoten (m)	Bornholm Basin	*G. chalcogrammus*	7,701.9	152.3	109.8	0.162	0.0003
Lofoten (m)	Møre (m)	*G. chalcogrammus*	8,400.6	155.4	136.2	0.066	0.0159
Suffolk	Møre (s)	Newfoundland (s)	5,848.9	232.1	204.8	0.062	0.0179
Suffolk	Møre (s)	Newfoundland (m)	5,806.1	225.4	196.7	0.068	0.0125
Suffolk	Møre (s)	Labrador	5,678.6	227.7	202.2	0.059	0.0101
Newfoundland (s)	Iceland (m)	Møre (s)	3,884.3	380.7	296.8	0.124	0.0068
Newfoundland (s)	Lofoten (m)	Møre (s)	3,425.5	334.6	288.7	0.074	0.0423
Newfoundland (s)	Bornholm Basin	Møre (s)	3,612.4	372.1	260.2	0.177	0.0001
Newfoundland (s)	Iceland (s)	Møre (s)	3,433.4	323.3	254.2	0.120	0.0034
Newfoundland (s)	Møre (m)	Møre (s)	3,853.4	378.1	296.7	0.121	0.0058
Newfoundland (s)	Newfoundland (m)	Suffolk	4,180.7	298.6	268.8	0.053	0.0214
Newfoundland (s)	Labrador	Suffolk	4,082.7	296.8	275.3	0.038	0.0891
Newfoundland (s)	Iceland (m)	Suffolk	4,101.5	404.2	303.7	0.142	0.0032
Newfoundland (s)	Lofoten (m)	Suffolk	3,596.5	355.6	301.9	0.082	0.0245
Newfoundland (s)	Bornholm Basin	Suffolk	3,801.8	389.0	267.9	0.184	0.0001
Newfoundland (s)	Iceland (s)	Suffolk	3,576.3	342.8	247.8	0.161	0.0001
Newfoundland (s)	Møre (m)	Suffolk	4,072.1	399.8	307.8	0.130	0.0042
Newfoundland (s)	Newfoundland (m)	Kiel Bight	4,228.3	308.4	278.4	0.051	0.0353
Newfoundland (s)	Labrador	Kiel Bight	4,130.4	313.1	284.5	0.048	0.0419
Newfoundland (s)	Iceland (m)	Kiel Bight	4,146.2	416.1	314.0	0.140	0.0043
Newfoundland (s)	Lofoten (m)	Kiel Bight	3,645.5	357.0	310.0	0.071	0.0542
Newfoundland (s)	Bornholm Basin	Kiel Bight	3,833.9	406.4	279.6	0.185	0.0001
Newfoundland (s)	Iceland (s)	Kiel Bight	3,624.7	348.2	256.6	0.151	0.0005
Newfoundland (s)	Møre (m)	Kiel Bight	4,115.3	412.1	317.6	0.130	0.0031
Newfoundland (s)	Labrador	Lofoten (s)	3,914.1	285.2	261.1	0.044	0.0461
Newfoundland (s)	Iceland (m)	Lofoten (s)	3,933.1	381.0	290.6	0.135	0.0049
Newfoundland (s)	Lofoten (m)	Lofoten (s)	3,471.0	339.4	291.5	0.076	0.0446
Newfoundland (s)	Bornholm Basin	Lofoten (s)	3,649.7	383.3	258.6	0.194	0.0001
Newfoundland (s)	Iceland (s)	Lofoten (s)	3,477.8	324.7	238.8	0.152	0.0002
Newfoundland (s)	Møre (m)	Lofoten (s)	3,909.0	379.8	294.3	0.127	0.0045
Møre (m)	Iceland (m)	Newfoundland (s)	891.3	625.1	588.6	0.030	0.0532
Møre (m)	Bornholm Basin	Newfoundland (s)	856.1	574.5	520.8	0.049	0.0155
Møre (m)	Iceland (s)	Newfoundland (s)	776.0	546.4	471.5	0.074	0.0051
Møre (m)	Iceland (m)	Newfoundland (m)	882.2	636.2	588.7	0.039	0.0303
Møre (m)	Bornholm Basin	Newfoundland (m)	846.9	577.2	520.6	0.052	0.0203
Møre (m)	Iceland (s)	Newfoundland (m)	767.2	560.9	467.3	0.091	0.0000
Newfoundland (s)	Labrador	Iceland (m)	793.8	661.6	600.1	0.049	0.0070
Newfoundland (s)	Labrador	Lofoten (m)	761.2	602.8	559.8	0.037	0.0037
Newfoundland (s)	Labrador	Bornholm Basin	737.1	627.0	572.6	0.045	0.0353
Newfoundland (s)	Labrador	Iceland (s)	648.7	575.6	521.9	0.049	0.0119
Newfoundland (s)	Labrador	Møre (m)	819.9	652.1	588.0	0.052	0.0029
Lofoten (m)	Bornholm Basin	Iceland (m)	659.5	637.8	579.9	0.048	0.0194
Møre (m)	Bornholm Basin	Lofoten (m)	639.9	630.7	592.8	0.031	0.0870

Supplementary Table 11: D_{fix}-statistics for *Gadus morhua* populations and outgroups within the supergene region on LG 2.

The D_{fix} version of the D-statistic[27] was calculated as for Supplementary Table 6, except that only sites from the supergene region on LG 2 were used and the input tree was the one inferred for the supergene on LG 2 under the multi-species coalescent model (Fig. 3d). The highest D values are underlined.

P1	P2	P3	C_{BBAA}	C_{ABBA}	C_{BABA}	D_{BBAA}	p
Labrador	*G. chalcogrammus*	*G. macrocephalus*	4,523.6	1,220.8	941.6	0.129	0.0000
Iceland (m)	Møre (s)	*G. macrocephalus*	8,549.2	451.4	329.6	0.156	0.0002
Bornholm Basin	Suffolk	*G. macrocephalus*	8,449.9	428.6	326.2	0.136	0.0002
Bornholm Basin	Kiel Bight	*G. macrocephalus*	8,583.7	444.1	324.8	0.155	0.0000
Bornholm Basin	Lofoten (s)	*G. macrocephalus*	7,876.1	415.2	320.4	0.129	0.0003
Iceland (m)	Newfoundland (m)	*G. macrocephalus*	12,739.7	127.8	108.2	0.083	0.0325
Lofoten (m)	Labrador	*G. macrocephalus*	10,727.8	113.8	101.1	0.059	0.0910
Lofoten (m)	Bornholm Basin	*G. macrocephalus*	10,515.5	110.4	96.6	0.066	0.0752
Lofoten (m)	Møre (m)	*G. macrocephalus*	11,448.6	115.5	105.5	0.045	0.0714
Labrador	*G. chalcogrammus*	*G. ogac*	4,161.8	1,162.4	926.9	0.113	0.0000
G. macrocephalus	*G. ogac*	Møre (s)	11,666.4	157.8	84.2	0.304	0.0000
G. macrocephalus	*G. ogac*	Suffolk	12,617.5	169.2	94.1	0.285	0.0000
G. macrocephalus	*G. ogac*	Kiel Bight	12,868.9	169.2	95.3	0.279	0.0000
G. macrocephalus	*G. ogac*	Lofoten (s)	11,722.3	156.2	86.2	0.289	0.0000
G. macrocephalus	*G. ogac*	Newfoundland (s)	13,040.0	179.1	96.6	0.299	0.0000
G. macrocephalus	*G. ogac*	Newfoundland (m)	12,934.5	177.2	95.8	0.299	0.0000
G. macrocephalus	*G. ogac*	Labrador	12,633.0	173.8	93.1	0.302	0.0000
G. macrocephalus	*G. ogac*	Iceland (m)	13,348.1	188.5	98.5	0.314	0.0000
G. macrocephalus	*G. ogac*	Lofoten (m)	11,184.0	160.1	84.8	0.308	0.0000
G. macrocephalus	*G. ogac*	Bornholm Basin	12,146.6	170.1	90.4	0.306	0.0000
G. macrocephalus	*G. ogac*	Iceland (s)	11,465.5	162.2	81.2	<u>0.333</u>	0.0000
G. macrocephalus	*G. ogac*	Møre (m)	13,275.2	183.5	98.0	0.304	0.0000
Lofoten (m)	Møre (s)	*G. chalcogrammus*	4,646.8	563.8	482.2	0.078	0.0275
Iceland (s)	Suffolk	*G. chalcogrammus*	5,020.7	586.2	503.1	0.076	0.0242
Lofoten (m)	Kiel Bight	*G. chalcogrammus*	5,025.2	615.6	516.2	0.088	0.0095
Lofoten (m)	Lofoten (s)	*G. chalcogrammus*	4,676.1	578.6	498.8	0.074	0.0216
Iceland (s)	Newfoundland (m)	*G. chalcogrammus*	8,021.9	129.8	118.1	0.047	0.0710
Iceland (s)	Labrador	*G. chalcogrammus*	7,841.8	132.3	112.9	0.079	0.0335
Iceland (s)	Iceland (m)	*G. chalcogrammus*	8,316.7	138.8	122.4	0.063	0.0526
Lofoten (m)	Bornholm Basin	*G. chalcogrammus*	7,701.9	152.3	109.8	0.162	0.0003
Lofoten (m)	Møre (m)	*G. chalcogrammus*	8,400.6	155.4	136.2	0.066	0.0159
Suffolk	Møre (s)	Newfoundland (s)	5,848.9	232.1	204.8	0.062	0.0179
Suffolk	Møre (s)	Newfoundland (m)	5,806.1	225.4	196.7	0.068	0.0125
Suffolk	Møre (s)	Labrador	5,678.6	227.7	202.2	0.059	0.0101
Newfoundland (s)	Iceland (m)	Møre (s)	3,884.3	380.7	296.8	0.124	0.0068
Newfoundland (s)	Lofoten (m)	Møre (s)	3,425.5	334.6	288.7	0.074	0.0423
Newfoundland (s)	Bornholm Basin	Møre (s)	3,612.4	372.1	260.2	0.177	0.0001
Newfoundland (s)	Iceland (s)	Møre (s)	3,433.4	323.3	254.2	0.120	0.0034
Newfoundland (s)	Møre (m)	Møre (s)	3,853.4	378.1	296.7	0.121	0.0058
Newfoundland (s)	Newfoundland (m)	Suffolk	4,180.7	298.6	268.8	0.053	0.0214
Newfoundland (s)	Labrador	Suffolk	4,082.7	296.8	275.3	0.038	0.0891
Newfoundland (s)	Iceland (m)	Suffolk	4,101.5	404.2	303.7	0.142	0.0032

Supplementary Table 11 (continued): D_{fix}-statistics for *Gadus morhua* populations and outgroups within the supergene region on LG 2.

P1	P2	P3	C_{BBAA}	C_{ABBA}	C_{BABA}	D_{BBAA}	p
Newfoundland (s)	Lofoten (m)	Suffolk	3,596.5	355.6	301.9	0.082	0.0245
Newfoundland (s)	Bornholm Basin	Suffolk	3,801.8	389.0	267.9	0.184	0.0001
Newfoundland (s)	Iceland (s)	Suffolk	3,576.3	342.8	247.8	0.161	0.0001
Newfoundland (s)	Møre (m)	Suffolk	4,072.1	399.8	307.8	0.130	0.0042
Newfoundland (s)	Newfoundland (m)	Kiel Bight	4,228.3	308.4	278.4	0.051	0.0353
Newfoundland (s)	Labrador	Kiel Bight	4,130.4	313.1	284.5	0.048	0.0419
Newfoundland (s)	Iceland (m)	Kiel Bight	4,146.2	416.1	314.0	0.140	0.0043
Newfoundland (s)	Lofoten (m)	Kiel Bight	3,645.5	357.0	310.0	0.071	0.0542
Newfoundland (s)	Bornholm Basin	Kiel Bight	3,833.9	406.4	279.6	0.185	0.0001
Newfoundland (s)	Iceland (s)	Kiel Bight	3,624.7	348.2	256.6	0.151	0.0005
Newfoundland (s)	Møre (m)	Kiel Bight	4,115.3	412.1	317.6	0.130	0.0031
Newfoundland (s)	Labrador	Lofoten (s)	3,914.1	285.2	261.1	0.044	0.0461
Newfoundland (s)	Iceland (m)	Lofoten (s)	3,933.1	381.0	290.6	0.135	0.0049
Newfoundland (s)	Lofoten (m)	Lofoten (s)	3,471.0	339.4	291.5	0.076	0.0446
Newfoundland (s)	Bornholm Basin	Lofoten (s)	3,649.7	383.3	258.6	0.194	0.0001
Newfoundland (s)	Iceland (s)	Lofoten (s)	3,477.8	324.7	238.8	0.152	0.0002
Newfoundland (s)	Møre (m)	Lofoten (s)	3,909.0	379.8	294.3	0.127	0.0045
Møre (m)	Iceland (m)	Newfoundland (s)	891.3	625.1	588.6	0.030	0.0532
Møre (m)	Bornholm Basin	Newfoundland (s)	856.1	574.5	520.8	0.049	0.0155
Møre (m)	Iceland (s)	Newfoundland (s)	776.0	546.4	471.5	0.074	0.0051
Møre (m)	Iceland (m)	Newfoundland (m)	882.2	636.2	588.7	0.039	0.0303
Møre (m)	Bornholm Basin	Newfoundland (m)	846.9	577.2	520.6	0.052	0.0203
Møre (m)	Iceland (s)	Newfoundland (m)	767.2	560.9	467.3	0.091	0.0000
Newfoundland (s)	Labrador	Iceland (m)	793.8	661.6	600.1	0.049	0.0070
Newfoundland (s)	Labrador	Lofoten (m)	761.2	602.8	559.8	0.037	0.0037
Newfoundland (s)	Labrador	Bornholm Basin	737.1	627.0	572.6	0.045	0.0353
Newfoundland (s)	Labrador	Iceland (s)	648.7	575.6	521.9	0.049	0.0119
Newfoundland (s)	Labrador	Møre (m)	819.9	652.1	588.0	0.052	0.0029
Møre (m)	Bornholm Basin	Lofoten (m)	639.9	630.7	592.8	0.031	0.0870
Iceland (m)	Lofoten (m)	Iceland (s)	547.9	600.6	559.8	0.035	0.0693

Supplementary Table 12: D_{BBAA}-statistics for *Gadus morhua* populations and outgroups within the supergene region on LG 7.
The D_{BBAA} version of the D-statistic[27] was calculated as for Supplementary Table 6, except that only sites from the supergene region on LG 7 were used. The highest D values are underlined.

P1	P2	P3	C_{BBAA}	C_{ABBA}	C_{BABA}	D_{BBAA}	p
Lofoten (m)	*G. chalcogrammus*	*G. macrocephalus*	9,783.1	1,774.3	1,527.7	0.075	0.0000
Labrador	Møre (s)	*G. macrocephalus*	19,051.8	655.2	571.7	0.068	0.0139
Lofoten (m)	Bornholm Basin	*G. macrocephalus*	17,032.2	599.9	511.2	0.080	0.0067
Labrador	Lofoten (s)	*G. macrocephalus*	18,511.5	638.6	565.2	0.061	0.0098
Labrador	Suffolk	*G. macrocephalus*	19,593.7	664.2	582.2	0.066	0.0066
Labrador	Kiel Bight	*G. macrocephalus*	19,806.5	667.7	572.7	0.077	0.0017
Labrador	Newfoundland (m)	*G. macrocephalus*	30,404.5	130.4	118.6	0.048	0.0301
Labrador	Newfoundland (s)	*G. macrocephalus*	30,383.7	122.5	111.2	0.048	0.0655
Lofoten (m)	Iceland (m)	*G. macrocephalus*	28,753.7	131.1	116.6	0.058	0.0394
Lofoten (m)	Møre (m)	*G. macrocephalus*	28,871.1	128.8	114.2	0.060	0.0676
G. macrocephalus	*G. ogac*	*G. chalcogrammus*	26,488.1	215.9	149.9	0.180	0.0000
G. macrocephalus	*G. ogac*	Møre (s)	25,584.8	240.8	152.9	0.223	0.0000
G. macrocephalus	*G. ogac*	Bornholm Basin	24,421.1	241.8	145.5	0.249	0.0000
G. macrocephalus	*G. ogac*	Lofoten (s)	24,835.1	229.9	151.7	0.205	0.0000
G. macrocephalus	*G. ogac*	Suffolk	26,375.0	248.0	160.5	0.214	0.0000
G. macrocephalus	*G. ogac*	Kiel Bight	26,699.2	247.8	158.5	0.220	0.0000
G. macrocephalus	*G. ogac*	Newfoundland (m)	26,935.1	313.9	154.9	0.339	0.0000
G. macrocephalus	*G. ogac*	Newfoundland (s)	26,915.3	317.4	151.6	0.354	0.0000
G. macrocephalus	*G. ogac*	Labrador	26,105.4	304.8	147.0	0.349	0.0000
G. macrocephalus	*G. ogac*	Iceland (m)	27,210.5	317.7	154.9	0.344	0.0000
G. macrocephalus	*G. ogac*	Iceland (s)	23,784.4	286.6	130.1	<u>0.376</u>	0.0000
G. macrocephalus	*G. ogac*	Møre (m)	27,158.5	317.1	154.5	0.345	0.0000
G. macrocephalus	*G. ogac*	Lofoten (m)	24,258.4	285.7	136.6	0.353	0.0000
Lofoten (m)	Møre (s)	*G. chalcogrammus*	10,368.6	1,050.6	967.4	0.041	0.0414
Lofoten (m)	Bornholm Basin	*G. chalcogrammus*	9,899.0	999.6	920.9	0.041	0.0375
Lofoten (m)	Lofoten (s)	*G. chalcogrammus*	10,105.6	1,053.3	952.7	0.050	0.0151
Lofoten (m)	Suffolk	*G. chalcogrammus*	10,589.6	1,068.7	977.6	0.044	0.0259
Lofoten (m)	Kiel Bight	*G. chalcogrammus*	10,736.8	1,077.0	983.4	0.045	0.0339
Lofoten (m)	Møre (m)	*G. chalcogrammus*	20,737.2	173.1	150.0	0.072	0.0138
Labrador	Newfoundland (m)	Møre (s)	11,167.1	343.9	313.4	0.046	0.0207
Labrador	Iceland (m)	Møre (s)	10,976.7	379.2	343.2	0.050	0.0397
Labrador	Møre (m)	Møre (s)	10,921.5	390.2	353.8	0.049	0.0812
Møre (s)	Lofoten (s)	Bornholm Basin	1,266.1	1,216.4	1,142.6	0.031	0.0089
Labrador	Newfoundland (m)	Bornholm Basin	10,769.9	328.2	302.0	0.042	0.0593
Lofoten (s)	Bornholm Basin	Newfoundland (s)	15,742.8	227.9	210.3	0.040	0.0900
Lofoten (s)	Bornholm Basin	Labrador	15,430.6	224.1	207.1	0.039	0.0866
Newfoundland (s)	Iceland (m)	Bornholm Basin	10,824.1	354.0	320.3	0.050	0.0452
Suffolk	Bornholm Basin	Iceland (s)	14,357.4	197.8	180.0	0.047	0.0580
Suffolk	Bornholm Basin	Møre (m)	16,305.9	225.9	209.4	0.038	0.0960
Suffolk	Bornholm Basin	Lofoten (m)	14,674.8	200.7	184.9	0.041	0.0828
Labrador	Newfoundland (m)	Lofoten (s)	11,054.0	333.8	309.9	0.037	0.0482
Newfoundland (s)	Iceland (m)	Lofoten (s)	11,086.1	364.3	330.7	0.048	0.0601
Newfoundland (s)	Iceland (m)	Suffolk	11,448.1	374.7	344.3	0.042	0.0826
Labrador	Newfoundland (m)	Kiel Bight	11,467.6	350.8	322.7	0.042	0.0466

Supplementary Table 12 (continued): D_{BBAA}-statistics for *Gadus morhua* populations and outgroups within the supergene region on LG 7.

P1	P2	P3	C_{BBAA}	C_{ABBA}	C_{BABA}	D_{BBAA}	p
Labrador	Iceland (m)	Kiel Bight	11,283.4	385.2	355.0	0.041	0.0632
Iceland (s)	Iceland (m)	Newfoundland (m)	1,486.2	1,023.8	945.8	0.040	0.0070
Lofoten (m)	Iceland (m)	Newfoundland (s)	1,524.5	1,144.1	1,044.4	0.046	0.0066
Newfoundland (m)	Newfoundland (s)	Møre (m)	1,564.5	1,221.2	1,119.0	0.044	0.0665
Lofoten (m)	Iceland (m)	Labrador	1,557.8	1,172.0	1,056.5	0.052	0.0026
Newfoundland (m)	Labrador	Iceland (s)	1,424.0	1,082.1	997.6	0.041	0.0202
Newfoundland (m)	Labrador	Møre (m)	1,604.8	1,206.8	1,127.5	0.034	0.0502
Lofoten (m)	Møre (m)	Iceland (m)	1,313.3	1,285.3	1,150.1	0.056	0.0017

Supplementary Table 13: D_{fix}-statistics for *Gadus morhua* populations and outgroups within the supergene region on LG 7.
The D_{fix} version of the D-statistic[27] was calculated as for Supplementary Table 6, except that only sites from the supergene region on LG 7 were used and the input tree was the one inferred for the supergene on LG 7 under the multi-species coalescent model (Fig. 3g). The highest D values are underlined.

P1	P2	P3	C_{BBAA}	C_{ABBA}	C_{BABA}	D_{BBAA}	p
Lofoten (m)	*G. chalcogrammus*	*G. macrocephalus*	9,783.1	1,774.3	1,527.7	0.075	0.0000
Labrador	Møre (s)	*G. macrocephalus*	19,051.8	655.2	571.7	0.068	0.0139
Lofoten (m)	Bornholm Basin	*G. macrocephalus*	17,032.2	599.9	511.2	0.080	0.0067
Labrador	Lofoten (s)	*G. macrocephalus*	18,511.5	638.6	565.2	0.061	0.0098
Labrador	Suffolk	*G. macrocephalus*	19,593.7	664.2	582.2	0.066	0.0066
Labrador	Kiel Bight	*G. macrocephalus*	19,806.5	667.7	572.7	0.077	0.0017
Labrador	Newfoundland (m)	*G. macrocephalus*	30,404.5	130.4	118.6	0.048	0.0301
Labrador	Newfoundland (s)	*G. macrocephalus*	30,383.7	122.5	111.2	0.048	0.0655
Lofoten (m)	Iceland (m)	*G. macrocephalus*	28,753.7	131.1	116.6	0.058	0.0394
Lofoten (m)	Møre (m)	*G. macrocephalus*	28,871.1	128.8	114.2	0.060	0.0676
G. macrocephalus	*G. ogac*	*G. chalcogrammus*	26,488.1	215.9	149.9	0.180	0.0000
G. macrocephalus	*G. ogac*	Møre (s)	25,584.8	240.8	152.9	0.223	0.0000
G. macrocephalus	*G. ogac*	Bornholm Basin	24,421.1	241.8	145.5	0.249	0.0000
G. macrocephalus	*G. ogac*	Lofoten (s)	24,835.1	229.9	151.7	0.205	0.0000
G. macrocephalus	*G. ogac*	Suffolk	26,375.0	248.0	160.5	0.214	0.0000
G. macrocephalus	*G. ogac*	Kiel Bight	26,699.2	247.8	158.5	0.220	0.0000
G. macrocephalus	*G. ogac*	Newfoundland (m)	26,935.1	313.9	154.9	0.339	0.0000
G. macrocephalus	*G. ogac*	Newfoundland (s)	26,915.3	317.4	151.6	0.354	0.0000
G. macrocephalus	*G. ogac*	Labrador	26,105.4	304.8	147.0	0.349	0.0000
G. macrocephalus	*G. ogac*	Iceland (m)	27,210.5	317.7	154.9	0.344	0.0000
G. macrocephalus	*G. ogac*	Iceland (s)	23,784.4	286.6	130.1	<u>0.376</u>	0.0000
G. macrocephalus	*G. ogac*	Møre (m)	27,158.5	317.1	154.5	0.345	0.0000
G. macrocephalus	*G. ogac*	Lofoten (m)	24,258.4	285.7	136.6	0.353	0.0000
Lofoten (m)	Møre (s)	*G. chalcogrammus*	10,368.6	1,050.6	967.4	0.041	0.0414
Lofoten (m)	Bornholm Basin	*G. chalcogrammus*	9,899.0	999.6	920.9	0.041	0.0375

Supplementary Table 13 (continued): D_{fix}-statistics for *Gadus morhua* populations and outgroups within the supergene region on LG 7.

P1	P2	P3	C_{BBAA}	C_{ABBA}	C_{BABA}	D_{BBAA}	p
Lofoten (m)	Lofoten (s)	*G. chalcogrammus*	10,105.6	1,053.3	952.7	0.050	0.0151
Lofoten (m)	Suffolk	*G. chalcogrammus*	10,589.6	1,068.7	977.6	0.044	0.0259
Lofoten (m)	Kiel Bight	*G. chalcogrammus*	10,736.8	1,077.0	983.4	0.045	0.0339
Lofoten (m)	Møre (m)	*G. chalcogrammus*	20,737.2	173.1	150.0	0.072	0.0138
Kiel Bight	Lofoten (s)	Møre (s)	1,217.9	1,376.1	1,174.9	0.079	0.0001
Kiel Bight	Suffolk	Møre (s)	1,234.6	1,307.1	1,193.7	0.045	0.0015
Labrador	Newfoundland (m)	Møre (s)	11,167.1	343.9	313.4	0.046	0.0207
Labrador	Iceland (m)	Møre (s)	10,976.7	379.2	343.2	0.050	0.0397
Labrador	Møre (m)	Møre (s)	10,921.5	390.2	353.8	0.049	0.0812
Møre (s)	Lofoten (s)	Bornholm Basin	1,266.1	1,216.4	1,142.6	0.031	0.0089
Labrador	Newfoundland (m)	Bornholm Basin	10,769.9	328.2	302.0	0.042	0.0593
Lofoten (s)	Bornholm Basin	Newfoundland (s)	15,742.8	227.9	210.3	0.040	0.0900
Lofoten (s)	Bornholm Basin	Labrador	15,430.6	224.1	207.1	0.039	0.0866
Newfoundland (s)	Iceland (m)	Bornholm Basin	10,824.1	354.0	320.3	0.050	0.0452
Suffolk	Bornholm Basin	Iceland (s)	14,357.4	197.8	180.0	0.047	0.0580
Suffolk	Bornholm Basin	Møre (m)	16,305.9	225.9	209.4	0.038	0.0960
Suffolk	Bornholm Basin	Lofoten (m)	14,674.8	200.7	184.9	0.041	0.0828
Kiel Bight	Suffolk	Lofoten (s)	1,230.0	1,310.7	1,232.1	0.031	0.0259
Labrador	Newfoundland (m)	Lofoten (s)	11,054.0	333.8	309.9	0.037	0.0482
Newfoundland (s)	Iceland (m)	Lofoten (s)	11,086.1	364.3	330.7	0.048	0.0601
Newfoundland (s)	Iceland (m)	Suffolk	11,448.1	374.7	344.3	0.042	0.0826
Labrador	Newfoundland (m)	Kiel Bight	11,467.6	350.8	322.7	0.042	0.0466
Labrador	Iceland (m)	Kiel Bight	11,283.4	385.2	355.0	0.041	0.0632
Iceland (s)	Iceland (m)	Newfoundland (m)	1,486.2	1,023.8	945.8	0.040	0.0070
Lofoten (m)	Iceland (m)	Newfoundland (s)	1,524.5	1,144.1	1,044.4	0.046	0.0066
Newfoundland (m)	Newfoundland (s)	Møre (m)	1,564.5	1,221.2	1,119.0	0.044	0.0665
Lofoten (m)	Iceland (m)	Labrador	1,557.8	1,172.0	1,056.5	0.052	0.0026
Newfoundland (m)	Labrador	Iceland (s)	1,424.0	1,082.1	997.6	0.041	0.0202
Newfoundland (m)	Labrador	Møre (m)	1,604.8	1,206.8	1,127.5	0.034	0.0502
Lofoten (m)	Møre (m)	Iceland (m)	1,313.3	1,285.3	1,150.1	0.056	0.0017
Iceland (m)	Iceland (s)	Lofoten (m)	1,077.1	1,165.1	1,035.7	0.059	0.0002

Supplementary Table 14: D_{BBAA}-statistics for *Gadus morhua* populations and outgroups within the supergene region on LG 12.
The D_{BBAA} version of the D-statistic[27] was calculated as for Supplementary Table 6, except that only sites from the supergene region on LG 12 were used. The highest D values are underlined.

P1	P2	P3	C_{BBAA}	C_{ABBA}	C_{BABA}	D_{BBAA}	p
Bornholm Basin	*G. chalcogrammus*	*G. macrocephalus*	13,979.6	2,751.8	2,566.6	0.035	0.0179
Bornholm Basin	Lofoten (s)	*G. macrocephalus*	23,094.0	363.2	293.2	0.107	0.0094
Bornholm Basin	Suffolk	*G. macrocephalus*	33,148.2	491.0	391.6	0.113	0.0005
Bornholm Basin	Kiel Bight	*G. macrocephalus*	33,505.7	485.5	408.0	0.087	0.0026
G. macrocephalus	*G. ogac*	*G. chalcogrammus*	34,681.2	296.2	226.2	0.134	0.0009
G. macrocephalus	*G. ogac*	Lofoten (s)	23,711.0	297.2	165.5	0.285	0.0000
G. macrocephalus	*G. ogac*	Suffolk	34,836.4	414.9	232.1	0.283	0.0000
G. macrocephalus	*G. ogac*	Kiel Bight	35,330.2	415.8	237.6	0.273	0.0000
G. macrocephalus	*G. ogac*	Bornholm Basin	32,867.1	423.3	210.6	0.336	0.0000
G. macrocephalus	*G. ogac*	Iceland (s)	31,577.5	395.5	197.0	0.335	0.0000
G. macrocephalus	*G. ogac*	Møre (s)	33,823.1	438.9	213.1	0.346	0.0000
G. macrocephalus	*G. ogac*	Lofoten (m)	31,503.6	410.2	188.9	<u>0.369</u>	0.0000
G. macrocephalus	*G. ogac*	Newfoundland (s)	35,366.2	449.3	220.4	0.342	0.0000
G. macrocephalus	*G. ogac*	Iceland (m)	35,865.7	458.8	226.7	0.339	0.0000
G. macrocephalus	*G. ogac*	Møre (m)	35,777.5	454.6	222.2	0.343	0.0000
G. macrocephalus	*G. ogac*	Newfoundland (m)	35,303.2	448.4	221.6	0.338	0.0000
G. macrocephalus	*G. ogac*	Labrador	34,386.4	432.6	215.0	0.336	0.0000
Iceland (m)	Lofoten (s)	*G. chalcogrammus*	16,586.6	518.0	464.1	0.055	0.0767
Iceland (m)	Suffolk	*G. chalcogrammus*	23,884.5	730.8	596.9	0.101	0.0016
Iceland (m)	Kiel Bight	*G. chalcogrammus*	24,186.9	711.4	638.1	0.054	0.0294
Iceland (m)	Iceland (s)	*G. chalcogrammus*	25,400.4	252.7	231.1	0.045	0.0345
Iceland (m)	Møre (s)	*G. chalcogrammus*	27,557.4	291.4	257.2	0.062	0.0308
Iceland (m)	Newfoundland (s)	*G. chalcogrammus*	28,062.6	319.1	287.4	0.052	0.0677
Iceland (m)	Møre (m)	*G. chalcogrammus*	28,871.7	307.4	277.0	0.052	0.0124
Iceland (m)	Newfoundland (m)	*G. chalcogrammus*	28,164.5	311.6	273.2	0.066	0.0050
Iceland (m)	Labrador	*G. chalcogrammus*	27,617.1	329.3	282.9	0.076	0.0073
Lofoten (m)	Bornholm Basin	Lofoten (s)	3,652.4	704.3	604.1	0.077	0.0066
Lofoten (m)	Iceland (s)	Lofoten (s)	3,520.5	631.9	533.6	0.084	0.0142
Lofoten (m)	Møre (s)	Lofoten (s)	3,810.3	656.3	605.3	0.040	0.0724
Labrador	Newfoundland (s)	Lofoten (s)	3,973.7	730.0	680.4	0.035	0.0978
Lofoten (m)	Iceland (m)	Lofoten (s)	3,886.5	678.8	629.7	0.038	0.0764
Lofoten (m)	Møre (m)	Lofoten (s)	3,890.5	683.1	633.7	0.037	0.0395
Labrador	Newfoundland (m)	Lofoten (s)	4,015.7	733.7	661.9	0.051	0.0681
Lofoten (m)	Bornholm Basin	Suffolk	4,839.8	962.0	811.7	0.085	0.0087
Lofoten (m)	Iceland (s)	Suffolk	4,586.9	824.6	742.6	0.052	0.0313
Labrador	Newfoundland (s)	Suffolk	5,323.9	999.3	940.4	0.030	0.0808
Lofoten (m)	Møre (m)	Suffolk	5,194.6	919.9	871.4	0.027	0.0930
Labrador	Newfoundland (m)	Suffolk	5,359.6	987.9	908.7	0.042	0.0809
Lofoten (m)	Bornholm Basin	Kiel Bight	4,878.6	974.6	826.1	0.083	0.0051
Lofoten (m)	Iceland (s)	Kiel Bight	4,620.7	840.4	744.6	0.060	0.0127
Lofoten (m)	Møre (s)	Kiel Bight	5,100.4	893.7	840.1	0.031	0.0948
Labrador	Newfoundland (s)	Kiel Bight	5,388.7	1,016.3	948.1	0.035	0.0776
Lofoten (m)	Møre (m)	Kiel Bight	5,237.2	930.3	881.9	0.027	0.0946

Supplementary Table 14 (continued): D_{BBAA}-statistics for *Gadus morhua* populations and outgroups within the supergene region on LG 12.

P1	P2	P3	C_{BBAA}	C_{ABBA}	C_{BABA}	D_{BBAA}	p
Labrador	Newfoundland (m)	Kiel Bight	5,408.1	1,017.8	910.1	0.056	0.0313
Møre (m)	Møre (s)	Bornholm Basin	1,590.3	1,549.4	1,383.9	0.056	0.0231
Newfoundland (s)	Newfoundland (m)	Bornholm Basin	1,701.7	1,481.1	1,361.7	0.042	0.0328
Lofoten (m)	Iceland (s)	Newfoundland (m)	1,506.7	1,308.6	1,188.6	0.048	0.0028
Iceland (m)	Møre (s)	Lofoten (m)	1,531.2	1,520.1	1,408.7	0.038	0.0382
Lofoten (m)	Møre (s)	Newfoundland (s)	1,804.9	1,383.2	1,325.4	0.021	0.0511
Iceland (m)	Møre (s)	Møre (m)	1,564.0	1,557.7	1,474.1	0.028	0.0635
Newfoundland (s)	Newfoundland (m)	Møre (s)	1,671.3	1,571.4	1,405.3	0.056	0.0028
Bornholm Basin	Lofoten (m)	Møre (m)	1,530.5	1,521.2	1,414.7	0.036	0.0315
Newfoundland (s)	Newfoundland (m)	Lofoten (m)	1,697.5	1,469.7	1,383.0	0.030	0.0413
Newfoundland (s)	Labrador	Lofoten (m)	1,637.4	1,532.4	1,440.6	0.031	0.0591
Lofoten (m)	Møre (m)	Newfoundland (s)	1,767.5	1,458.1	1,375.1	0.029	0.0200
Newfoundland (s)	Newfoundland (m)	Iceland (m)	1,743.8	1,583.1	1,440.8	0.047	0.0052
Lofoten (m)	Iceland (m)	Labrador	1,719.6	1,505.2	1,426.9	0.027	0.0384

Supplementary Table 15: D_{fix}-statistics for *Gadus morhua* populations and outgroups within the supergene region on LG 12.

The D_{fix} version of the D-statistic[27] was calculated as for Supplementary Table 6, except that only sites from the supergene region on LG 12 were used and the input tree was the one inferred for the supergene on LG 12 under the multi-species coalescent model (Fig. 3j). The highest D values are underlined.

P1	P2	P3	C_{BBAA}	C_{ABBA}	C_{BABA}	D_{BBAA}	p
Bornholm Basin	*G. chalcogrammus*	*G. macrocephalus*	13,979.6	2,751.8	2,566.6	0.035	0.0179
Bornholm Basin	Lofoten (s)	*G. macrocephalus*	23,094.0	363.2	293.2	0.107	0.0094
Bornholm Basin	Suffolk	*G. macrocephalus*	33,148.2	491.0	391.6	0.113	0.0005
Bornholm Basin	Kiel Bight	*G. macrocephalus*	33,505.7	485.5	408.0	0.087	0.0026
G. macrocephalus	*G. ogac*	*G. chalcogrammus*	34,681.2	296.2	226.2	0.134	0.0009
G. macrocephalus	*G. ogac*	Lofoten (s)	23,711.0	297.2	165.5	0.285	0.0000
G. macrocephalus	*G. ogac*	Suffolk	34,836.4	414.9	232.1	0.283	0.0000
G. macrocephalus	*G. ogac*	Kiel Bight	35,330.2	415.8	237.6	0.273	0.0000
G. macrocephalus	*G. ogac*	Bornholm Basin	32,867.1	423.3	210.6	0.336	0.0000
G. macrocephalus	*G. ogac*	Iceland (s)	31,577.5	395.5	197.0	0.335	0.0000
G. macrocephalus	*G. ogac*	Møre (s)	33,823.1	438.9	213.1	0.346	0.0000
G. macrocephalus	*G. ogac*	Lofoten (m)	31,503.6	410.2	188.9	<u>0.369</u>	0.0000
G. macrocephalus	*G. ogac*	Newfoundland (s)	35,366.2	449.3	220.4	0.342	0.0000
G. macrocephalus	*G. ogac*	Iceland (m)	35,865.7	458.8	226.7	0.339	0.0000
G. macrocephalus	*G. ogac*	Møre (m)	35,777.5	454.6	222.2	0.343	0.0000
G. macrocephalus	*G. ogac*	Newfoundland (m)	35,303.2	448.4	221.6	0.338	0.0000
G. macrocephalus	*G. ogac*	Labrador	34,386.4	432.6	215.0	0.336	0.0000
Iceland (m)	Lofoten (s)	*G. chalcogrammus*	16,586.6	518.0	464.1	0.055	0.0767
Iceland (m)	Suffolk	*G. chalcogrammus*	23,884.5	730.8	596.9	0.101	0.0016

Supplementary Table 15 (continued): D_{fix}-statistics for *Gadus morhua* populations and outgroups within the supergene region on LG 12.

P1	P2	P3	C_{BBAA}	C_{ABBA}	C_{BABA}	D_{BBAA}	p
Iceland (m)	Kiel Bight	*G. chalcogrammus*	24,186.9	711.4	638.1	0.054	0.0294
Iceland (m)	Iceland (s)	*G. chalcogrammus*	25,400.4	252.7	231.1	0.045	0.0345
Iceland (m)	Møre (s)	*G. chalcogrammus*	27,557.4	291.4	257.2	0.062	0.0308
Iceland (m)	Newfoundland (s)	*G. chalcogrammus*	28,062.6	319.1	287.4	0.052	0.0677
Iceland (m)	Møre (m)	*G. chalcogrammus*	28,871.7	307.4	277.0	0.052	0.0124
Iceland (m)	Newfoundland (m)	*G. chalcogrammus*	28,164.5	311.6	273.2	0.066	0.0050
Iceland (m)	Labrador	*G. chalcogrammus*	27,617.1	329.3	282.9	0.076	0.0073
Lofoten (m)	Bornholm Basin	Lofoten (s)	3,652.4	704.3	604.1	0.077	0.0066
Lofoten (m)	Iceland (s)	Lofoten (s)	3,520.5	631.9	533.6	0.084	0.0142
Lofoten (m)	Møre (s)	Lofoten (s)	3,810.3	656.3	605.3	0.040	0.0724
Labrador	Newfoundland (s)	Lofoten (s)	3,973.7	730.0	680.4	0.035	0.0978
Lofoten (m)	Iceland (m)	Lofoten (s)	3,886.5	678.8	629.7	0.038	0.0764
Lofoten (m)	Møre (m)	Lofoten (s)	3,890.5	683.1	633.7	0.037	0.0395
Labrador	Newfoundland (m)	Lofoten (s)	4,015.7	733.7	661.9	0.051	0.0681
Lofoten (m)	Bornholm Basin	Suffolk	4,839.8	962.0	811.7	0.085	0.0087
Lofoten (m)	Iceland (s)	Suffolk	4,586.9	824.6	742.6	0.052	0.0313
Labrador	Newfoundland (s)	Suffolk	5,323.9	999.3	940.4	0.030	0.0808
Lofoten (m)	Møre (m)	Suffolk	5,194.6	919.9	871.4	0.027	0.0930
Labrador	Newfoundland (m)	Suffolk	5,359.6	987.9	908.7	0.042	0.0809
Lofoten (m)	Bornholm Basin	Kiel Bight	4,878.6	974.6	826.1	0.083	0.0051
Lofoten (m)	Iceland (s)	Kiel Bight	4,620.7	840.4	744.6	0.060	0.0127
Lofoten (m)	Møre (s)	Kiel Bight	5,100.4	893.7	840.1	0.031	0.0948
Labrador	Newfoundland (s)	Kiel Bight	5,388.7	1,016.3	948.1	0.035	0.0776
Lofoten (m)	Møre (m)	Kiel Bight	5,237.2	930.3	881.9	0.027	0.0946
Labrador	Newfoundland (m)	Kiel Bight	5,408.1	1,017.8	910.1	0.056	0.0313
Newfoundland (s)	Iceland (s)	Bornholm Basin	1,300.2	1,525.4	1,216.0	0.113	0.0036
Newfoundland (s)	Møre (s)	Bornholm Basin	1,399.1	1,783.7	1,320.9	0.149	0.0000
Newfoundland (s)	Lofoten (m)	Bornholm Basin	1,342.9	1,735.7	1,334.2	0.131	0.0005
Newfoundland (s)	Iceland (m)	Bornholm Basin	1,439.2	1,772.6	1,388.4	0.122	0.0016
Newfoundland (s)	Møre (m)	Bornholm Basin	1,495.2	1,707.4	1,408.6	0.096	0.0103
Newfoundland (s)	Newfoundland (m)	Bornholm Basin	1,701.7	1,481.1	1,361.7	0.042	0.0328
Newfoundland (s)	Møre (s)	Iceland (s)	1,213.9	1,635.0	1,248.8	0.134	0.0000
Newfoundland (s)	Lofoten (m)	Iceland (s)	1,206.7	1,572.0	1,273.6	0.105	0.0010
Newfoundland (s)	Iceland (m)	Iceland (s)	1,248.5	1,647.5	1,298.5	0.118	0.0006
Newfoundland (s)	Møre (m)	Iceland (s)	1,275.6	1,620.3	1,283.5	0.116	0.0022
Newfoundland (s)	Newfoundland (m)	Iceland (s)	1,509.4	1,395.0	1,292.7	0.038	0.0173
Lofoten (m)	Møre (s)	Newfoundland (s)	1,804.9	1,383.2	1,325.4	0.021	0.0511
Lofoten (m)	Møre (s)	Iceland (m)	1,520.1	1,531.2	1,408.7	0.042	0.0311
Newfoundland (s)	Møre (m)	Møre (s)	1,417.3	1,844.9	1,398.8	0.138	0.0001
Newfoundland (s)	Newfoundland (m)	Møre (s)	1,671.3	1,571.4	1,405.3	0.056	0.0028
Newfoundland (s)	Møre (m)	Lofoten (m)	1,458.1	1,767.5	1,375.1	0.125	0.0001
Newfoundland (s)	Newfoundland (m)	Lofoten (m)	1,697.5	1,469.7	1,383.0	0.030	0.0413
Newfoundland (s)	Labrador	Lofoten (m)	1,637.4	1,532.4	1,440.6	0.031	0.0591
Labrador	Newfoundland (s)	Newfoundland (m)	1,520.4	1,646.6	1,572.4	0.023	0.0919
Newfoundland (s)	Møre (m)	Iceland (m)	1,495.7	1,854.2	1,461.6	0.118	0.0006
Newfoundland (s)	Newfoundland (m)	Iceland (m)	1,743.8	1,583.1	1,440.8	0.047	0.0052
Lofoten (m)	Iceland (m)	Labrador	1,719.6	1,505.2	1,426.9	0.027	0.0384

Supplementary Table 16: Predicted genes within the region of the supergene on LG 12 with evidence for double crossover (positions 7,478,537 bp to 7,752,994 bp).
Gene IDs refer to the gadMor2 assembly annotation[10]. BLASTN searches suggest that gene ID 00027785 corresponds to vitellogenin type A, while both IDs 00027782 and 00027783 correspond to vitellogenin type B.

Beginning	End	Strand	Gene ID	Predicted gene	GO terms
7,468,870	7,485,996	−	00027772	TTLL7 (*Homo sapiens*)	GO:0006464
7,493,441	7,504,363	+	00027774	PRKACA (*Sus scrofa*)	GO:0004672, GO:0004674, GO:0005524, GO:0006468, GO:0016772
7,504,544	7,507,926	+	00027775	SAMD13 (*Homo sapiens*)	GO:0005515
7,507,198	7,513,354	−	00027776	UOX (*Oryctolagus cuniculus*)	—
7,512,454	7,516,738	+	00027777	DNASE2B (*Homo sapiens*)	GO:0004531, GO:0006259
7,517,560	7,520,918	+	00027779	rpf1 (*Danio rerio*)	—
7,526,726	7,530,804	−	00027781	Ctbs (*Mus musculus*)	GO:0004553, GO:0004568, GO:0005975, GO:0006032
7,534,022	7,547,615	+	00027782	vtg1 (*Oncorhynchus mykiss*)	GO:0005319, GO:0006869
7,551,466	7,559,692	−	00027783	Vitellogenin (*Fundulus heteroclitus*)	GO:0005319, GO:0006869
7,565,225	7,575,883	+	00027785	vtg1 (*Oncorhynchus mykiss*)	GO:0005319, GO:0006869
7,575,985	7,583,560	−	00027786	ssx2ip (*Oryzias latipes*)	—
7,604,691	7,610,866	−	00027788	Lpar3 (*Mus musculus*)	GO:0004930, GO:0007186, GO:0016021, GO:0070915
7,613,633	7,625,764	−	00027790	MCOLN2 (*Homo sapiens*)	—
7,627,573	7,633,411	−	00027791	CLCC1 (*Bos taurus*)	—
7,633,462	7,641,637	−	00027793	FTSJ3 (*Gallus gallus*)	GO:0001510, GO:0005634, GO:0006364, GO:0008168, GO:0008649, GO:0031167, GO:0032259
7,641,843	7,644,988	−	00027795	Hrasls (*Mus musculus*)	—
7,645,411	7,660,397	+	00027796	Mfn1 (*Rattus norvegicus*)	GO:0003924, GO:0005525, GO:0005741, GO:0008053, GO:0016021
7,660,315	7,678,716	−	00027799	GNB4 (*Homo sapiens*)	GO:0005515, GO:0016491, GO:0051537, GO:0055114
7,684,579	7,693,849	+	00027802	ACTL6A (*Homo sapiens*)	—
7,692,544	7,696,524	−	00027803	Mrpl47 (*Mus musculus*)	GO:0003735, GO:0005761, GO:0006412
7,696,509	7,699,595	+	00027804	NDUFB5 (*Bos taurus*)	—
7,700,430	7,730,778	+	00027805	usp13 (*Danio rerio*)	GO:0005515, GO:0006511, GO:0008270, GO:0036459
7,734,260	7,769,915	−	00027810	PEX5L (*Homo sapiens*)	GO:0005515
7,823,234	7,825,557	−	00027816	Cpn2 (*Mus musculus*)	GO:0005515

Supplementary Table 17: Genomic signatures of extreme bottlenecks.
To assess how long signatures of brief but extreme bottlenecks can remain in the genomes of the affected population, we simulated genetic data with msprime, under a model of a single panmictic population that experienced an extreme bottleneck in the past during which the population size was reduced to a single sequence for a duration of 20 generations. At the end of the simulation, one diploid genome was sampled from the population and the proportion of the diploid genome coalescing in (or before) the bottleneck, $p_{\text{bottleneck}}$, was quantified. We further quantified the genetic diversity of the simulated genome, $\pi_{\text{bottleneck}}$, as well as the genetic diversity of a second genome simulated with the same settings except the bottleneck, $\pi_{\text{no bottleneck}}$. The simulated linkage group had a length of 20 Mb and the generation time was assumed to be 10 years. We repeated the simulation with five different values for the diploid population size, N_{e}, (outside the bottleneck) between 1,000 and 100,000 and with five different values for the age of the bottleneck, $t_{\text{bottleneck}}$, between 3,000 years and 3,000,000 years. Reported are the mean values across 20 simulations followed by standard deviations in parentheses.

N_{e}	$t_{\text{bottleneck}}$	$p_{\text{bottleneck}}$	$\pi_{\text{bottleneck}}$ ($\times 10^3$)	$\pi_{\text{no bottleneck}}$ ($\times 10^3$)
1,000	3,000	0.89 (0.06)	0.004 (0.001)	0.030 (0.004)
1,000	10,000	0.61 (0.07)	0.012 (0.002)	0.031 (0.003)
1,000	30,000	0.22 (0.04)	0.025 (0.003)	0.032 (0.004)
1,000	100,000	0.01 (0.00)	0.031 (0.003)	0.031 (0.003)
1,000	300,000	0.00 (0.00)	0.032 (0.003)	0.030 (0.004)
1,000	1,000,000	0.00 (0.00)	0.032 (0.003)	0.031 (0.004)
1,000	3,000,000	0.00 (0.00)	0.031 (0.004)	0.030 (0.005)
3,000	3,000	0.96 (0.05)	0.005 (0.001)	0.094 (0.009)
3,000	10,000	0.85 (0.05)	0.015 (0.001)	0.093 (0.008)
3,000	30,000	0.59 (0.05)	0.038 (0.003)	0.090 (0.011)
3,000	100,000	0.19 (0.01)	0.078 (0.005)	0.095 (0.011)
3,000	300,000	0.01 (0.00)	0.095 (0.005)	0.093 (0.010)
3,000	1,000,000	0.00 (0.00)	0.095 (0.005)	0.096 (0.007)
3,000	3,000,000	0.00 (0.00)	0.097 (0.005)	0.092 (0.009)
10,000	3,000	0.98 (0.03)	0.005 (0.000)	0.318 (0.011)
10,000	10,000	0.95 (0.02)	0.016 (0.001)	0.307 (0.011)
10,000	30,000	0.86 (0.02)	0.044 (0.002)	0.314 (0.013)
10,000	100,000	0.61 (0.02)	0.123 (0.005)	0.312 (0.018)
10,000	300,000	0.22 (0.01)	0.242 (0.010)	0.304 (0.035)
10,000	1,000,000	0.01 (0.00)	0.315 (0.011)	0.314 (0.013)
10,000	3,000,000	0.00 (0.00)	0.316 (0.016)	0.315 (0.015)
30,000	3,000	0.99 (0.03)	0.005 (0.001)	0.946 (0.024)
30,000	10,000	0.99 (0.01)	0.016 (0.001)	0.953 (0.018)
30,000	30,000	0.96 (0.01)	0.047 (0.002)	0.939 (0.038)
30,000	100,000	0.84 (0.04)	0.144 (0.005)	0.940 (0.019)
30,000	300,000	0.61 (0.02)	0.369 (0.007)	0.943 (0.015)
30,000	1,000,000	0.19 (0.01)	0.762 (0.017)	0.944 (0.018)
30,000	3,000,000	0.01 (0.00)	0.942 (0.023)	0.939 (0.027)
100,000	3,000	1.00 (0.00)	0.005 (0.000)	3.122 (0.044)
100,000	10,000	1.00 (0.01)	0.016 (0.001)	3.132 (0.043)
100,000	30,000	0.98 (0.01)	0.047 (0.002)	3.139 (0.041)
100,000	100,000	0.95 (0.01)	0.152 (0.003)	3.124 (0.037)

Supplementary Table 17 (continued): Genomic signatures of extreme bottlenecks.

N_e	$t_{\text{bottleneck}}$	$p_{\text{bottleneck}}$	$\pi_{\text{bottleneck}}$ ($\times 10^3$)	$\pi_{\text{no bottleneck}}$ ($\times 10^3$)
100,000	300,000	0.86 (0.02)	0.437 (0.008)	3.138 (0.051)
100,000	1,000,000	0.61 (0.01)	1.236 (0.013)	3.122 (0.035)
100,000	3,000,000	0.22 (0.00)	2.444 (0.035)	3.131 (0.053)

Supplementary Table 18: *Gadus morhua* specimens used in analyses of linkage disequilibrium. Genomes of 100 *Gadus morhua* specimens were used exclusively in analyses of linkage disequilibrium and selected so that at each of the four LGs with supergenes, both the ancestral and the derived orientation of the supergene were found in exactly 50 specimens.

Specimen ID	ENA accession(s)	Population	LG 1	LG 2	LG 7	LG 12
AVE1403Z09	NA	Møre	derived	ancestral	derived	ancestral
AVE1403Z11	ERR5321906	Møre	derived	ancestral	derived	ancestral
AVE1403Z17	NA	Møre	derived	ancestral	derived	ancestral
AVE1403Z20	NA	Møre	derived	ancestral	derived	ancestral
AVE1403Z27	NA	Møre	derived	ancestral	derived	ancestral
AVE1403Z29	NA	Møre	derived	ancestral	derived	ancestral
AVE1403Z34	NA	Møre	derived	ancestral	derived	ancestral
AVE1403Z35	NA	Møre	derived	ancestral	derived	ancestral
CEL1002Z01	NA	Celtic Sea	ancestral	derived	ancestral	derived
CEL1002Z02	NA	Celtic Sea	ancestral	derived	ancestral	derived
CEL1002Z08	NA	Celtic Sea	ancestral	derived	ancestral	derived
CEL1002Z10	NA	Celtic Sea	ancestral	derived	ancestral	derived
CEL1002Z12	NA	Celtic Sea	ancestral	derived	ancestral	derived
CEL1002Z14	NA	Celtic Sea	ancestral	derived	ancestral	derived
CEL1002Z15	NA	Celtic Sea	ancestral	derived	ancestral	derived
CEL1002Z16	NA	Celtic Sea	ancestral	derived	ancestral	derived
CEL1002Z17	NA	Celtic Sea	ancestral	derived	ancestral	derived
CEL1002Z24	NA	Celtic Sea	ancestral	derived	ancestral	derived
ENG0701Z01	NA	English Channel	ancestral	derived	ancestral	derived
ENG0701Z02	NA	English Channel	ancestral	derived	ancestral	derived
ENG0701Z03	NA	English Channel	ancestral	derived	ancestral	derived
ENG0701Z05	NA	English Channel	ancestral	derived	ancestral	derived
ENG0701Z07	NA	English Channel	ancestral	derived	ancestral	derived
ENG0701Z09	NA	English Channel	ancestral	derived	ancestral	derived
ENG0701Z16	NA	English Channel	ancestral	derived	ancestral	derived
ENG0701Z20	NA	English Channel	ancestral	derived	ancestral	derived
ENG0701Z22	NA	English Channel	ancestral	derived	ancestral	derived
ENG0902Z01	NA	English Channel	ancestral	derived	ancestral	derived
ENG0902Z03	NA	English Channel	ancestral	derived	ancestral	derived
ENG0902Z04	NA	English Channel	ancestral	derived	ancestral	derived
ICC0304Z14	NA	Iceland	derived	ancestral	derived	ancestral
ICC0304Z15	NA	Iceland	derived	ancestral	derived	ancestral

Supplementary Table 18 (continued): *Gadus morhua* specimens used in analyses of linkage disequilibrium.

Specimen ID	ENA accession(s)	Population	LG 1	LG 2	LG 7	LG 12
ICC0304Z16	NA	Iceland	derived	ancestral	derived	ancestral
ICO0304Z01	NA	Iceland	derived	ancestral	derived	ancestral
ICO0304Z10	NA	Iceland	derived	ancestral	derived	ancestral
ICO0304Z11	NA	Iceland	derived	ancestral	derived	ancestral
ICO0304Z14	NA	Iceland	derived	ancestral	derived	ancestral
ICO0304Z15	NA	Iceland	derived	ancestral	derived	ancestral
ICO0304Z16	NA	Iceland	derived	ancestral	derived	ancestral
ICO0304Z19	NA	Iceland	derived	ancestral	derived	ancestral
ICO0304Z20	ERR5321899	Iceland	derived	ancestral	derived	ancestral
ICO0304Z21	NA	Iceland	derived	ancestral	derived	ancestral
IRI0802Z01	NA	Irish Sea	ancestral	derived	ancestral	derived
IRI0902Z02	NA	Irish Sea	ancestral	derived	ancestral	derived
IRI0902Z06	NA	Irish Sea	ancestral	derived	ancestral	derived
IRI0903Z01	NA	Irish Sea	ancestral	derived	ancestral	derived
IRI0903Z02	NA	Irish Sea	ancestral	derived	ancestral	derived
IRI0903Z03	NA	Irish Sea	ancestral	derived	ancestral	derived
IRI0903Z04	NA	Irish Sea	ancestral	derived	ancestral	derived
IRI1002Z03	NA	Irish Sea	ancestral	derived	ancestral	derived
IRI1003Z05	NA	Irish Sea	ancestral	derived	ancestral	derived
IRI1003Z07	NA	Irish Sea	ancestral	derived	ancestral	derived
IRI1003Z12	NA	Irish Sea	ancestral	derived	ancestral	derived
KIE1103Z20	ERR2850485	Kiel Bight	ancestral	derived	ancestral	derived
LOF1103Z04	ERR4851787,88	Lofoten	derived	ancestral	derived	ancestral
LOF1103Z06	ERR4851791,92	Lofoten	derived	ancestral	derived	ancestral
LOF1103Z08	ERR4851795,96	Lofoten	derived	ancestral	derived	ancestral
LOF1103Z10	ERR4851799,800	Lofoten	derived	ancestral	derived	ancestral
LOF1103Z11	ERR4851801,02	Lofoten	derived	ancestral	derived	ancestral
LOF1103Z17	ERR4851813,14	Lofoten	derived	ancestral	derived	ancestral
LOF1103Z20	ERR4851819,20	Lofoten	derived	ancestral	derived	ancestral
LOF1103Z24	ERR4851827,28	Lofoten	derived	ancestral	derived	ancestral
LOF1403Z02	ERR4851830	Lofoten	derived	ancestral	derived	ancestral
LOF1403Z04	ERR4851832	Lofoten	derived	ancestral	derived	ancestral
LOF1403Z06	ERR4851834	Lofoten	derived	ancestral	derived	ancestral
LOF1403Z07	ERR4851835	Lofoten	derived	ancestral	derived	ancestral
LOF1403Z08	ERR4851836	Lofoten	derived	ancestral	derived	ancestral
LOF1403Z09	ERR4851837	Lofoten	derived	ancestral	derived	ancestral
LOF1403Z11	ERR4851839	Lofoten	derived	ancestral	derived	ancestral
LOF1403Z12	ERR4851840	Lofoten	derived	ancestral	derived	ancestral
LOF1403Z14	ERR4851842	Lofoten	derived	ancestral	derived	ancestral
LOF1403Z17	ERR4851845	Lofoten	derived	ancestral	derived	ancestral
LOF1403Z18	ERR4851846	Lofoten	derived	ancestral	derived	ancestral
LOF1403Z24	ERR4851852	Lofoten	derived	ancestral	derived	ancestral
LOW1503Z01	ERR2850495-98	Suffolk	ancestral	derived	ancestral	derived
LOW1503Z04	ERR2850507-10	Suffolk	ancestral	derived	ancestral	derived
LOW1503Z07	ERR2850519-22	Suffolk	ancestral	derived	ancestral	derived
LOW1503Z08	ERR2850523-26	Suffolk	ancestral	derived	ancestral	derived
LOW1504Z02	ERR2850559-62	Suffolk	ancestral	derived	ancestral	derived

Supplementary Table 18 (continued): *Gadus morhua* specimens used in analyses of linkage disequilibrium.

Specimen ID	ENA accession(s)	Population	LG 1	LG 2	LG 7	LG 12
LOW1504Z05	ERR2850571-74	Suffolk	ancestral	derived	ancestral	derived
LOW1504Z06	ERR2850575-78	Suffolk	ancestral	derived	ancestral	derived
LOW1504Z07	ERR2850579-82	Suffolk	ancestral	derived	ancestral	derived
LOW1504Z08	ERR2850583-86	Suffolk	ancestral	derived	ancestral	derived
SCA0601Z04	NA	North Yorkshire	ancestral	derived	ancestral	derived
SCA0601Z12	NA	North Yorkshire	ancestral	derived	ancestral	derived
SOR1403Z01	NA	Finnmark	derived	ancestral	derived	ancestral
SOR1403Z02	NA	Finnmark	derived	ancestral	derived	ancestral
SOR1403Z07	NA	Finnmark	derived	ancestral	derived	ancestral
SOR1404Z02	NA	Finnmark	derived	ancestral	derived	ancestral
SOR1404Z03	NA	Finnmark	derived	ancestral	derived	ancestral
SOR1404Z05	NA	Finnmark	derived	ancestral	derived	ancestral
SOR1404Z06	NA	Finnmark	derived	ancestral	derived	ancestral
SOR1404Z09	NA	Finnmark	derived	ancestral	derived	ancestral
SOR1404Z10	NA	Finnmark	derived	ancestral	derived	ancestral
SOR1404Z12	NA	Finnmark	derived	ancestral	derived	ancestral
TVE1105Z09	ERR2850674	Agder	ancestral	derived	ancestral	derived
TVE1105Z19	ERR2850683	Agder	ancestral	derived	ancestral	derived
TVE1205Z01	ERR2850689	Agder	ancestral	derived	ancestral	derived
TVE1205Z10	ERR2850698	Agder	ancestral	derived	ancestral	derived
TVE1205Z13	ERR2850701	Agder	ancestral	derived	ancestral	derived

Supplementary Table 19: Sequence data used in analyses of divergence times of Gadinae. Illumina reads for *Gadus macrocephalus* and *Gadus ogac* were used for local assembly of candidate phylogenetic markers, all local and whole-genome assemblies were used to identify sets of orthologuous sequences for 91 genes, and alignments of these sequences were used to estimate divergence times with StarBEAST2[26, 42] (Supplementary Figure 5).

Species	Data type	Reference	Accession
Brosme brosme	Genome assemby	Malmstrøm et al.[8]	Dryad: 326r8
Gadiculus argenteus	Genome assemby	Malmstrøm et al.[8]	Dryad: 326r8
Trisopterus minutus	Genome assemby	Malmstrøm et al.[8]	Dryad: 326r8
Pollachius virens	Genome assemby	Malmstrøm et al.[8]	Dryad: 326r8
Melanogrammus aeglefinus	Genome assemby	Malmstrøm et al.[8]	Dryad: 326r8
Merlangius merlangus	Genome assemby	Malmstrøm et al.[8]	Dryad: 326r8
Boreogadus saida	Genome assemby	Malmstrøm et al.[8]	Dryad: 326r8
Arctogadus glacialis	Genome assemby	Malmstrøm et al.[8]	Dryad: 326r8
Gadus macrocephalus	Illumina reads	Árnason and Halldórsdóttir[1]	ENA: SRR2906345
Gadus ogac	Illumina reads	Árnason and Halldórsdóttir[1]	ENA: SRR2906193
Gadus chalcogrammus	Genome assemby	Malmstrøm et al.[8]	Dryad: 326r8
Gadus morhua	Genome assemby[1]	Tørresen et al.[10]	ENA: ERR1551885

[1]gadMor2 assembly[10]

Supplementary Table 20: Read data used in analyses of divergence times and introgression among species of the genera *Gadus*, *Arctogadus*, and *Boreogadus*.
Illumina reads were mapped to the gadMor2 assembly[10] and used to generate alignments for 109 phylogenetic markers, which were then analyzed with the isolation-with-migration model implemented in the AIM package[25] for BEAST 2[26].

Species	Reference	ENA accession(s)
Pollachius virens	Malmstrøm et al.[8]	ERR1473878
Melanogrammus aeglefinus	Tørresen et al.[39]	ERR2028455,ERR2028456
Merlangius merlangus	Malmstrøm et al.[8]	ERR1473880,ERR1473881
Boreogadus saida	Malmstrøm et al.[8]	ERR1473884,ERR1473885
Arctogadus glacialis	Malmstrøm et al.[8]	ERR1473882,ERR1473883
Gadus macrocephalus	Árnason and Halldórsdóttir[1]	SRR2906345
Gadus ogac	Árnason and Halldórsdóttir[1]	SRR2906193
Gadus chalcogrammus	Malmstrøm et al.[8]	ERR1473886
Gadus morhua (migratory)	This study	ERR5330167[1]
Gadus morhua (stationary)	This study	ERR5321904[2]

[1]Migratory *Gadus morhua* individual LOF1103Z24 sampled at the Lofoten islands, with a genome similar to the gadMor2 genome (2,940 differences excluding heterozygous sites)
[2]Stationary *Gadus morhua* individual LOF1106Z11 used for the gadMor_Stat assembly

Supplementary Table 21: Test statistics for comparisons of repeat content and mutation load in *Gadus morhua*.
Sample sizes are given as the numbers of sliding windows in the genome-wide background or the four supergenes. df: Degrees of freedom; CI: confidence interval; Prop.: proportion; AA: amino acid; GWBG: genome-wide background; SG: supergene.

Measure	Comparison	Sample sizes	Means	t	df	p	95%CI
Repeat content (gadMor2)	GWBG / LG 1 SG	544 / 17	$1.06 / 1.16 \times 10^{-1}$	0.790	16.816	0.220	$[-1.23 \times 10^{-2}, \infty]$
Repeat content (gadMor2)	GWBG / LG 2 SG	544 / 6	$1.06 / 1.57 \times 10^{-1}$	2.515	5.101	0.026	$[\ 1.04 \times 10^{-2}, \infty]$
Repeat content (gadMor2)	GWBG / LG 7 SG	544 / 9	$1.06 / 0.77 \times 10^{-1}$	-4.330	9.687	0.999	$[-4.13 \times 10^{-2}, \infty]$
Repeat content (gadMor2)	GWBG / LG 12 SG	544 / 13	$1.06 / 0.97 \times 10^{-1}$	-0.672	12.607	0.743	$[-3.18 \times 10^{-2}, \infty]$
Prop. stop codons (gadMor_Stat)	GWBG / LG 1 SG	544 / 17	$9.47 / 3.36 \times 10^{-5}$	1.881	16.381	0.039	$[\ 0.44 \times 10^{-5}, \infty]$
Prop. stop codons (gadMor_Stat)	GWBG / LG 2 SG	544 / 6	$9.47 / 5.90 \times 10^{-5}$	0.857	5.146	0.215	$[-3.36 \times 10^{-5}, \infty]$
Prop. stop codons (gadMor_Stat)	GWBG / LG 7 SG	544 / 9	$9.47 / 4.58 \times 10^{-5}$	0.582	8.481	0.288	$[-2.60 \times 10^{-5}, \infty]$
Prop. stop codons (gadMor_Stat)	GWBG / LG 12 SG	544 / 13	$9.47 / 4.75 \times 10^{-5}$	0.933	13.509	0.184	$[-1.21 \times 10^{-5}, \infty]$
Prop. changed AAs (gadMor2)	GWBG / LG 1 SG	544 / 17	$3.28 / 2.60 \times 10^{-2}$	-4.579	20.653	1.000	$[-0.93 \times 10^{-2}, \infty]$
Prop. changed AAs (gadMor2)	GWBG / LG 2 SG	544 / 6	$3.28 / 3.92 \times 10^{-2}$	1.535	5.155	0.092	$[-0.19 \times 10^{-2}, \infty]$
Prop. changed AAs (gadMor2)	GWBG / LG 7 SG	544 / 9	$3.28 / 2.63 \times 10^{-2}$	-2.654	8.760	0.987	$[-1.09 \times 10^{-2}, \infty]$
Prop. changed AAs (gadMor2)	GWBG / LG 12 SG	544 / 13	$3.28 / 3.31 \times 10^{-2}$	0.101	12.779	0.461	$[-0.49 \times 10^{-2}, \infty]$
Prop. changed AAs (gadMor_Stat)	GWBG / LG 1 SG	544 / 17	$3.28 / 2.57 \times 10^{-2}$	-4.887	20.660	1.000	$[-0.96 \times 10^{-2}, \infty]$
Prop. changed AAs (gadMor_Stat)	GWBG / LG 2 SG	544 / 6	$3.28 / 3.79 \times 10^{-2}$	1.267	5.157	0.130	$[-0.30 \times 10^{-2}, \infty]$
Prop. changed AAs (gadMor_Stat)	GWBG / LG 7 SG	544 / 9	$3.28 / 2.64 \times 10^{-2}$	-2.671	8.752	0.987	$[-1.08 \times 10^{-2}, \infty]$
Prop. changed AAs (gadMor_Stat)	GWBG / LG 12 SG	544 / 13	$3.28 / 3.19 \times 10^{-2}$	-0.338	13.015	0.630	$[-0.53 \times 10^{-2}, \infty]$

Supplementary Table 22: D_{BBAA}-statistics for species of the genera *Gadus*, *Arctogadus*, and *Boreogadus* and outgroups.

For the calculation of the D_{BBAA} version of the D-statistic[27], all trios are oriented so that the number of "BBAA" patterns (C_{BBAA}) is greater than the numbers of "ABBA" (C_{ABBA}) and "BBAA" (C_{BABA}) patterns. Additionally, the trio is arbitrarily oriented so that the number of "ABBA" patterns (C_{ABBA}) exceeds that of "BBAA" patterns (C_{BABA}). When the trio is oriented in this way, the D_{BBAA}-statistic is never negative and values significantly greater than zero support introgression between the two species in positions P2 and P3. If the same pair of species in positions P2 and P3 produced significant results with several third species in position P1, only the trio with the highest D_{BBAA} is listed. Significance was assessed with block-jackknifing. The highest D values are underlined.

P1	P2	P3	C_{BBAA}	C_{ABBA}	C_{BABA}	D_{BBAA}	p
G. morhua (m)[1]	*B. saida*	*Mel. aeglefinus*	999,222.0	74,770.5	70,196.2	0.032	0.0000
G. morhua (m)	*A. glacialis*	*Mel. aeglefinus*	1043,480.0	65,118.6	63,258.9	0.014	0.0000
G. morhua (m)	*G. macrocephal.*	*Mel. aeglefinus*	1,381,730.0	48,034.4	46,407.9	0.017	0.0000
G. morhua (m)	*G. ogac*	*Mel. aeglefinus*	1,378,670.0	47,735.4	45,982.6	0.019	0.0000
G. morhua (m)	*G. chalcogram.*	*Mel. aeglefinus*	1,630,450.0	32,523.8	31,345.2	0.018	0.0000
G. morhua (m)	*G. morhua* (s)[2]	*Mel. aeglefinus*	2,128,480.0	6,106.4	5,964.9	0.012	0.0360
Mel. aeglefinus	*Mer. merlangus*	*B. saida*	524,569.0	109,421.0	104,705.0	0.022	0.0000
Mel. aeglefinus	*Mer. merlangus*	*A. glacialis*	526,258.0	103,925.0	99,293.4	0.023	0.0000
Mel. aeglefinus	*Mer. merlangus*	*G. macrocephal.*	535,103.0	106,401.0	101,740.0	0.022	0.0000
Mel. aeglefinus	*Mer. merlangus*	*G. ogac*	532,068.0	105,833.0	101,330.0	0.022	0.0000
Mel. aeglefinus	*Mer. merlangus*	*G. chalcogram.*	534,655.0	107,694.0	101,792.0	0.028	0.0000
Mel. aeglefinus	*Mer. merlangus*	*G. morhua* (s)	544,694.0	110,805.0	103,491.0	0.034	0.0000
Mel. aeglefinus	*Mer. merlangus*	*G. morhua* (m)	544,885.0	110,788.0	103,421.0	0.034	0.0000
G. morhua (m)	*G. macrocephal.*	*B. saida*	461,022.0	87,150.0	74,312.0	0.080	0.0000
G. morhua (m)	*G. ogac*	*B. saida*	463,606.0	86,146.6	73,616.9	0.078	0.0000
G. morhua (m)	*G. chalcogram.*	*B. saida*	699,019.0	51,353.8	42,041.8	0.100	0.0000
G. morhua (m)	*G. morhua* (s)	*B. saida*	1,178,120.0	6,965.9	6,699.1	0.020	0.0004
B. saida	*A. glacialis*	*G. macrocephal.*	267,280.0	170,777.0	132,905.0	0.125	0.0000
B. saida	*A. glacialis*	*G. ogac*	265,999.0	169,897.0	132,210.0	0.125	0.0000
B. saida	*A. glacialis*	*G. chalcogram.*	268,779.0	170,122.0	131,604.0	0.128	0.0000
B. saida	*A. glacialis*	*G. morhua* (s)	279,966.0	170,630.0	131,786.0	0.128	0.0000
B. saida	*A. glacialis*	*G. morhua* (m)	280,258.0	170,613.0	131,776.0	0.128	0.0000
G. morhua (m)	*G. chalcogram.*	*G. macrocephal.*	337,874.0	74,069.5	55,581.5	0.143	0.0000
G. morhua (m)	*G. morhua* (s)	*G. macrocephal.*	820,109.0	7,930.4	7,495.9	0.028	0.0000
G. macrocephal.	*G. ogac*	*G. chalcogram.*	941,162.0	9,380.4	7,456.4	0.114	0.0000
G. macrocephal.	*G. ogac*	*G. morhua* (s)	980,086.0	13,956.1	7,797.4	<u>0.283</u>	0.0000
G. macrocephal.	*G. ogac*	*G. morhua* (m)	980,709.0	14,167.9	7,875.9	<u>0.285</u>	0.0000
G. morhua (m)	*G. morhua* (s)	*G. chalcogram.*	532,010.0	10,099.0	9,619.8	0.024	0.0000

[1]Migratory *Gadus morhua* individual LOF1103Z24 sampled at the Lofoten islands

[2]Stationary *Gadus morhua* individual LOF1106Z11 sampled at the Lofoten islands

Supplementary Table 23: D_{fix}-statistics for species of the genera *Gadus*, *Arctogadus*, and *Boreogadus* and outgroups.
For the calculation of the D_{fix} version of the D-statistic[27], all trios are fixed in their orientation according to an assumed species tree, for which we here use the more strongly supported one out of two trees resulting from the inference under the isolation-with-migration model (Extended Data Fig. 2b). Additionally, the trio is arbitrarily oriented so that the number of "ABBA" patterns (C_{ABBA}) exceeds that of "BBAA" patterns (C_{BABA}). When the trio is oriented in this way, the D_{BBAA}-statistic is never negative and values significantly greater than zero support introgression between the two species in positions P2 and P3. If the same pair of species in positions P2 and P3 produced significant results with several third species in position P1, only the trio with the highest D_{BBAA} is listed. Significance was assessed with block-jackknifing. The highest D values are underlined.

P1	P2	P3	C_{BBAA}	C_{ABBA}	C_{BABA}	D_{fix}	p
G. morhua (m)[1]	*B. saida*	*Mel. aeglefinus*	999,222.0	74,770.5	70,196.2	0.032	0.0000
G. morhua (m)	*A. glacialis*	*Mel. aeglefinus*	1,043,480.0	65,118.6	63,258.9	0.014	0.0000
G. morhua (m)	*G. macrocephalus*	*Mel. aeglefinus*	1,381,730.0	48,034.4	46,407.9	0.017	0.0000
G. morhua (m)	*G. ogac*	*Mel. aeglefinus*	1,378,670.0	47,735.4	45,982.6	0.019	0.0000
G. morhua (m)	*G. chalcogrammus*	*Mel. aeglefinus*	1,630,450.0	32,523.8	31,345.2	0.018	0.0000
G. morhua (m)	*G. morhua* (s)[2]	*Mel. aeglefinus*	2,128,480.0	6,106.4	5,964.9	0.012	0.0360
Mel. aeglefinus	*Mer. merlangus*	*B. saida*	524,569.0	109,421.0	104,705.0	0.022	0.0000
Mel. aeglefinus	*Mer. merlangus*	*A. glacialis*	526,258.0	103,925.0	99,293.4	0.023	0.0000
Mel. aeglefinus	*Mer. merlangus*	*G. macrocephalus*	535,103.0	106,401.0	101,740.0	0.022	0.0000
Mel. aeglefinus	*Mer. merlangus*	*G. ogac*	532,068.0	105,833.0	101,330.0	0.022	0.0000
Mel. aeglefinus	*Mer. merlangus*	*G. chalcogrammus*	534,655.0	107,694.0	101,792.0	0.028	0.0000
Mel. aeglefinus	*Mer. merlangus*	*G. morhua* (s)	544,694.0	110,805.0	103,491.0	0.034	0.0000
Mel. aeglefinus	*Mer. merlangus*	*G. morhua* (m)	544,885.0	110,788.0	103,421.0	0.034	0.0000
G. morhua (m)	*A. glacialis*	*B. saida*	170,613.0	280,258.0	131,776.0	<u>0.360</u>	0.0000
G. morhua (m)	*G. macrocephalus*	*B. saida*	461,022.0	87,150.0	74,312.0	0.080	0.0000
G. morhua (m)	*G. ogac*	*B. saida*	463,606.0	86,146.6	73,616.9	0.078	0.0000
G. morhua (m)	*G. chalcogrammus*	*B. saida*	699,019.0	51,353.8	42,041.8	0.100	0.0000
G. morhua (m)	*G. morhua* (s)	*B. saida*	1,178,120.0	6,965.9	6,699.1	0.020	0.0004
G. morhua (m)	*G. macrocephalus*	*A. glacialis*	425,364.0	85,291.4	71,895.4	0.085	0.0000
G. morhua (m)	*G. ogac*	*A. glacialis*	428,397.0	84,501.5	71,280.5	0.085	0.0000
G. morhua (m)	*G. chalcogrammus*	*A. glacialis*	669,250.0	50,390.8	40,088.8	0.114	0.0000
G. morhua (m)	*G. morhua* (s)	*A. glacialis*	1,159,190.0	6,748.2	6,422.2	0.025	0.0000
G. morhua (m)	*G. chalcogrammus*	*G. macrocephalus*	337,874.0	74,069.5	55,581.5	0.143	0.0000
G. morhua (m)	*G. morhua* (s)	*G. macrocephalus*	820,109.0	7,930.4	7,495.9	0.028	0.0000
G. macrocephalus	*G. ogac*	*G. chalcogrammus*	941,162.0	9,380.4	7,456.4	0.114	0.0000
G. macrocephalus	*G. ogac*	*G. morhua* (s)	980,086.0	13,956.1	7,797.4	<u>0.283</u>	0.0000
G. macrocephalus	*G. ogac*	*G. morhua* (m)	980,709.0	14,167.9	7,875.9	<u>0.285</u>	0.0000
G. morhua (m)	*G. morhua* (s)	*G. chalcogrammus*	532,010.0	10,099.0	9,619.8	0.024	0.0000

[1]Migratory *Gadus morhua* individual LOF1103Z24 sampled at the Lofoten islands
[2]Stationary *Gadus morhua* individual LOF1106Z11 sampled at the Lofoten islands

Supplementary References

1. Árnason, E. & Halldórsdóttir, K. Codweb: Whole-genome sequencing uncovers extensive reticulations fueling adaptation among Atlantic, Arctic, and Pacific gadids. *Sci. Adv.* **5,** eaat8788 (2019).
2. Solís-Lemus, C. & Ané, C. Inferring phylogenetic networks with maximum pseudolikelihood under incomplete lineage sorting. *PLOS Genet.* **12,** e1005896 (2016).
3. Saitou, N. & Nei, M. The neighbor-joining method: a new method for reconstructing phylogenetic trees. *Mol. Biol. Evol.* **4,** 406–425 (1987).
4. Paradis, E., Claude, J. & Strimmer, K. APE: Analyses of Phylogenetics and Evolution in R language. *Bioinformatics* **20,** 289–290 (2004).
5. Coulson, M. W., Marshall, H. D., Pepin, P. & Carr, S. M. Mitochondrial genomics of gadine fishes: implications for taxonomy and biogeographic origins from whole-genome data sets. *Genome* **49,** 1115–1130 (2006).
6. Teletchea, F., Laudet, V. & Hänni, C. Phylogeny of the Gadidae (sensu Svetovidov, 1948) based on their morphology and two mitochondrial genes. *Mol. Phylogenet. Evol.* **38,** 189–199 (2006).
7. Owens, H. L. Evolution of codfishes (Teleostei: Gadinae) in geographical and ecological space: evidence that physiological limits drove diversification of subarctic fishes. *J. Biogeogr.* **42,** 1091–1102 (2015).
8. Malmstrøm, M. *et al.* Evolution of the immune system influences speciation rates in teleost fishes. *Nat. Genet.* **48,** 1204–1210 (2016).
9. Hughes, L. C. *et al.* Comprehensive phylogeny of ray-finned fishes (Actinopterygii) based on transcriptomic and genomic data. *Proc. Natl. Acad. Sci. U.S.A.* **5,** 201719358 (2018).
10. Tørresen, O. K. *et al.* An improved genome assembly uncovers prolific tandem repeats in Atlantic cod. *BMC Genomics* **18,** 95 (2017).
11. Günther, T. & Nettelblad, C. The presence and impact of reference bias on population genomic studies of prehistoric human populations. *PLOS Genet.* **15,** e1008302 (2019).
12. Sheng, G.-L. *et al.* Paleogenome reveals genetic contribution of extinct giant panda to extant populations. *Curr. Biol.* **29,** 1695–1700.e6 (2019).
13. Kirubakaran, T. G. *et al.* Two adjacent inversions maintain genomic differentiation between migratory and stationary ecotypes of Atlantic cod. *Mol. Ecol.* **25,** 2130–2143 (2016).
14. Kucuk, E. *et al.* Kollector: transcript-informed, targeted de novo assembly of gene loci. *Bioinformatics* **33,** 1782–1788 (2017).
15. Roth, O. *et al.* Evolution of male pregnancy associated with remodeling of canonical vertebrate immunity in seahorses and pipefishes. *Proc. Natl. Acad. Sci. U.S.A.* **117,** 9431–9439 (2020).

16. Nguyen, L.-T., Schmidt, H. A., Von Haeseler, A. & Minh, B. Q. IQ-TREE: A fast and effective stochastic algorithm for estimating maximum-likelihood phylogenies. *Mol. Biol. Evol.* **32,** 268–274 (2015).

17. Roa-Varon, A. & Orti, G. Phylogenetic relationships among families of Gadiformes (Teleostei, Paracanthopterygii) based on nuclear and mitochondrial data. *Mol. Phylogenet. Evol.* **52,** 688–704 (2009).

18. Green, R. E. *et al.* A draft sequence of the Neandertal genome. *Science* **328,** 710–722 (2010).

19. Durand, E. Y., Patterson, N., Reich, D. & Slatkin, M. Testing for ancient admixture between closely related populations. *Mol. Biol. Evol.* **28,** 2239–2252 (2011).

20. Korneliussen, T. S., Albrechtsen, A. & Nielsen, R. ANGSD: Analysis of next generation sequencing data. *BMC Bioinformatics* **15,** 356 (2014).

21. Kelleher, J., Etheridge, A. M. & McVean, G. Efficient coalescent simulation and genealogical analysis for large sample sizes. *PLOS Comput. Biol.* **12,** e1004842 (2016).

22. Speidel, L., Forest, M., Shi, S. & Myers, S. R. A method for genome-wide genealogy estimation for thousands of samples. *Nat. Genet.* 1321–1329 (2019).

23. Tuttle, E. M. *et al.* Divergence and functional degradation of a sex chromosome-like supergene. *Curr. Biol.* **26,** 344–350 (2016).

24. Edelman, N. B. *et al.* Genomic architecture and introgression shape a butterfly radiation. *Science* **366,** 594–599 (2019).

25. Müller, N. F., Ogilvie, H. A., Zhang, C., Drummond, A. & Stadler, T. Inference of species histories in the presence of gene flow. *bioRxiv.* doi:10.1101/348391 (2018).

26. Bouckaert, R. R. *et al.* BEAST 2.5: An advanced software platform for Bayesian evolutionary analysis. *PLOS Comput. Biol.* **15,** e1006650 (2019).

27. Malinsky, M., Matschiner, M. & Svardal, H. Dsuite - Fast D-statistics and related admixture evidence from VCF files. *Mol. Ecol. Resour.* **19,** 1655 (2020).

28. Malinsky, M. *et al.* Genomic islands of speciation separate cichlid ecomorphs in an East African crater lake. *Science* **350,** 1493–1498 (2015).

29. Martin, S. H., Davey, J. W. & Jiggins, C. D. Evaluating the use of ABBA-BABA statistics to locate introgressed loci. *Mol. Biol. Evol.* **32,** 244–257 (2015).

30. Ronco, F. *et al.* Drivers and dynamics of a massive adaptive radiation in cichlid fishes. *Nature* **589,** 76–81 (2021).

31. Arcila, D. *et al.* Genome-wide interrogation advances resolution of recalcitrant groups in the tree of life. *Nat. Ecol. Evol.* **1,** 1–10 (2017).

32. Barth, J. M. I. *et al.* Stable species boundaries despite ten million years of hybridization in tropical eels. *Nat. Commun.* **11,** 1433 (2020).

33. Juric, I., Aeschbacher, S. & Coop, G. The strength of selection against Neanderthal introgression. *PLOS Genet.* **12,** e1006340 (2016).

34. Schumer, M. *et al.* Natural selection interacts with recombination to shape the evolution of hybrid genomes. *Science* **360,** 656–660 (2018).

35. Matute, D. R. *et al.* Rapid and predictable evolution of admixed populations between two *Drosophila* species pairs. *Genetics* **214,** 211–230 (2020).

36. Kirubakaran, T. G. *et al.* A nanopore based chromosome-level assembly representing Atlantic cod from the Celtic Sea. *G3,* g3.401423.2020 (2020).

37. Musilova, Z. *et al.* Vision using multiple distinct rod opsins in deep-sea fishes. *Science* **364,** 588–592 (2019).

38. The International HapMap Consortium. The International HapMap Project. *Nature* **426,** 789–796 (2003).

39. Tørresen, O. K. *et al.* Genomic architecture of haddock (*Melanogrammus aeglefinus*) shows expansions of innate immune genes and short tandem repeats. *BMC Genomics* **19,** 240 (2018).

40. Barth, J. M. I. *et al.* Disentangling structural genomic and behavioural barriers in a sea of connectivity. *Mol. Ecol.* **87,** 449 (2019).

41. Ruegg, K., Anderson, E. C., Boone, J., Pouls, J. & Smith, T. B. A role for migration-linked genes and genomic islands in divergence of a songbird. *Mol. Ecol.* **23,** 4757–4769 (2014).

42. Ogilvie, H. A., Bouckaert, R. R. & Drummond, A. J. StarBEAST2 brings faster species tree inference and accurate estimates of substitution rates. *Mol. Biol. Evol.* **34,** 2101–2114 (2017).
