# Supplementary material for: Supergene origin and maintenance in Atlantic cod
Source: Nat Ecol Evol. 2022 Feb 17;6(4):469–81. doi: 10.1038/s41559-022-01661-x (PMC8986531; doi:10.1038/s41559-022-01661-x)
Supplement: Supplementary file 2 — Reporting Summary [file 41559_2022_1661_MOESM2_ESM.pdf]

nature portfolio

Corresponding author(s): Michael Matschiner, Sissel Jentoft

Last updated by author(s): Dec 13, 2021

Reporting Summary

Nature Portfolio wishes to improve the reproducibility of the work that we publish. This form provides structure for consistency and transparency in reporting. For further information on Nature Portfolio policies, see our Editorial Policies and the Editorial Policy Checklist.

Statistics

For all statistical analyses, confirm that the following items are present in the figure legend, table legend, main text, or Methods section.

n/a	Confirmed	
☐	☒	The exact sample size (*n*) for each experimental group/condition, given as a discrete number and unit of measurement
☐	☒	A statement on whether measurements were taken from distinct samples or whether the same sample was measured repeatedly
☐	☒	The statistical test(s) used AND whether they are one- or two-sided<br>*Only common tests should be described solely by name; describe more complex techniques in the Methods section.*
☒	☐	A description of all covariates tested
☐	☒	A description of any assumptions or corrections, such as tests of normality and adjustment for multiple comparisons
☐	☒	A full description of the statistical parameters including central tendency (e.g. means) or other basic estimates (e.g. regression coefficient) AND variation (e.g. standard deviation) or associated estimates of uncertainty (e.g. confidence intervals)
☐	☒	For null hypothesis testing, the test statistic (e.g. *F*, *t*, *r*) with confidence intervals, effect sizes, degrees of freedom and *P* value noted<br>*Give P values as exact values whenever suitable.*
☐	☒	For Bayesian analysis, information on the choice of priors and Markov chain Monte Carlo settings
☒	☐	For hierarchical and complex designs, identification of the appropriate level for tests and full reporting of outcomes
☒	☐	Estimates of effect sizes (e.g. Cohen's *d*, Pearson's *r*), indicating how they were calculated

Our web collection on statistics for biologists contains articles on many of the points above.

Software and code

Policy information about availability of computer code

Data collection	No software was used in the data collection process. Sequencing data were generated through Illumina HiSeq sequencing at the Norwegian Sequencing Centre, Oslo, Norway.
Data analysis	Construction of gadMor_Stat assembly: Celera Assembler v.8.3rc2, Quiver v.0.9.0, BWA MEM v.0.7.12-r1039, SAMtools v.1.10, Pilon v.1.16, BUSCO v.5.0<br>Whole-genome sequencing and population-level variant calling: BWA MEM v.0.7.17, SAMtools v.1.9, Picard tools v.2.18.27, GATK v.4.1.2.0, BCFtools v.1.9<br>Delimiting high-LD regions associated with inversions: VCFtools v.0.1.14, PLINK v.1.90b3b<br>Contig mapping: BLASTN v.2.2.29<br>Threeway whole-genome alignment: MASA-CUDAlign v.3.9.1.1024, LASTZ v.1.0.4, RepeatMasker v.1.0.8<br>Estimating divergence times of Gadinae: Kollector v.1.0.1, BMGE v.1.1, RAxML v.8.2.4, Concaterpillar v.1.7.2, PAUP* v.4.0, BEAST v.2.6, Dsuite v.0.1.r3, IQ-TREE v.1.6.8<br>Estimating divergence times, demography, and gene flow among Gadus morhua populations: Relate v.1.1.2, BEAGLE v.5.1, msprime v.0.7.4.<br>All custom code is available from https://github.com/mmatschiner/supergenes.

For manuscripts utilizing custom algorithms or software that are central to the research but not yet described in published literature, software must be made available to editors and reviewers. We strongly encourage code deposition in a community repository (e.g. GitHub). See the Nature Portfolio guidelines for submitting code & software for further information.

Data

Policy information about availability of data

All manuscripts must include a data availability statement. This statement should provide the following information, where applicable:

- Accession codes, unique identifiers, or web links for publicly available datasets
- A description of any restrictions on data availability
- For clinical datasets or third party data, please ensure that the statement adheres to our policy

The gadMor_Stat assembly (ENA accession GCA_905250895) and read data for all Gadus morhua specimens listed in Supplementary Table 8 are deposited on ENA with project number PRJEB43149. Alignment files, SNP datasets in PED and VCF format, and input and output of phylogenetic analyses are available from Zenodo (doi: 10.5281/zenodo.4560275).

Field-specific reporting

Please select the one below that is the best fit for your research. If you are not sure, read the appropriate sections before making your selection.

☐ Life sciences ☐ Behavioural & social sciences ☒ Ecological, evolutionary & environmental sciences

For a reference copy of the document with all sections, see nature.com/documents/nr-reporting-summary-flat.pdf

Ecological, evolutionary & environmental sciences study design

All studies must disclose on these points even when the disclosure is negative.

Study description	We generate a new sequence assembly and population genomic data for Atlantic cod and analyze this data together with published data of the same species and closely related species.
Research sample	Sampling for whole-genome sequencing included 22 specimens of Atlantic cod (Gadus morhua), collected at eight localities. Samples were collected with hand line, gillnet, longline, and trawling, as listed in Supplementary Table 8. The eight sampling localities were chosen to cover the distribution of Atlantic cod in the North Atlantic, with two localities (Newfoundland and Labrador) in the Northwest Atlantic and six localities in the Northeast Atlantic (Iceland, Lofoten, Møre, Bornholm Basin, Kiel Bight, and Suffolk). Stationary Atlantic cod individuals were collected from all localities, and migratory Atlantic cod were caught from all those localities where these are known to occur during the spawning season (Newfoundland, Iceland, Lofoten, and Møre). In addition to these 22 individuals, we included publicly available data for 11 further species of the family Gadidae. These species include the following:<br>Brosme brosme<br>Gadiculus argenteus<br>Trisopterus minutus<br>Pollachius virens<br>Melanogrammus aeglefinus<br>Merlangius merlangus<br>Boreogadus saida<br>Arctogadus glacialis<br>Gadus macrocephalus<br>Gadus ogac<br>Gadus chalcogrammus<br>For these species, genome assemblies were downloaded from Dryad (datadryad.org) and read data were taken from the EBI (ebi.ac.uk) database (full links to all datasets are provided in Supplementary Tables 4 and 5).
Sampling strategy	The samples used in this study were part of a larger dataset and were selected from this larger dataset based on sampling locality, ecotype (migratory or stationary), and data completeness. The sample used to produce the gadMor_Stat genome assembly was selected so that its supergene haplotypes on linkage groups 1, 2, 7, and 12 were complementary to those of the existing gadMor2 genome assembly. The sample size used for this study was chosen to cover the distribution of Atlantic cod in the North Atlantic, while considering run times of computationally demanding analyses, which scale exponentially with the number of included samples.
Data collection	Whole-genome sequencing data were generated by PacBio and Illumina HiSeq sequencing on the Pacific Biosciences RS II and Illumina HiSeq2500 platforms, respectively, operated by the Norwegian Sequencing Centre (NSC), Oslo, Norway. Genome sequencing libraries were prepared by NSC staff members.
Timing and spatial scale	Samples were collected between 2011 and 2015 as described in Supplementary Table 8. For the Norwegian localities where both migratory and stationary individuals co-occur during the spawning season, samples were collected during and outside of the spawning season to adequately sample both ecotypes. As stated above and in Supplementary Table 8, the spatial scale encompasses the North Atlantic.
Data exclusions	As stated above, samples used in this study are part of a larger dataset and were selected based on sampling locality, ecotype (migratory or stationary), and data completeness.
Reproducibility	To enable the reproduction of our result by other researchers, we provide all datasets, analysis code, and input files for certain programs on https://github.com/mmatschiner/supergenes. As part of our study, reproducibility was confirmed for specific analyses, such as all Bayesian analyses with BEAST 2 or SNAPP, for which we performed two replicate analyses with each dataset, and

additionally sets of analyses with different datasets, that all supported the same result. Overall, all attempts at replication were successful, and none of the results could not be reproduced.

Randomization	Atlantic cod samples were determined to be migratory or stationary based on otholith shape for all those samples for which otolith shape was available. For samples from Iceland and Newfoundland, otolith shape was not available, and these samples were considered migratory or stationary based on their haplotype of the supergene on linkage group 1.
Blinding	Tree inference was not constrained according to previously available taxonomic information; thus, the applied methods were blind to taxonomic groupings, some of which have been established beyond doubt by past research. The inferred trees agreed with all established groupings.

Did the study involve field work? ☒ Yes ☐ No

Field work, collection and transport

Field conditions	As state above and described in Supplementary Table 8, Atlantic cod samples were collected at different times of the year, between March and September. The conditions varied with the season, but should not be relevant for the study besides the effect of the spawning season, during which migratory cod is present at Newfoundland, Iceland, Lofoten, and Møre.
Location	Coordinates for all sampling localities are provided in Supplementary Table 8. All sampling took place on the sea or from the coast, so the elevation of all localities was at sea level. Water depth presumably varied but was not recorded.
Access & import/export	All sampling and sample transport was performed in accordance with local, national, and international law.
Disturbance	Disturbance of the environment was not recorded but expected to be minimal with all applied sampling methods.

Reporting for specific materials, systems and methods

We require information from authors about some types of materials, experimental systems and methods used in many studies. Here, indicate whether each material, system or method listed is relevant to your study. If you are not sure if a list item applies to your research, read the appropriate section before selecting a response.

Materials & experimental systems

n/a	Involved in the study	
☒	☐	Antibodies
☒	☐	Eukaryotic cell lines
☒	☐	Palaeontology and archaeology
☐	☒	Animals and other organisms
☒	☐	Human research participants
☒	☐	Clinical data
☒	☐	Dual use research of concern

Methods

n/a	Involved in the study	
☒	☐	ChIP-seq
☒	☐	Flow cytometry
☒	☐	MRI-based neuroimaging

Animals and other organisms

Policy information about studies involving animals; ARRIVE guidelines recommended for reporting animal research

Laboratory animals	Study did not involve laboratory animals
Wild animals	Atlantic cod individuals were caught as described above, and humanely sacrificed immediately after catching.
Field-collected samples	None of the field-collected samples were taken to the lab.
Ethics oversight	We always strive to limit the effect of our sampling needs on populations and individuals. All samples used in this study have been collected in a responsible manner: i) in connection to research surveys (as part of larger hauls for stock assessments) or ii) by commercial fisheries (obtained as byproduct of conventional business practice). The fish were humanely sacrificed before sampling in accordance with the guidelines set by national and international animal welfare laws (e.g. www.norecopa.no), and thus no specific legislation were needed. Sampling was performed prior to the respective countries signed the the Nagoya Protocol (for instance for UK the date of accession was set to May 22nd 2016).

Note that full information on the approval of the study protocol must also be provided in the manuscript.
